# A high-resolution map of coastal vegetation for two Arctic Alaskan parklands: An object-oriented approach with point training data

**DOI:** 10.1371/journal.pone.0273893

**Published:** 2022-08-31

**Authors:** Celia J. Hampton-Miller, Peter N. Neitlich, David K. Swanson

**Affiliations:** 1 Arctic Inventory & Monitoring Network, National Park Service, Fairbanks, Alaska, United States of America; 2 Arctic Inventory & Monitoring Network, National Park Service, Anchorage, Alaska, United States of America; Universite de Nantes, FRANCE

## Abstract

Bering Land Bridge National Preserve and Cape Krusenstern National Monument in northwest Alaska have approximately 1600 km of predominantly soft-sediment coastlines along the Chukchi Sea, a shallow bay of the Arctic Ocean. Over the past decade, marine vessel traffic through the Bering Strait has grown exponentially to take advantage of new ice-free summer shipping routes, increasing the risk of oil spills in these fragile ecosystems. We present a high-resolution coastal vegetation map to serve as a baseline for potential spill response, restoration, and change detection. We segmented 663 km^2^ of high-resolution multispectral satellite images by the mean-shift method and collected 40 spectral, topographic and spatial variables per segment. The segments were classified using photo-interpreted points as training data, and verified with field based plots. Digitizing points, rather than polygons, and intersecting them with the segmentation allows for rapid collection of training data. We classified the map segments using Random Forest because of its high accuracy, computational speed, and ability to incorporate non-normal, high-dimensional data. We found creating separate classification models by each satellite scene gave highly similar results to models combining the entire study area, and that reducing the number of variables had little impact on accuracy. A unified, study area-wide Random Forest model for both parklands produced the highest accuracy of various models attempted. We mapped 18 distinct classes, with an out-of-bag error of 11.6%, resulting in an improvement to the past per-pixel classification of this coast, and in higher spatial and vegetation classification resolution. The resulting map demonstrates the utility of our point-based method and provides baseline data for incident preparedness and change detection. Elevation is highly correlated with the ordination of the vegetation types, and was the most important variable in all tested classification models. The vegetation classification brings together the largest amount of vegetation data for the Chukchi Sea coast yet documented.

## Introduction

**Fig 1:**
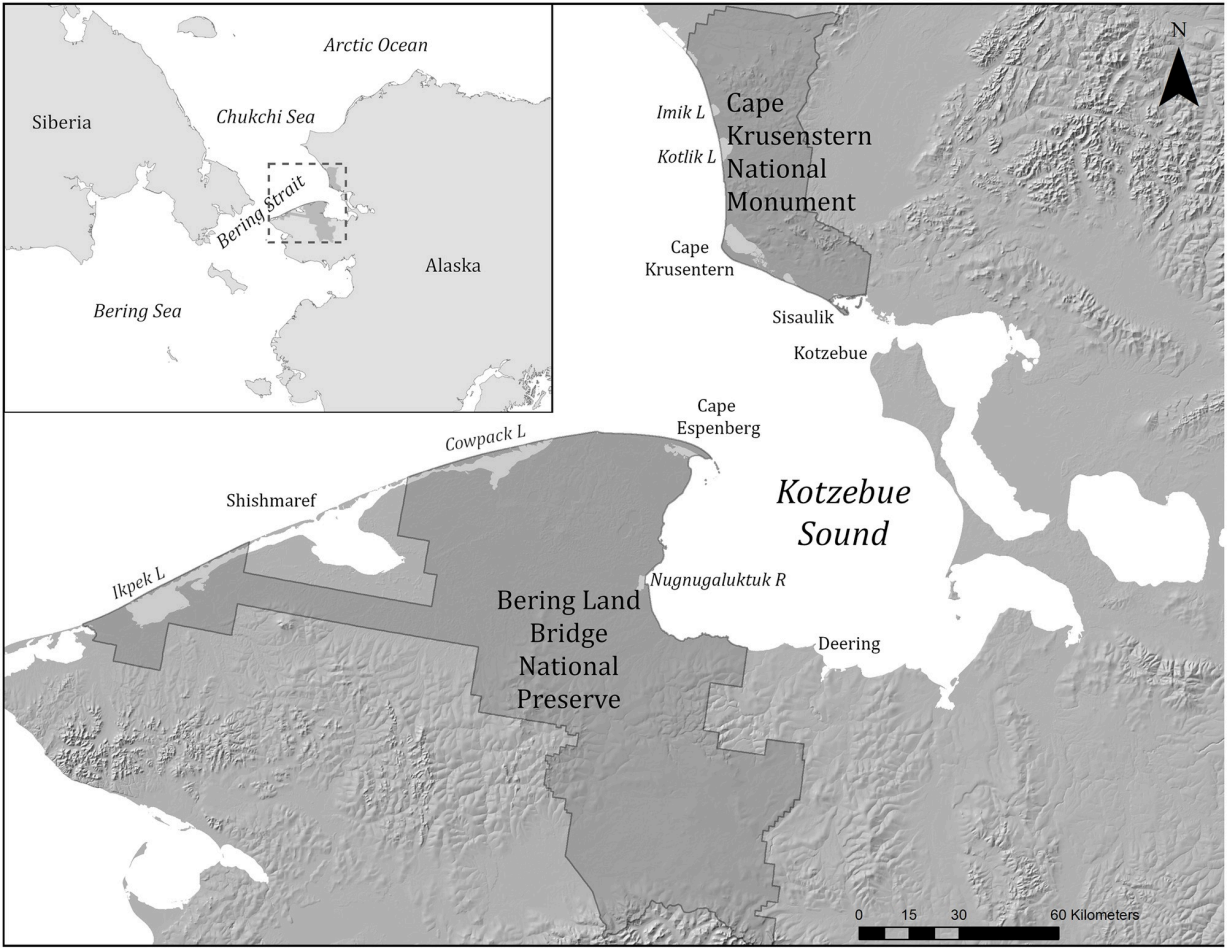
Study area map. 1A. Location of the Chukchi Sea. 1B. Location of the two parks and relevant coastal landmarks. Elevation data USGS 30 ARC-second Global Elevation Data, GTOPO30. Research Data Archive at the National Center for Atmospheric Research, Computational and Information Systems Laboratory. https://doi.org/10.5065/A1Z4-EE71. CC By 4.0.

The dynamic, soft-sediment Arctic coastal ecosystems of northwest Alaska offer important ecological services and habitat for a wide variety of Arctic and migratory species [1]. These shorelines include vast and shallow lagoons with fractal-patterned interiors, large estuaries teeming with waterbirds, barrier islands, sandy capes, salt marshes, mudflats, brackish wetlands, and the world’s northernmost eelgrass beds [2]. Like those of eastern North America before European contact, the northwest Arctic shorelines are wild, productive, and extensive. These areas are globally significant for a variety of life forms; in particular the lagoon systems and Nugnugaluktuk Estuary (Fig 1) of BELA are identified as Global Important Bird Areas by the Audubon Society [1]. These lagoons also serve as important habitat for a diversity of fish and bird species, including whitefishes and other salmonids, which are important subsistence resources in the region [3].

The enabling legislation [4] of both Bering Land Bridge National Preserve (BELA; Fig 1) and Cape Krusenstern National Monument (CAKR; Fig 1) mandates protection of subsistence resources, plant communities, coastal formations, migratory bird habitat, fish and wildlife, and additionally archaeological sites in CAKR. CAKR was established in large part to preserve the history of ancient human settlements along the coast which arose in concert with the rich coastal resources, and which provide a detailed archaeological chronosequence of human habitation spanning over 5000 years [5, 6]. The Arctic coast continues to be home to Native Iñupiaq communities, for whom marine mammal and fish harvests are the center of their statutorily protected way of life.

Arctic sea ice volume, extent and duration have declined dramatically for decades [7], leaving the coast increasingly vulnerable to fall and winter storms. The tidal range along the northwest Alaskan coast is small, estimated at a 30 cm daily average range [8], but storm surge and atmospheric pressure can drive water levels several meters beyond mean sea level [9, 10]. Reduction in sea ice means a winter storm now has greater potential to drive oil or other contaminants deep into the sensitive habitats described in this study.

In the summer months, the Arctic ice pack is now sufficiently far north to allow for passage of vessels via both the Northern Sea Route (above Siberia) and the Northwest Passage (through the Canadian Archipelago to Greenland). Over the past decade, vessel traffic has grown exponentially through the Bering Strait with the retreat of summer sea ice and now includes a significant number of tankers and cargo ships [11]. At the same time, planning and initial stages of development for oil and gas extraction have progressed in the National Petroleum Reserve-Alaska, as have plans for the development of deepwater ports at Nome and Cape Blossom (near Kotzebue). These developments now place BELA and CAKR at risk of a marine incident without adequate pre-disturbance vegetation mapping.

### Remote sensing and vegetation classification

Prior to this study the best available vegetation map of the study area was a 30-m per-pixel raster map derived from supervised classification of Landsat data, covering all of the five National Park Service units in northern Alaska, produced by Jorgenson et al. [12]. This map was based on extensive fieldwork, and maps 44 detailed ecotypes, of which 5 were specific coastal types. Overall map accuracy was estimated to be between 65 and 80%.

National Parks still primarily create vegetation classification maps via a combination of fieldwork and hand-drawn polygons based on aerial imagery (e.g. [13–16]). These methods can be labor intensive, and do not allow for error estimation without further costly fieldwork. Remote sensing vegetation classification has advantages—less time investment, lower cost, and multiple levels of error estimation. Per-pixel methods, where each pixel of aerial imagery is individually classified, frequently result in a ‘salt-and-pepper’ effect [17]. Grouping pixels into segments, or objects, allows more homogenous clusters to be classified, as well as the collection of object-level traits such as size and shape [17–19]. Geographic object-oriented landcover classification has been widely used in urban and agricultural areas, where the borders between types are clearly delimited and segment shape is often a highly useful parameter [20, 21]. Its use is undeveloped landscapes is often limited to classifying a handful of broad landcover classes; e.g. forested vs unforested [22, 23]. Mapping of higher resolution landcover classes (>8 classes) is also typically divided by functional group (e.g. [24]), or physiognomy (e.g. [25]), not plant associations.

To assist in park preparedness, we have produced a vegetation classification and detailed map of the coastal vegetation communities. The Jorgenson et al. [12] raster map includes only two brackish water vegetation classes—Coastal Brackish Sedge-Grass Meadow and Coastal Brackish Willow Shrub. In reality, coastal salt marsh is among the most productive habitats along the BELA and CAKR coasts and represents a complex mosaic of vegetation types, each of which hosts different bird species using these areas for nesting, foraging and pre-migration staging. As most of the BELA coast falls into a multi-species bird hotspot represented as a global Important Bird Area, fine-scale delineation of habitat types helps discriminate habitats into polygons used by dozens of species for different purposes at different times of the year [26]. These types include lagoon interior marshes at different tidal elevations, estuarine salt marshes at different elevation and salinity, and several other halophytic sedge and shrub-dominated classes. By mapping vegetation at a higher resolution than Jorgenson et al. [12], we hope to enable far more insight aimed at potential uses for spill response, post-spill restoration, Natural Resource Damage Assessment (NRDA), and the scientific community at large for uses in vegetation monitoring, climate change, and sea level rise detection. In the event of an oil spill, a fine-scale habitat baseline is the single most valuable tool for response, restoration and NRDA [1]. We aimed to map at least 10 distinct coastal vegetation types, with a minimum polygon size of 200 m^2^ and smooth polygon boundaries when displayed at 1:10,000 scale. To meet NPS standards for vegetation classification, we mapped USNVC vegetation types. NPS minimum accuracy for vegetation classification is 60% [26]; our target accuracy was 80% as is typical of similar object-oriented classifications [22, 24, 27].

## Methods

### Study area

**Fig 2:**
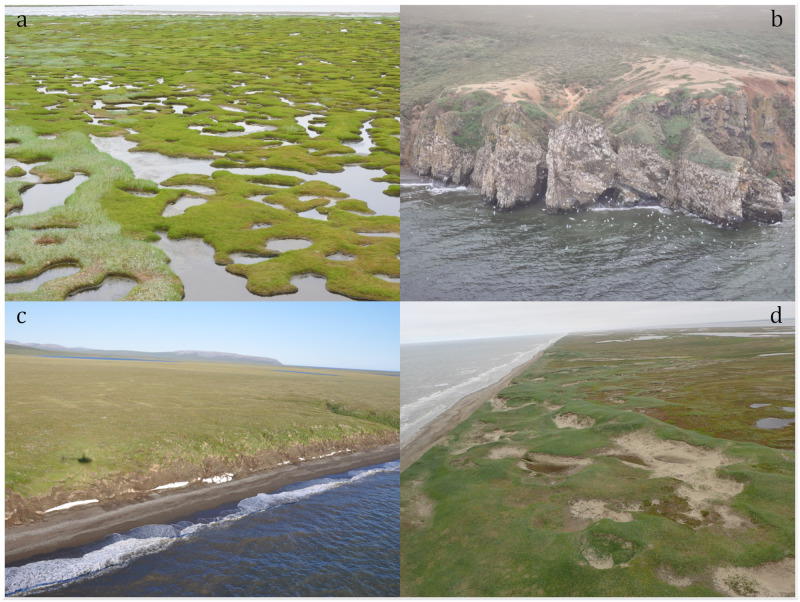
Examples of coastal geomorphology features. A. Patterned salt marsh at the mouth of the Nugnugaluktuk Estuary, eastern BELA. B. Bedrock outcrop on the coast of Goodhope Bay, eastern BELA. C. Ice-wedge polygon tundra bluff along the beach north of Cape Krusenstern Lagoon, CAKR. D. Active and stabilized sand dunes on the barrier island of Cowpack Lagoon, northern BELA. Photo A NPS, public domain, B, C & D photos from ShoreZone [33], licensed CC By 3.0.

Coasts of both parks lie along the Chukchi Sea, an embayment of the Arctic Ocean north of the Bering Strait (Fig 1). The Chukchi Sea is a shallow continental shelf, averaging 50 m deep. The primary coastal features of both parks’ lands are gravelly or sandy barrier island complexes, backed by lagoons [28, 29]. The beach ridges of both coasts initiated nearly 4000 years BP as the sea level stabilized [30, 31]. Lagoons and estuaries are bordered by salt marshes (Fig 2A). Other parts of the coast are ice-rich permafrost bluffs with a narrow band of beach (Fig 2B). These coastal bluffs are found where the lagoon and barrier complexes are absent: in CAKR most notably along much of the west-facing coast, and in BELA near Kitluk River and along the coast of Kotzebue Sound. Bedrock outcrops occur along the coast in Goodhope Bay (Fig 2C) and Ugrurak Bluff, north of Tasaychek Lagoon in Cape Krusenstern. The mean annual temperature along the BELA and CAKR coasts is -5° C [32].

### Field methods

We sampled 362 vegetation plots in Bering Land Bridge National Preserve and Cape Krusenstern National Monument in the summers of 2018 and 2019. Due to the remote, roadless nature of these parklands, access was by helicopter and foot. Using satellite imagery, we selected sampling areas to maximize high spectral and landform diversity and to minimize flight time and expense. Plots were arrayed subjectively, attempting to capture as much of the ecological variation as possible in the imagery across a broad geographical area. On the ground, plots were located within areas of homogeneous vegetation, as well as mosaics (multiple types within 200 m^2^) and gradual eco-tones, but plot locations that spanned distinct boundaries between types were avoided. Our intent was to obtain a representative sample of the vegetation while avoiding plot locations that were likely to mix data from plant assemblages that were likely to represent more than one type in the final classification. Plots were circular with an 8-meter radius (200 m^2^). All vascular plants with at least 1% cover within the plot were identified to species in the field or collected for later identification. Plants below 1% cover were identified in the field to species or genus. Taxonomy follows the vascular plant inventory of the National Park Service (NPS) Arctic Inventory and Monitoring Network (ARCN) [34]. Mosses and lichens were identified to species or species group, following the ARCN Vegetation sampling protocol [35]. For all species in the plot, ocular estimates of cover were made using the following five classes: 0:<1%, 1:1–5%, 2:5–25%, 3:25–50%, 4:50–75%, 5:>75%, a modified Daubenmire scale [36]. GPS coordinates were collected at plot center on Trimble Geo XH 6000 or Geo 7X model receivers, and post-process corrected to the Kotzebue CORS reference station. The mean horizontal error was 30 cm, with 99% in the range of 10–50 cm. Electrical conductivity (EC) in units of μS/m, an estimate of salinity, was measured at plot center with a soil probe (Hanna Instruments Direct Soil EC Tester). Photos were taken in each cardinal direction. Cover of functional groups (e.g. shrubs, forbs, graminoids) was estimated visually. Post-fieldwork, a hydric index—a proxy for site wetness—was calculated by weighted averaging using wetland species weights from the Federal Interagency Committee for Wetland Delineation [37].

Along with the 362 plots collected in the summers of 2018 and 2019 (two of which were non-vegetated), we incorporated 37 coastal plots collected in 2003 by Jorgenson et al. [12] and 35 coastal plots established in 2013 as part of the ARCN Vegetation Node Sampling protocol [35]. These data, measured in percent cover, were converted to the above cover categories for comparison with our data, and synonymy was standardized to the NPS Arctic Network plant species list [34]. Non-vascular plant diversity was reduced to the species list collected for the ARCN vegetation protocols [35]. To be comparable to the EC units collected in this study EC values for Jorgenson et al. [12] plots were truncated to 4000 (the maximum value read on our instruments). EC was not collected for the 35 ARCN plots; we imputed these values through a k-nearest neighbor model using the R package VIM [38] (data available: [39]).

### Classification methods

Our classification goal was to balance the recognition of as many discrete vegetation types as possible with the ability to distinguish these types via photo interpretation and automated classification on the map product. Our final goal of the classification methods was the description of vegetation types that can be keyed dichotomously, and we used both analytical and subjective tools to separate vegetation types.

We initially attempted to classified plots via previously existing vegetation classification schemes [12, 40]. Some plots clearly fit type descriptions, others were lumped into overly broad categories, and others did not match any described vegetation types. We assigned plots to types which matched descriptions; these included Halophytic salt marsh, Dunegrass beach meadow, Crowberry tundra and Freshwater wet sedge meadow [12, 40]. We used nonmetric multidimensional scaling (NMS) ordination [41] iteratively to test whether these pre-existing types were coherent in species space, whether further subdivisions were possible, and how unlabeled plots clustered.

We began our analyses with a matrix of 432 plots and 302 species. A Bray-Curtis dissimilarity matrix was used as the basis for ordinations. As ordinations are reductions in dimensionality via co-occurring species, plots with only one species do not ordinate. Thus, plots assigned to the predominately monoculture classes comprised of *Arctophila fulva*, *Carex lyngbyei*, *Carex saxatilis* and *Hippuris tetraphylla* were removed from ordination analyses, as were outliers, defined as plots more than two standard deviations above the mean Bray-Curtis dissimilarity [42] to all other plots [43]. These plots were statistical as well as ecological outliers. Thirteen outlier plots and 31 monoculture type plots were removed from the ordination analyses. All species occurrences with less than 1% cover, and species with less than three plot occurrences total, were removed from the dataset, leaving a matrix of 386 plots and 117 species. Preliminary ordinations were used to find whether further subdivisions of types were coherent in species space. Outliers and boundaries between types were distinguished using sorted table analyses [44, 45].

Non-metric multidimensional scaling (NMS) ordinations were run via the R package ‘vegan’ using the function ‘metaMDS’ [46]. A Bray-Curtis dissimilarity matrix of the community data (reduced as described above) was input with the following parameters: 2 dimensions, with 250 runs of data, a maximum number of random starts 500 and maximum iterations 999. The ordination was constrained to 2 dimensions based on a step-down in dimensionality. Previous ordination positions were used as a starting point, and data were centered but not otherwise transformed.

### Mapping methods

The map was based on WorldView-2 (WV2) satellite imagery (resolution 0.46 m panchromatic, 1.85 m multispectral) captured in July 2013 and July 2014. The 16.4-km wide images were orthorectified and clipped to the study area in ArcMap 10.6 [47]. The multi-spectral imagery was then segmented via the ArcMap ‘Segment Mean Shift’ tool with the following parameters: spectral detail 20, spatial detail 18, minimum segment size 54 pixels, which mimics the size of our 8-m radius ground based plots (200 m^2^) [48]. The spatial and spectral detail parameters used by the proprietary ArcGIS software range from 1 to 20; they are not equivalent to the bandwidth parameter *h* used in the original formulation of the mean-shift method [49], and no additional information on their properties is provided. Lacking an a priori basis for choosing the spatial and spectral parameters, we chose values by trial and error to produce segments distinctly finer than our intended final map. This ensured that all potential objects of interest were differentiated. The unnecessary complexity in the segmentation was then reduced by the classification process, which merged adjacent segments assigned to the same class. The unnecessary complexity in the segmentation was then reduced by the classification process, which merged adjacent segments assigned to the same class.

**Fig 3:**
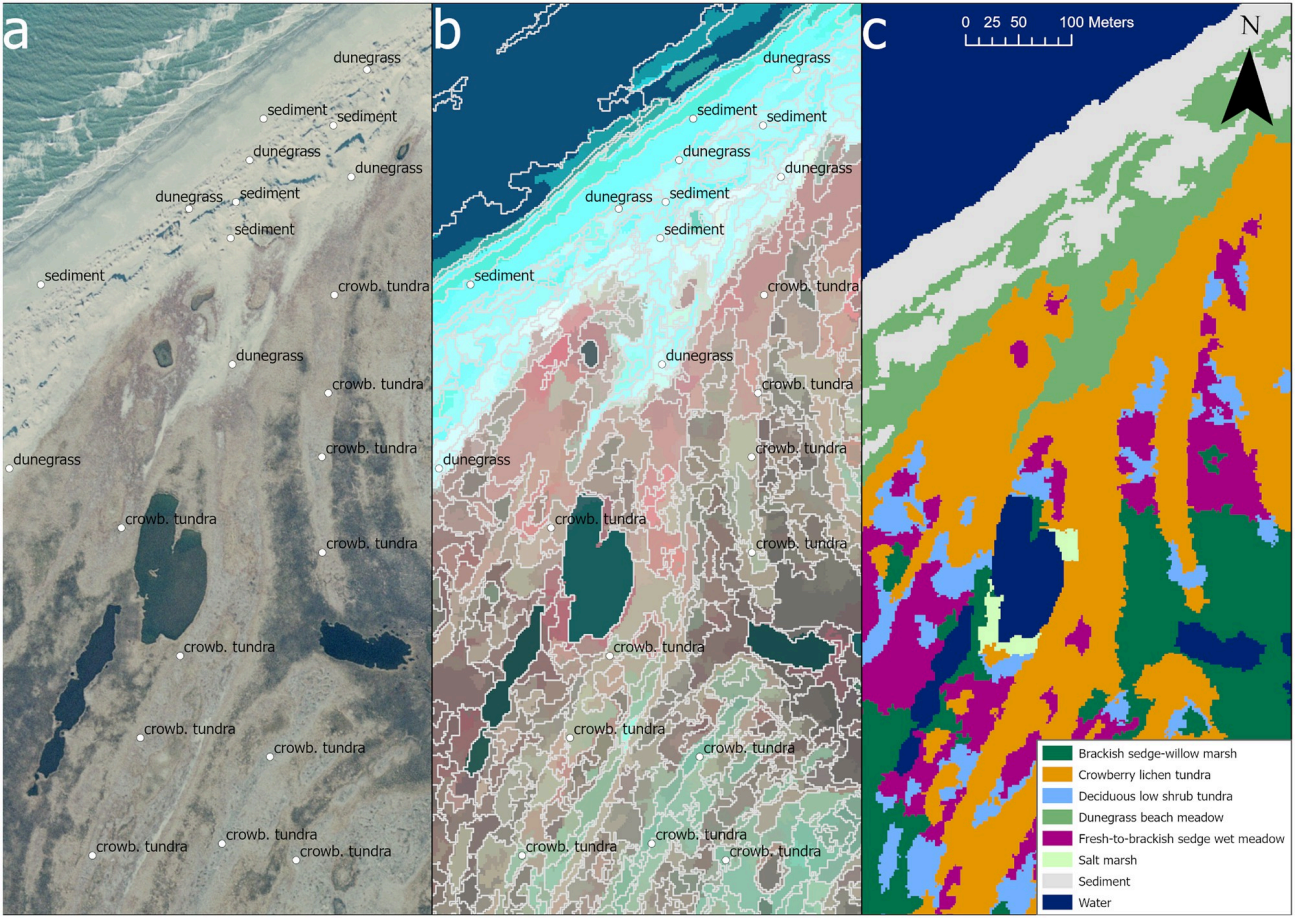
Training points were (a) digitized via interpretation of the imagery, (b) intersected with the segmentation with spatial, spectral and topographic variables extracted, and (c) classified to land cover class via Random Forest. Imagery: Manley, WF, Sanzone, DM, Lestak, LR, and Parrish, EG 2007. Index Layers for High-Resolution Orthorectified Imagery from 2003 for the Coastal Areas of Bering Land Bridge NP (BELA). https://irma.nps.gov/DataStore/Reference/Profile/1044493 Public domain.

After all plots were assigned to classes, the spectral, topographic and spatial variables of image segments containing vegetation plots were used as training data. Further training data were added whenever landcover class was clearly identifiable from the imagery, in order to increase the overall number of training points and to increase representation of rare classes. Sources of imagery for visual interpretation of landcover class included: 1) an orthomosaic of true-color aerial 1:24,000-scale photographs, taken in 2003 by AeroMetric Inc and digitized at 0.6 m resolution of the CAKR and BELA coasts; 2) the statewide 2012 SPOT imagery mosaic; 3) pan-sharpened WV2 imagery (available for the coasts of both CAKR and BELA); and 4) oblique aerial photographs of the coast available through ShoreZone [33]. The land cover class was identified visually at numerous locations using these image sources, and digitized as point locations. Individual points can be digitized much more rapidly than drawing polygons, which are typically used for supervised classifications, at a rate of ten or more per minute (Fig 3). Allowing the segmentation to define training area boundaries reduces much of the decision-making needed during training data collection, allowing for many more points to be collected. This method allows collection of training data which encompasses the full range of variation of each land cover type. A total of 11,647 visually interpreted points with class labels were inferred for BELA, and 6575 from CAKR. The labeled points were then intersected with the segmentation, the variables of the segments where each point fell were extracted, and these records formed the basis of the supervised classification.

A total of 40–46 variables were collected for each segment (Table 1), derived from the WV2 satellite imagery, Alaska 5-meter IFSAR digital terrain model, the National Hydrography Dataset [50] and the 2003 coastal orthomosaic described above. NDVI, the normalized difference vegetation index was calculated from the WV2 images as Near Infrared (NIR)–Red/ (NIR + Red). The normalized difference water index (NDWI) was calculated as Green–NIR / (Green + NIR) [51]. Spectral data was the level 1b radiometrically corrected, 16-bit pixel values, and not further processed to radiance or reflectance.

**Table 1:** Pearson correlation between site variables and the NMS ordination scores.

	r Axis 1	r Axis 2
**Environmental Variables**		
EC (μS/m)	-0.8694	0.4941
Hydric index	1.00^‡^	0.00
Log distance to estuary (km)	0.6974	-0.7167
Log distance to ocean (km)	-0.9998	-0.018
Elevation (m)	0.9892	-0.1467

^‡^Ordination rotated to hydric index.

Only the training data derived from the visually interpreted point locations was used to assign map cover classes to the imagery. We used the plot data and locations to calibrate our visual interpretation of training points, and as a reserved test set for error analysis.

The “unknown” segments (those not identified by visually interpreted training points) were classified by the Random Forest classifier [52]. Random Forest (RF) is a powerful machine learning classifier, widely used in object-oriented mapping due to its high accuracy, and its ability to incorporate high-dimensional model variables and non-normally distributed data [27, 53]. RF is an ensemble classifier, built from many classification-and-regression (CART) trees.

Using the RF decision tree modeling approach as implemented in the R package ‘randomForest’ [54], we classified the segmented map in four ways (Table 2). First, the training data were divided for each satellite scene, and a total of 40 localized RF models were built. Secondly, all the training data were combined to create a single study-area wide model with the scene identifier as a variable. For both modeling approaches, the initial model measure of variable importance was used to reduce the variables to the 15 most important, uncorrelated variables, and the models were then re-run. Prior to the reduction in variables, Spearman rank correlations were used to test the relationship between variables using package ‘Hmisc’ in R [55]. Spearman rank correlations of all 40 variables found three variables that were highly correlated (> 0.9) with other variables at an alpha level of 0.01. These were the segment green band, the mean of the 2003 Orthomosaic green and its standard deviation (S2 Table; p-values S3 Table). Those three variables were removed from consideration for the 15 variables used in the important variable models. For all approaches, we ran the data with 1000 trees and the number variables sampled per node (parameter ‘mtry’) as determined by the function ‘rfTune’.

**Table 2:** Summary of model design.

	All variables	Important variables
	Scene-specific all variable models	Scene-specific important variable models
Scene-specific	***Input***: 40 matrices with 37–45 variable columns and 86–1340 (mean 525) segment rows. ***Output***: 40 RF models, each for a given scene.	***Input***: 40 matrices each with 15 variable columns and 86–1340 (mean 525) segment rows. ***Output***: 40 RF models, each for a given scene.
	Unified all variable model	Unified important variable model
Unified study area	***Input***: 1 matrix with 40 variable columns and 21696 segment rows. ***Output***: 1 RF model for the entire study area.	***Input***: 1 matrix with 15 variable columns and 21696 segment rows. ***Output***: 1 RF model for the entire study area.

A workflow protocol is attached in Appendix 2 in S2 File.

### Error analysis

We used two approaches to assess map accuracy: the bootstrap method provided by RF analysis, and the field plots as an independent test. We computed errors using the field plots as verification data, because the classifications were performed solely with the photo-interpreted points as training data. Our field plots were placed intentionally, and they do not provide an unbiased estimate of the proportion of each type in the study area. However, within each type they provide a useful check on accuracy that can be compared with the OOB error rates.

Because RF is based on classification trees that leave out a random subset of the data, RF can calculate out-of-bag (OOB) error, the percent of the training data left out of the bootstrapped sample that is misclassified. Breiman [52] found OOB error to be an accurate metric that did not necessitate leaving out an independent test set. However, Millard and Richardson [27] found that OOB error was inflated for high-dimensional models relative to an independent test set, and recommend reducing the variable set to uncorrelated, high-importance variables; these formed the basis of our decision to include only the top 15 uncorrelated variables for both modelling approaches. Note that OOB error for individual classes is an estimate of “producer’s accuracy” [56], i.e., the percent of observations (segments in our case) from a known class that were correctly classified.

## Results

### Vegetation classification

The ordination’s final stress was 0.1271, with an instability (standard deviation of change in stress over the previous ten runs) of 0.00015. The observed non-metric fit for the ordination was 0.984.

**Fig 4:**
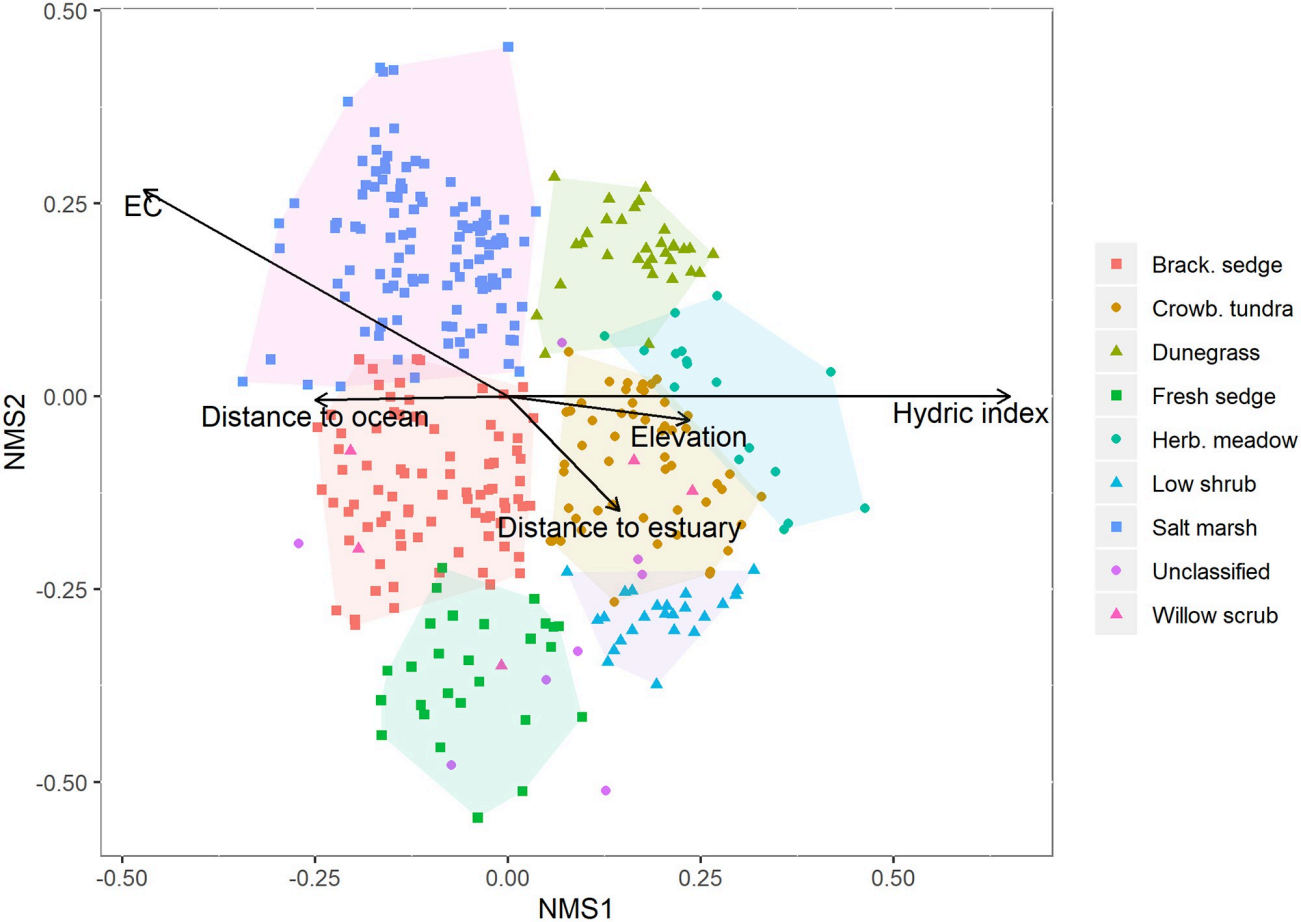
Ordination of plots using NMS. The point cloud was rotated to align the hydric index maximally with Axis 1. The 5 variables most correlated with Axes 1 and 2 are displayed (Table 3). Colored symbols refer to the vegetation classes, defined in Table 2. Classes were grouped by convex hulls, with Willow Scrub and unclassified plots excluded.

The covariates elevation, hydric index, salinity (EC) and the log of distances to ocean or estuary bodies were used as correlation overlays in the ordination, as these were environmental factors most highly correlated with ordination axes and of greatest interpretive value ecologically. There was a strong negative correlation between elevation and EC (-0.76) and a moderate positive correlation between distance to the ocean and EC, but otherwise weak relationships between variables (S1 Table). The ordination was rotated to align Axis 1 maximally with the hydric index, the most strongly correlated factor, r^2^ = 0.42 (Table 3; Fig 4). A higher hydric index indicates a drier site, thus this axis appears to represent a wetness gradient. The structure of the ordination is similar to the ecological sequence seen along beach ridges. Axis 2 separates plots closest to the ocean from those that are most isolated from salt water with transitional, mid-salinity plots in between. EC was not perpendicular to the ordination—presumably because EC registered as 0 in dry soils, regardless of their proximity to the ocean. Surprisingly, neither distance to ocean or distance to estuary was a highly correlated variable. This may be because linear distance from the ocean or an estuary does not accurately represent protection from storm surge. For example, a site 500 m from the ocean protected by two intervening beach ridges is not equivalent to a site 500 m from the ocean along contiguous low-lying salt marsh.

**Table 3:** Segment variables and their imagery sources.

Variable(s), grouped by source	Units	Notes
**Segmented image**		
Pixel count	count	Area approximation
Compactness	index	Range is 0 to 1, where 1 is a circle.
Rectangularity	index	Range is 0 to 1, where 1 is a rectangle.
Longitude of segment centroid	m	Alaska Albers projection
Latitude of segment centroid	m	Alaska Albers projection
Distance from centroid to ocean	m	Calculated from National Hydrography Dataset ocean shapefile
Segment color, NIR	nm	
Segment color, green	nm	
Segment color, red	nm	
**WV2 satellite image**		
Red, mean & standard deviation	nm	
Green, mean & standard deviation	nm	
Blue, mean & standard deviation	nm	
NIR2, mean & standard deviation	nm	
Coastal, mean & standard deviation^1^	nm	
Yellow, mean & standard deviation^1^	nm	
NIR1, mean & standard deviation^1^	nm	
RedEdge, mean & standard deviation^1^	nm	
NDVI	index	NIR–Red/ (NIR + Red) Range -0.73 to 0.80
NDWI	index	Green–NIR / (Green + NIR) Range: -0.59 to 0.75
Satellite scene unique identifier^2^	categorical	
Date of satellite image^2^	categorical	
**IFSAR 5-m Digital Terrain Model**		
Elevation, mean & standard deviation	m	
Curvature, mean & standard deviation	index	Range 7 to -7, where 0 is a straight surface, positive values are concave and negative convex.
Aspect, northness and eastness	index	Calculated as cos(aspect × π)/180 and sin(aspect × π) /180. Range -1 to 1, where -1 = south, 1 = north. Likewise, for eastness, -1 = west, and 1 = east.
Slope, mean & standard deviation	degrees	
**IFSAR 5-m Digital Terrain Model & IFSAR 5-m Digital Surface Model**		
DSM height above DTM, mean & standard deviation	m	vegetation height proxy
**2003 Coastal Orthomosaic**		
Red, mean & standard deviation	nm	
Green, mean & standard deviation	nm	
Blue, mean & standard deviation	nm	
**Location**		
Park (BELA/CAKR)^2^	categorical	

^1^ Collected for 3 satellite scenes which had all 8 WV2 bands.

^2^ Used in the unified models.

Classes were further separated subjectively using the ordination. Several highly diverse, forb-dominated plots in CAKR clustered consistently from crowberry lichen tundra, in the now designated ’Herbaceous meadow’ vegetation type (see Appendix 1 in S1 File). The brackish plots intermediary to salt marsh and freshwater sedge were dominated by *Carex rariflora* and *Salix ovalifolia*, and assigned to the ‘Brackish sedge wet meadow’ type.

Using the ordination and sorted table analyses, we describe 12 vegetation types as formal NCVS plant associations (Appendix 1 in S1 File). The map includes these 12 vegetation types, as well as six other broad classes, which we will hereafter collectively refer to as land cover classes (Table 4). The six broad classes include the non-vegetated classes of water, sediment and built-up (human infrastructure), Salt Marsh-Water Mosaic, and two non-coastal vegetated classes, Tall Shrub Upland and Upland Tundra. Salt marsh-water mosaic is identical in terms of plant cover to salt marsh, but includes a high percent cover of water, and is spectrally distinct. The two non-coastal classes, Tall Shrub Upland (dense vegetation found along steep slopes and freshwater riverine systems) and Upland Tundra (consisting of low shrub-sedge tundra outside of tidal influence), are mapped to delimit the study area. These two classes were only incidentally sampled and we do not include them in the description of vegetation types. Full descriptions of the vegetation types, as well as a dichotomous key and data summaries of plots are included in Vegetation Types, Appendix 1 in S1 File.

**Table 4:** Map land cover classes.

Land cover class (Abbreviated name)	Characteristic plants	No. field plots	General notes
Brackish Marestail Marsh (Marestail)*	*Hippuris tetraphylla*	10	Monoculture of emergent vegetation, found in shallow, brackish ponds.
Brackish Sedge-Willow Marsh (Brack. sedge)	*Carex rariflora*, *Salix ovalifolia*	100	At higher elevations or greater distances from coastal water than Salt marsh, slightly drier and less saline.
Built-up		-	Human infrastructure.
Crowberry Lichen Tundra (Crowb. tundra)	*Empetrum hermaphroditum*, *Thamnolia vermicularis*, *Leymus mollis*	46	Dry beach ridges found further from the coast than dunegrass beach meadow. Older beach ridges develop higher lichen cover.
Deciduous Low Shrub Tundra (Shrub tundra)	*Betula nana*, *Ledum palustre*	16	Vegetation typical of non-coastal arctic shrub tundra; found in the study area in innermost beach ridges of larger complexes. Transitional from crowb. tundra.
Dunegrass Beach Meadow (Dunegrass)	*Leymus mollis*, *Lathyrus maritimus*	25	Immediately adjacent to the beachfront, well-drained soils. Low diversity, early successional type.
Freshwater sedge wet meadow (Wet sedge)	*Carex aquatilis*, *Eriophorum angustifolium*	26	Vegetation typical of wet arctic tundra; found in most protected swales of large beach ridge complexes.
Grayleaf Willow Scrub (Willow scrub)	*Salix glauca*	5	Low (< 1 m) willow thickets found uncommonly near the outer beach ridge. CAKR only.
Herbaceous Dry Beach Ridge Meadow (Herb. meadow)	*Artemisia tilesii*, *Epilobium latifolium*, *Saxifraga tricuspidata*	17	Similar to crowb. tundra, but with high diversity and cover of forbs. CAKR only.
Lyngbye’s Sedge Meadow* (Lyng. sedge)	*Carex lyngbyei*	11	Monoculture of halophytic sedge found in standing brackish water, typically swales immediately adjacent to the outermost beach ridges.
Pendantgrass Lagoon Margin* (Lagoon margin)	*Arctophila fulva*	22	Heterogenous, narrow (~2–8 m) bands of emergent vegetation found along inner lagoon edges. BELA only.
Rock Sedge Marsh* (Rock sedge)	*Carex saxatilis*, *Iris setosa*	3	Narrow, brackish swales. Found only on Cape Krusenstern beach ridge complex.
Salt Marsh	*Carex subspathacea*, *Puccinellia phryganodes*	162	Low-lying vegetation on soils saturated with salt water. Low diversity veg. type that is highly important migratory bird forage [57].
Salt Marsh Mosaic (Salt marsh mos.)	*Carex subspathacea*, *Puccinellia phryganodes*	-	Salt marsh vegetation pocketed with small water/mud patches.
Sediment		11	Non-vegetated, non-water surfaces including sand, rock, gravel and mud.
Tall Shrub Upland (Shrub upl.)	Not included in vegetation type descriptions.	9	Shrubby bluff often present between coastal vegetation and upland.
Upland Tundra (Upland)	Not included in vegetation type descriptions.	5	Shrub and sedge tundra beyond tidal influence.
Water		-	Coastal and fresh water bodies.

Map classes, with dominant plant species and a description.

*Predominately monoculture class not included in ordination.

Attempts to separate the large Brackish Sedge-Willow Marsh cluster of plots into willow- or sedge-dominated classes were not consistent in the ordinations. The spectrally distinct ‘salt marsh-water mosaic’ ecotype was added to the map to display the estuary and lagoon islands composed of a fine patchwork of mud/water pockets between salt marsh vegetation. In terms of species composition, these were identical to salt marsh plots. One undersampled type that did not ordinate consistently, Grayleaf Willow Shrub, was included as a type because it was very distinct on the imagery (see S1 File).

Several potential types were so rare on the landscape that we were unable to include them and designated them as ‘unclassified’. These include two *Sphagnum* (peatmoss) dominated plots on the edge of lakes in Cape Krusenstern, CAKR, and *Juncus arcticus* (Arctic rush) dominated swales in sparsely vegetated beach ridge swales in BELA. Also included are three plots with the freshwater aquatic emergent species *Hippuris vulgaris* (common marestail). Due to the difficulty in mapping halophytic *Hippuris tetraphylla* (fourleaf marestail), which is more prevalent in the study area but still generally rare, we also designated these plots as unclassified. Ultimately, 5 plots were designated as outliers, 12 as unclassified, and 8 had low enough vegetation cover (<10%) that they were mapped as sediment.

### Variable importance

Variable importance is measured two ways for random forest trees: mean decrease accuracy (MDA), how much accuracy decreases when a variable is excluded from the model, and mean decrease Gini (MDG), a measure of how homogenous nodes including the variable are. We tested the stability of both measures with 100 random forest models using jackknife runs leaving out 10% of the data (ala [58, 59]) and found them both to be highly stable (r^2^ between the rank of each variable from full dataset and the 100 resamples was >.99 for both MDG and MDA, see S4 Table). We report MDA for ease of interpretation, but MDG results are substantively similar (S4 Table).

**Fig 5:**
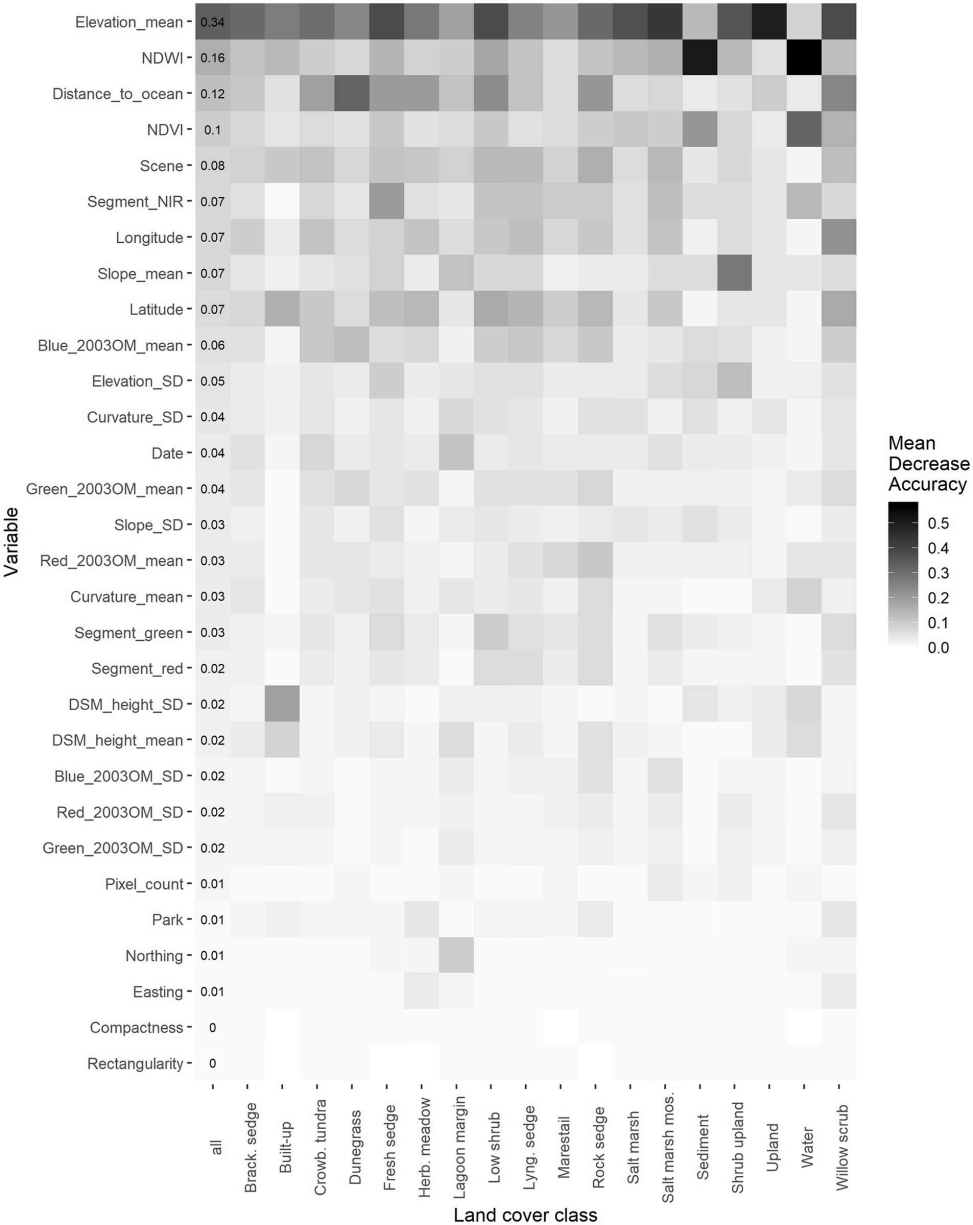
Mean Decrease in Accuracy (MDA) for the unified study area model, by land cover class. The first, labeled column displays the MDA for the model overall.

Fig 5 displays the ranking of the importance of the segment variables for land cover classes, as measured by MDA for the unified, all-variable model. Elevation is the single most important variable, especially for Upland Tundra and the two Salt Marsh classes. Distance to ocean is important generally, and particularly for distinguishing Dunegrass Beach Meadow, the vegetation type found directly at the beachfront. NDWI (a metric of wetness) and NDVI (a metric of vegetation density) are most important for recognizing the unvegetated classes of sediment and water. Latitude and longitude are important variables for most types; many of the vegetation types are structured sequentially along beach ridges. The least important factors are segment compactness and rectangularity, two metrics of segment shape.

### Model comparisons

We compared 4 models, scene-specific models with all variables (Scene spef., all var.) and top 15 important variables (Scene spef., top var.), and a study-area wide unified model with all variables (Unified, all var.) and top 15 important variables (Unified, top var.). We found all 4 models to have generally similar accuracies, with the unified important variable model and scene-specific all variable model in particular having slightly higher overall accuracies, and different strengths in recognizing classes (Table 5).

**Table 5:** The producer’s error for the independent test set of excluded field plots for all four sets of models and out-of-bag producer’s error from each RF model.

		Field plot producer’s accuracy						RF OOB producer’s accuracy			
Class	*n* plots	Scene spef., all var.	Scene spef., top var.	Unified, all var.	Unified, top var.	Class	*n* data	Scene spef., all var.	Scene spef., top var.	Unified, all var.	Unified, top var.
Marestail	10	0.70	0.70	**0.80**	**0.80**	Marestail	389	**0.68**	**0.68**	0.58	0.60
Brack. sedge	100	0.82	**0.87**	0.84	0.85	Brack. sedge	2480	0.83	0.83	0.81	**0.84**
Crowb. tundra	46	0.78	0.75	**0.76**	0.73	Crowb. tundra	1357	0.82	0.84	0.84	**0.85**
Dunegrass	25	0.89	0.89	**0.93**	**0.93**	Dunegrass	1996	0.90	0.90	0.90	**0.91**
Low shrub	16	0.75	0.75	0.75	0.75	Low shrub	722	0.85	**0.87**	0.83	0.85
Wet sedge	26	**0.68**	0.64	0.61	0.61	Fresh sedge	830	0.83	**0.86**	0.85	**0.86**
Willow scrub	5	0.60	0.60	0.60	0.60	Willow scrub	316	0.89	0.88	0.92	**0.93**
Herb. meadow	17	**1.00**	0.94	**1.00**	0.88	Herb. meadow	455	**0.83**	**0.83**	0.74	0.78
Sedge swale	11	0.91	0.91	0.91	**1.00**	Lyng. sedge	135	0.72	**0.81**	0.70	0.72
Lagoon margin	22	0.82	**0.86**	0.82	0.82	Lagoon margin	125	**0.85**	0.82	0.73	0.72
Rock sedge	3	1.00	1.00	1.00	1.00	Rock sedge	22	0.73	**0.77**	**0.77**	**0.77**
Sediment	11	0.50	0.75	**1.00**	**1.00**	Sediment	1411	0.91	0.91	**0.92**	**0.92**
Salt marsh	162	0.77	0.80	**0.81**	0.78	Salt marsh	3657	0.90	0.90	**0.91**	**0.91**
Shrub upland	9	0.70	0.70	**0.80**	**0.80**	Shrub upland	1823	**0.89**	**0.89**	0.88	0.88
Upland	5	0.82	**0.87**	0.84	0.85	Upland	3131	0.92	**0.93**	**0.93**	**0.93**
**overall**	468	0.79	0.81	0.82	0.80	Built-up*	24	**0.88**	**0.88**	0.71	0.67
						Water*	993	0.92	0.92	**0.93**	**0.93**
						Salt marsh mos.*	1830	**0.88**	0.86	0.87	0.85
						**overall**	21696	0.877	0.879	0.877	**0.881**

The number of test plots is higher than the number collected, as some plots occur on overlapping satellite scenes, which were classified separately in all modeling approaches. Such plots were counted as testing each classified scene. In bold: the highest accuracy for a given type. Four-way ties are unbolded. Starred classes in the OOB section indicate land cover classes without field plot test data.

For the independent field test set, accuracy is similar across models (Table 4). Accuracy was 0.7 or better for all types and models except GWS, which was not adequately sampled (n = 5). Several widespread types had accuracies of 0.9 or better. Extremely low (0.5) and high (1) values were obtained only for types with very small sample sizes of 5 or less. Accuracy varies more between classes than it does between models.

**Fig 6:**
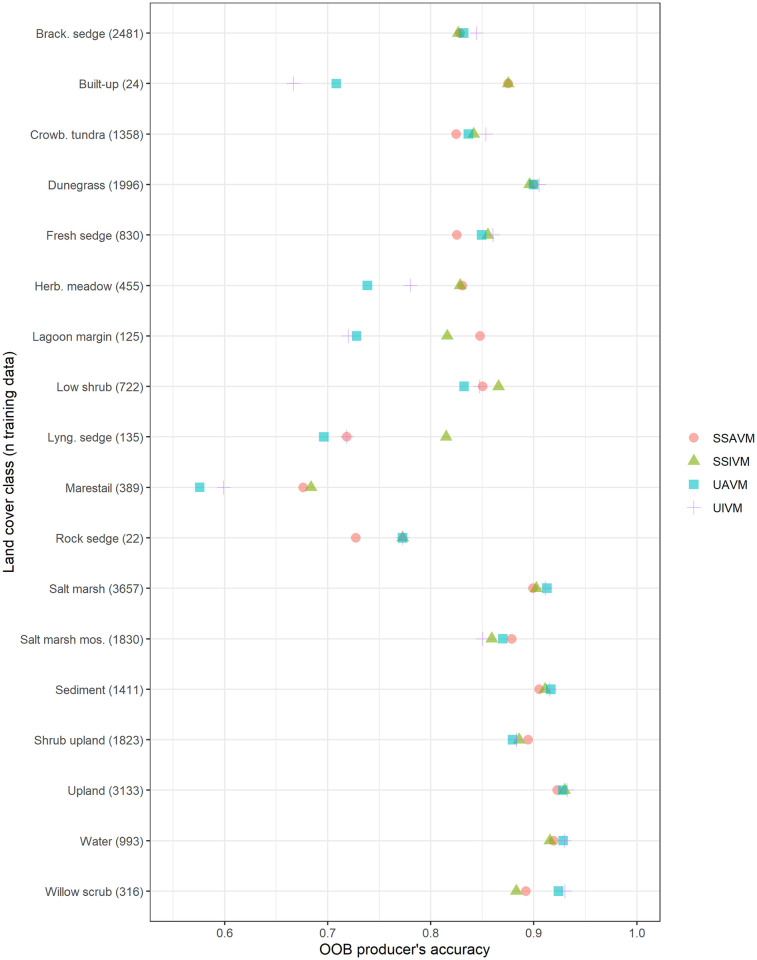
OOB producer’s error for the four modeling approaches, subdivided by land cover class. Land cover classes are ordered alphabetically. The n of training data is presented in parentheses.

**Fig 7:**
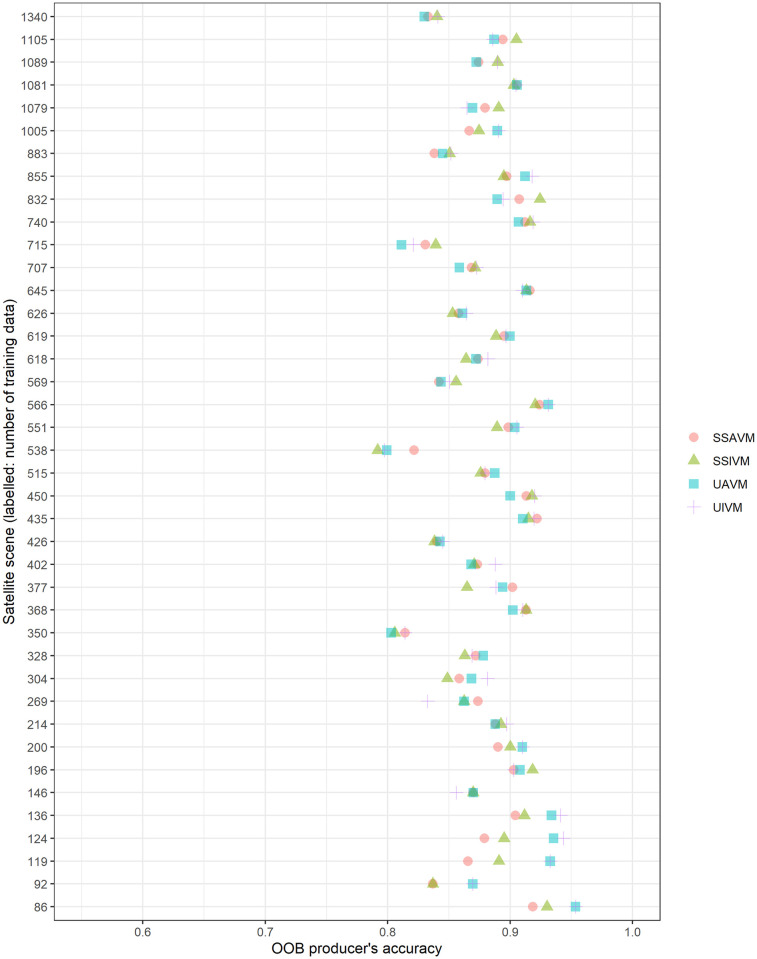
OOB producer’s error for the four modeling approaches, subdivided by satellite scene. Scenes are ordered by amount of training data.

For OOB error, most classes have very similar accuracies across models (Fig 6; Table 4). Some of the rarer classes have a wider range in accuracy, with the unified models being notably worse for these classes. When out-of-bag errors are parsed by satellite scene (Fig 7), again, error rates between scenes vary more than between models, that is to say, some scenes consistently classify better than others, regardless of model used. There is no consistent relationship between training data sample size and accuracy in by-scene comparisons (data S5 Table, multiple linear regression results S6 Table, adjusted R^2^ = 0.008).

Reducing the model to the fifteen most important variables increased accuracy only for the unified model. This can be seen slightly in the field test set results and more strongly for OOB error. For the field test set, the unified, top-variable model was more accurate than its counterpart all variable model in 7 out of 15 classes and tied in 5. For OOB error, the unified important-variable model is more accurate than the all-variable model for 12 out of 18 classes, with a 1.7% increase in overall accuracy (Table 4; Fig 6). In contrast, the scene-specific model sets the unified and important-variable models tie for 11 out of 15 classes of the field test data, and 8 of the 18 classes for OOB error, and have near-identical overall accuracy (Table 4). This is in contrast to the results from Millard & Richardson [27], who found an independent test set necessary to assess the value of reducing variable dimensionality.

The unified, important variable model was the most accurate for a plurality of land cover classes (9 out of 18), the majority of scenes (23 out of 40), and had the lowest overall OOB producer’s error, 11.6%. There is a strong correlation with sample size—widely sampled classes are more accurate in the unified models, while 5 out of 6 of the rarest classes have lower error in the scene-specific models. Visual comparison of the maps produced by the two best models, the scene-specific all variable model and the unified top variable model, found minor differences. For the purposes of this map, our priorities were distinguishing vegetated from non-vegetated areas, and the boundaries of more abundant classes. We chose the unified important variable model to produce the final map.

Table 6 shows the error matrix of the independent test set against this classification. Many of the errors are predictable—dunegrass beach meadow, an often sparsely vegetated type, is most commonly misclassified as sediment. Salt marsh and salt marsh mosaic, which have identical plant associations, are frequently confused. Brackish sedge-willow meadow is found in close proximity to and has gradual transitions from salt marsh and crowberry-lichen tundra, the two types with which it is most confused. Visual inspection of the map shows that inappropriate types are not being mapped on mismatched landforms (i.e. beach ridge types in estuaries), and much of the error is from difficulty in distinguishing transitions. However, the error is uncertain for many of the smaller land cover classes. As we do not have a sampling design proportionate to the area occupied by each class, we do not calculate user’s accuracy. The OOB error matrix is shown in S7 Table.

**Table 6:** Error matrix for the field test plots.

	Marestail	Brack. sedge	Crowb. tundra	Dunegrass	Low shrub	Fresh sedge	Willow	Herb. meadow	Lyng. sedge	Lagoon margin	Rock sedge	Sediment	Salt marsh	Shrub upland	Upland
Marestail	**7**	0	0	0	0	0	0	0	0	0	0	0	0	0	0
Brack. sedge	1	**87**	7	0	1	5	2	0	0	0	0	0	12	0	0
Crowb. tundra	0	1	**36**	0	2	0	0	0	0	0	0	0	0	0	0
Dunegrass	0	3	1	**24**	0	0	0	1	0	0	0	0	2	0	0
Low shrub	0	0	0	0	**12**	1	0	0	0	0	0	0	0	0	0
Fresh sedge	0	1	0	0	1	**19**	0	0	0	0	0	0	0	2	0
Willow scrub	0	0	0	0	0	0	**3**	0	0	0	0	0	0	0	0
Herb. meadow	0	0	1	1	0	0	0	**16**	0	0	0	0	0	0	0
Lyng. sedge	1	0	0	0	0	0	0	0	**10**	0	0	0	0	0	0
Lagoon margin	0	0	0	0	0	0	0	0	0	**19**	0	0	0	0	0
Rock sedge	0	0	0	0	0	0	0	0	0	0	**3**	0	0	0	0
Sediment	0	0	0	0	0	0	0	0	0	1	0	**11**	5	0	0
Salt marsh	0	8	1	0	0	1	0	0	1	0	0	0	**143**	0	0
Shrub upland	0	0	0	0	0	0	0	0	0	1	0	0	0	**7**	0
Upland	0	0	0	0	0	0	0	0	0	0	0	0	0	0	**5**
Water	1	0	0	0	0	0	0	0	0	1	0	0	0	0	0

Columns present the reference (field) classes, rows present the classified data. Correctly classified plots are presented in bold.

### Final map product

**Fig 8:**
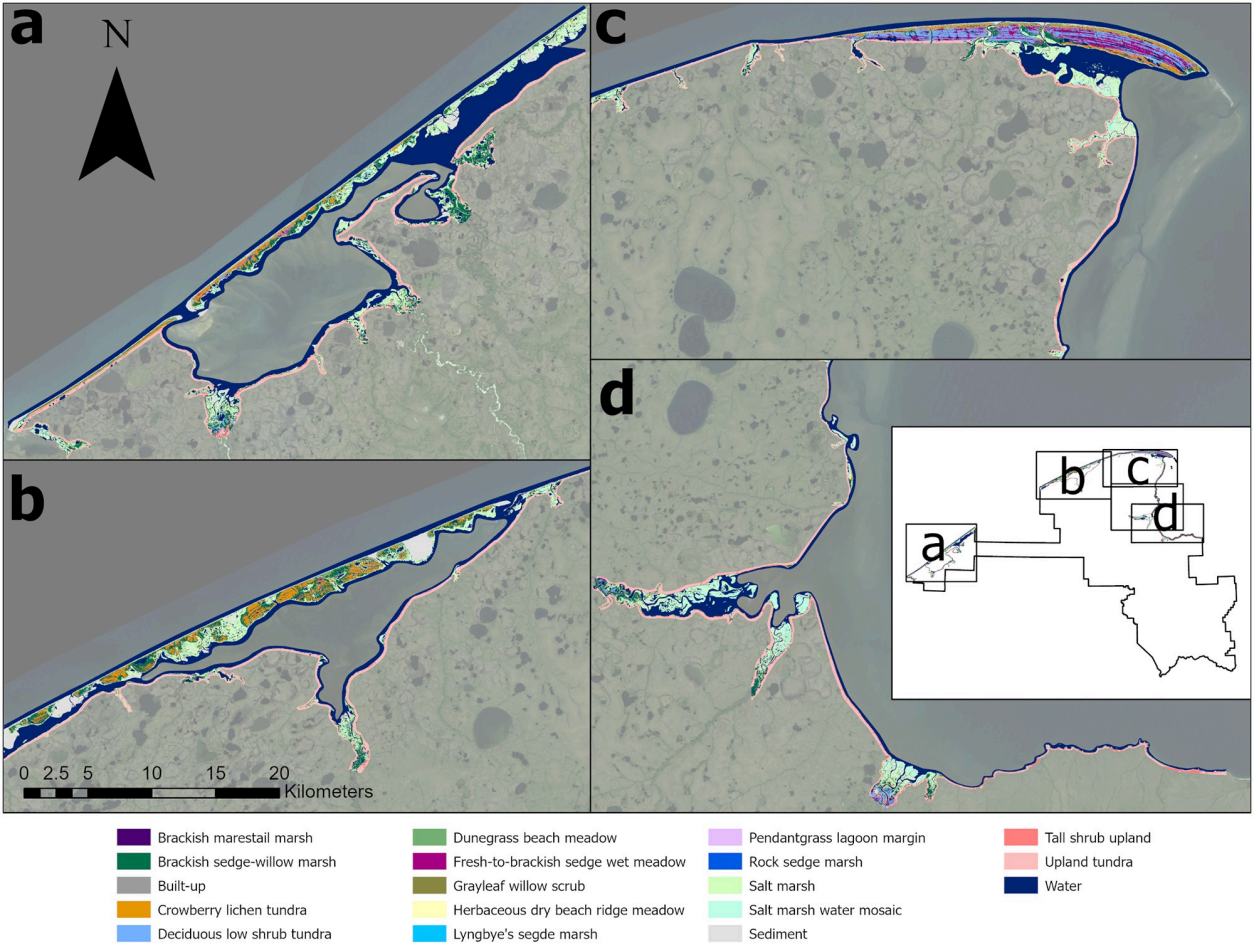
Map of coastal vegetation of Bering Land Bridge National Preserve. Background imagery Landsat mosaic produced for the Arctic Network. https://irma.nps.gov/DataStore/Reference/Profile/2171608 Public domain.

**Fig 9:**
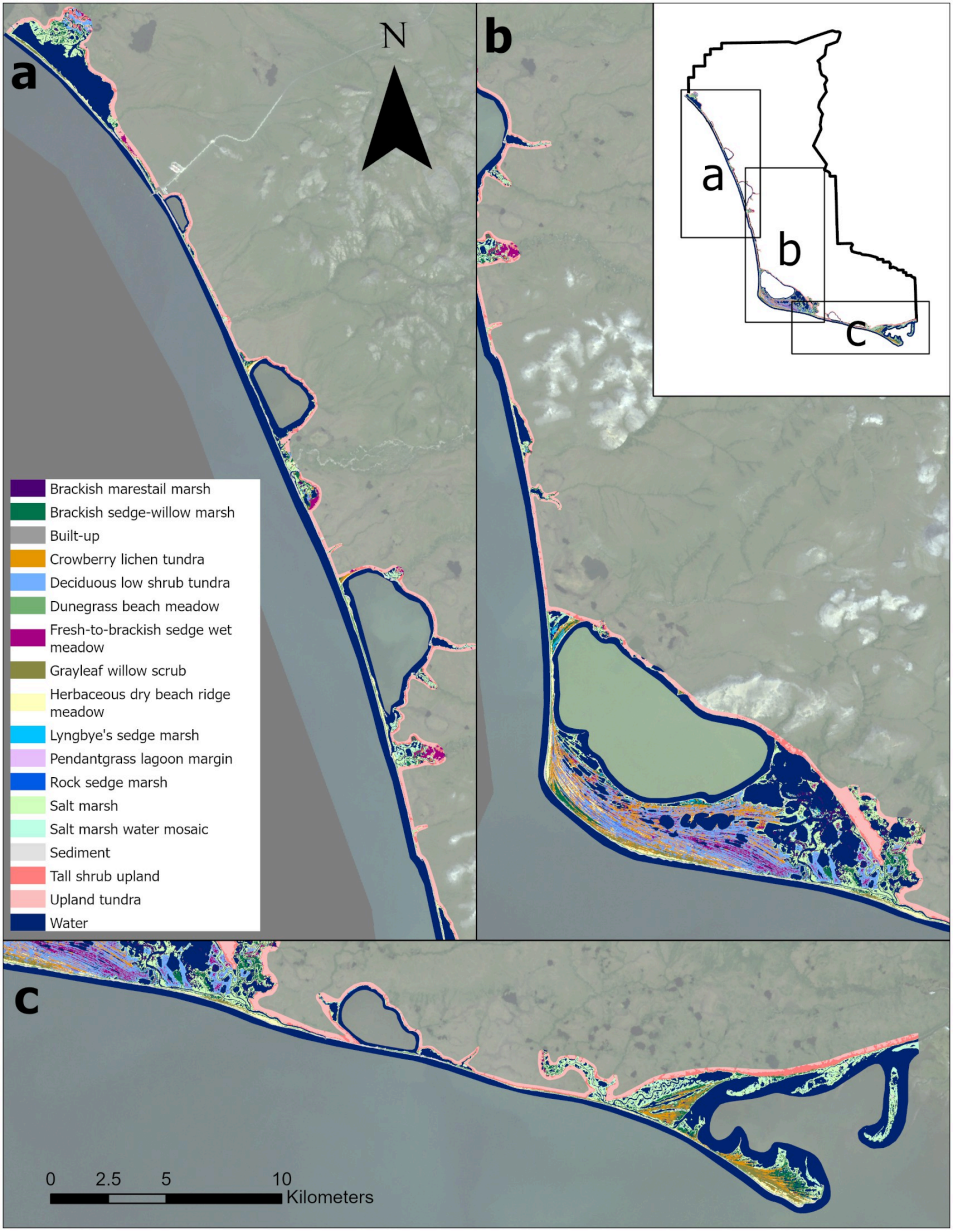
Map of the coastal vegetation of Cape Krusenstern National Monument. Background imagery Landsat mosaic produced for the Arctic Network. https://irma.nps.gov/DataStore/Reference/Profile/2171608 Public domain.

**Fig 10:**
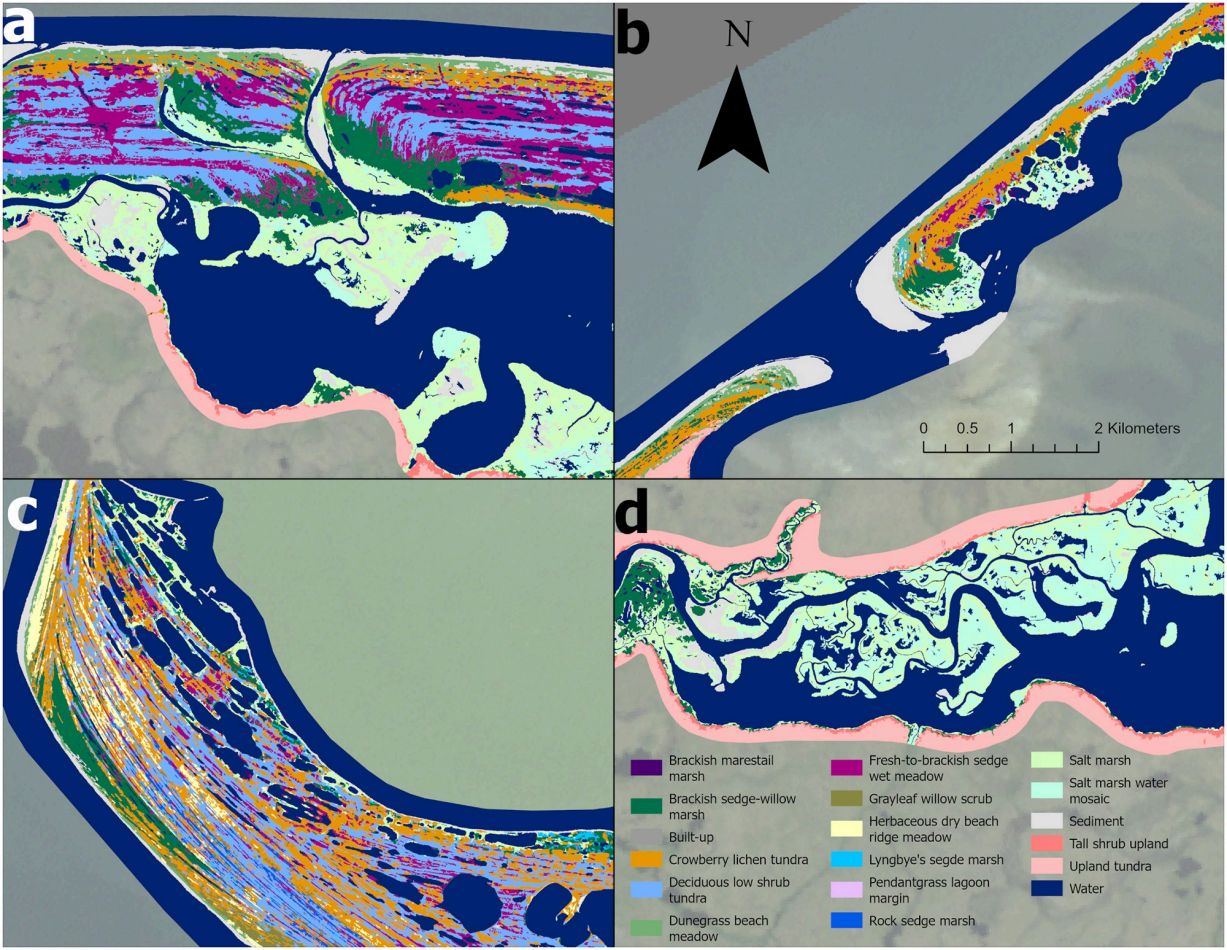
Highlights of the coastal vegetation map. A. Inner beach ridge complex of Cape Espenberg, BELA. B. Outer barrier island of Ikpek Lagoon, BELA. C. Beach ridge complex of Cape Krusenstern, CAKR. D. Nugnugaluktuk estuary, BELA. Background imagery Landsat mosaic produced for the Arctic Network. https://irma.nps.gov/DataStore/Reference/Profile/2171608 Public domain.

The final product is a 663.4 km^2^ map of the vegetation of the BELA & CAKR coasts, available as a vector GIS layer [39]. Figs 8–10 show the maps, as well as close-ups of the detail. The map will also be made available through the Alaska Ocean Observing System’s online Ocean Data Explorer [60]. This data network hosts coastal and oceanographic data from multiple partners, making it readily available to natural resource managers and stakeholders in spill preparation and response.

## Discussion

### Uses of mapping product

We envision three primary uses for these high-resolution coastal land cover layers: spill response, restoration, and long-term ecological monitoring. First, as the Arctic sea ice has retreated, shipping through the Bering Strait has more than doubled since 2008. The multi-agency U.S. Committee on the Marine Transportation System projects likely vessel transit increases of approximately 30% in the next decade [61]. With increased shipping comes an increased risk of spills from the cargo, towing, fishing, and tanker vessels that have constituted 70% of the several hundred vessels in the region each year in the second half of the last decade. Immediately following an oil spill, responders will need high-quality information about the distribution of coastal communities along the shoreline with important conservation value (e.g., salt marshes, brackish sedge-willow marshes) and a high likelihood of oil retention (i.e., areas of low wave energy, marine eddies, etc.) Low-lying, backwater vegetation communities generally have high importance as bird breeding areas [62] and oil releases have the potential to remain on site for decades [33, 63]. Site-specific oil spill response strategies have been established by the State of Alaska for sensitive areas [64], but little is known about the vegetation and habitat composition within these sites. In the event that prioritization of response resources is needed due to logistical issues in a response, our data layers will allow area estimation of the most sensitive habitats, thus enabling data-based triage. Overlaying Shorezone’s Oil Residency Index [33] onto land cover classes of interest could assist in developing a plan for prioritization of response resources.

Second, in the years following a spill, our land cover layers could prove valuable in guiding both restoration and Natural Resource Damage Assessment proceedings [65]. In a spill reaching U.S. lands, the responsible party is required to pay the cost of restoration, and the landowner and other regulatory parties determine the necessary restoration action. If the spill causes damage on NPS lands, NPS requires restoration of vegetation communities and physical site properties to their original, natural condition [66, 67]—in contrast to other landowners that may require only simple revegetation or restoration of ecological function. Our data layers can provide necessary information about composition, aerial extent, and location of affected communities. Using the characteristics of the unaffected areas of a given cover class in Appendix 1 in S1 File (vegetation type descriptions, S1 File) coupled with detailed vegetation work in the affected area and adjacent unaffected areas, restoration staff can create targets against which restoration efforts may be evaluated.

Lastly, these layers may serve as an excellent baseline against which to compare future vegetation changes from climate change and landscape changes that follow increasing coastal erosion [28]. NPS’s Arctic Network has conducted long term monitoring of terrestrial vegetation on NPS Arctic units since 2004 [35]. Because our data layers are high-resolution, they will permit an assessment of coastal landcover change in future decades using similarly high-resolution imagery that is becoming increasingly available from both public and commercial sources.

### Use of mapping method

This is one of the first large-scale, high-resolution object-oriented classifications of any National Park unit, and demonstrates that such methods are functional for conservation efforts. This classification method has several advantages as well as limitations. In per-pixel classifications, isolated pixels can be misclassified in the midst of the true, homogenous type, resulting in ‘salt-and-pepper’ land cover maps [17]. Segmentation reduces this by aggregating pixels into more natural units, and improves the display quality of the final map. Additionally, 30 m^2^ pixels are often mixtures of communities because they straddle boundaries or ecotones. Segments greatly reduce the number of boundary pixels, making more homogenous areas that are easier to classify. Segmentation also allows for object-level variables to be collected, such as shape parameters or texture, though we did not find these useful for our classification of a primarily undeveloped landscape.

As is true of any classification, high quality ground-based data is essential. Our total of 443 plots is substantial and well-distributed, but there are still several kilometer-wide gaps between some samples, due to the remote nature of the Chukchi coast and the existence of parcels of private land along the shore. The ability to recognize vegetation types from satellite imagery or aerial photography is often difficult, and the existence of continuous oblique photography along the coast available through ShoreZone [33] was a major asset to this project.

The method of identifying training points and then intersecting those identifications with the segmentation has yet to be widely used. It is in essence the same as the methods found internally for some object-oriented software workflows, where imagery is first segmented and the training data is collected via manually identifying segments [68]. The point-based method has the advantage that it can be adapted to any segmentation. Placing and categorizing training points is much faster than delineating polygons, allowing for a much larger set of training data to be collected. Collection of training points can be undertaken in a systematic way, via a grid or random sample. We did not use this method because of the large number of types we were attempting to recognize; assigning training points to unambiguous areas is more efficient.

Classifying each satellite scene by a separate model has certain advantages, allowing disparate variable information and potentially better inference for localized classes. For our map, we found minor differences between scene-specific and unified models. Including the scene identifier as a model variable allowed the decision tree forest to incorporate the local differences in classes. All but two of our satellite tiles were WV2 imagery taken within a year of each other, and we were able to collect 40 variables for all scenes, making the data consistent across the study area. Maps with wider variation in data availability and spectral information between scenes may perform better with scene-specific models.

We improved on the spatial resolution of the existing coastal land cover map, going from 900 m^2^ Landsat pixels to minimum 200 m^2^ polygons, and the ecological resolution, increasing from 5 coastal types to 12. Our accuracy rates, at 88.4% OOB producer’s accuracy for the final model, surpassed our target of 80% accuracy.

## Supporting information

S1 Table Spearman correlation of site variables. (CSV)

S2 Table Spearman correlation of RF model variables. (XLSX)

S3 Table P-values for Spearman correlations of RF model variables. (CSV)

S4 Table Stability of variable importance measures. (CSV)

S5 Table N training points and producer’s accuracy for each satellite scene. (CSV)

S6 Table Results of multiple linear regression for each satellite scene producer’s accuracy by *n* data and model. (CSV)

S7 Table RF OOB producer’s accuracy for all 4 models, by satellite scene. (XLSX)

S1 File Vegetation class descriptions, including a dichotomous key. (ZIP)

S2 File Workflow appendix. (DOCX)
