# Supplementary material for: A high-resolution map of coastal vegetation for two Arctic Alaskan parklands: An object-oriented approach with point training data
Source: PLoS One. 2022 Aug 31;17(8):e0273893. doi: 10.1371/journal.pone.0273893 (PMC9432696; doi:10.1371/journal.pone.0273893)
Supplement: S1 File — (ZIP) [file pone.0273893.s008.zip › Appendix1_Vegetation_types/Appendix_1_Vegetation_types.docx]

# Appendix 1: Coastal Vegetation Types for Bering Land Bridge Preserve and Cape Krusenstern National Monument

Supplemental information to Hampton-Miller, Neitlich & Swanson 2020.

# Introduction

We present 12 coastal vegetation types, listed alphabetically by newly proposed common name. We include their position within the U.S. National Vegetation Classification (USNVC) hierarchy at two levels.

The USNVC has an 8-level hierarchy. The upper three levels are classified by structure (e.g. trees vs shrubs), the middle levels by biogeography and the lower two by floristics (the particular species present). We categorize our vegetation types by two of the USNVC levels: Groups, level 6, and Associations, level 8. The Group, the lowest of the middle levels, is defined by “combinations of relatively narrow sets of diagnostic plant species, including dominants and co-dominants, broadly similar composition, and diagnostic growth forms” (USNVC 2019). Groups are the lowest level currently available for the USNVC, and many have yet to be classified for Alaskan vegetation. Plant associations, the lowest level, are more narrowly defined by diagnostic species. Many plant associations have already been described for Northwest Alaska, and we cite existing associations when possible. Most of our mapped vegetation types include several closely associated plant associations, found in similar habitats, such that they are distinguishable on aerial imagery (see Mapping Methods).

We also compare all types to two other classifications systems widely used within Alaska, the National Park Service Arctic Network (ARCN) landcover ecotypes [1] and the Alaska Vegetation Classification [2]. We compare our vegetation types to Jorgenson et al.’s [1] ecotypes (which integrate physiography, vegetation and surface form), as those are the units described and mapped in their report.

Two tables are presented for each type. The first, a summary of the median and inner quartile range (IQR) of each site variable collected (see Methods). The second is a contingency table of the cover class for the species present. Species that occurred at or above 10% frequency are included, which excludes most plants that occur only once. Plant names follow Parker [3]. We publish type names verbatim, with synonymy noted where applicable.

# Key to Coastal Vegetation Types for Bering Land Bridge Preserve and Cape Krusenstern National Monument

This key is a based on identifying vegetation in homogenous stands, approximately 200 m^2^ in area. To identify vegetation using this key, identify all species above 1 percent cover to one of the following five cover categories: 0:<1%, 1:1-5%, 2:6-25%, 3:26-50%, 4:51-75%, 5:75-100%. Visually estimate the cover of the following functional groups: shrubs (woody plants), graminoids (grasses, sedges & rushes), forbs (herbaceous non-graminoid vascular plants), mosses and lichens.

This key is meant to serve as a general guide. Check any type identification against the listed species in the type descriptions. Vegetation types are necessarily transitional, and types were assigned to frequently co-occurring species groups.

We use the term dominant to indicate a species that has the highest cover of any present.

1. Less than 10% live plant cover ………………….…………………………………………………….………………………….………. 2

1. 10% or more live plant cover ...……………….…….………………………………….………………….…………………….………. 3

2. Majority of cover comprised of sand, mud or gravel …..…………………….…………………….……………. **Sediment**

2. Majority of cover is standing water ………………….………………….………………….………………………………… **Water**

3. Not part of a beach ridge or estuarine complex, soils not brackish or saline (EC <200 μS/m), elevation usually 10 m above sea level or higher……………………………………………………………………………….………….……… 4

3. Part of a beach ridge or estuary complex, soils saline, brackish or fresh, elevation below 10 m (some beach ridges excepted) ………………………………………….……………………………………………………………………………… 5

4. Closed shrub canopy, found on steep slopes or at the transition between estuarine and riverine systems .………………………………………………………………………………………………………………………..…………… **Tall shrub upland**

4. Sedge or open low shrub tundra, often with polygonal ice wedges, wet or mesic, not found on steep bluffs or river bars …………………………………………………………………………………………….……………… **Upland tundra**

5. Cover of shrubs is > 25%, or the highest cover of any functional group .…………………………………………….. 6

5. Shrubs < 25% cover, graminoids or forbs predominant ………………………………….………….…………………….. 10

6. *Salix glauca* the highest cover of any species [dominant] ..……..……...…………………. **Grayleaf willow scrub**

6. Other shrubs dominant ………………………………………………………………………………………………………………….…. 7

7. Creeping willow, *Salix ovalifolia* or *Salix fuscescens*, dominant, soils brackish ………………………………….. ………………………………………………………………………………………………….………………. **Brackish sedge-willow marsh**

7. Ericaceous shrubs or dwarf birch dominant, soil well drained or not brackish ……………………….…….…….. 8

8. *Empetrum hermaphroditum* (crowberry) dominant ………………………………………………….………………..……… 9

8. *Betula nana* dominant, or co-dominant with *E. hermaphroditum* and ericaceous shrubs ……………………………………………………………………………………………………………………. **Deciduous low shrub tundra**

9. Low vascular plant diversity (fewer than 5 species) ……………………………..……….. **Crowberry lichen tundra**

9. High vascular plant diversity (5 or more species) ………………………… **Herbaceous dry beach ridge meadow**

10 (5). Majority of live plant cover graminoids ………………………………………………………….…….……………..…… 11

10. Majority of live plant cover forbs (including *Hippuris*) ……………………..…………………………………………….. 17

11. *Carex lyngbyei* or *C. saxatilis* dominant …………………………………..………………………………………………….…… 12

11. Other graminoid species dominant ……………………………………..……….…………………………………………….….. 13

12. *Carex lyngbyei* dominant, occurring in brackish swales or lagoon margins ………………………………………… ………………………………………………………………………………………………………….………….…… **Lyngbye’s sedge marsh**

12. *Carex saxatilis* dominant ………………………………………………….…………….………………………. **Rock sedge marsh**

13. *Leymus mollis* or *Poa eminens* dominant, low species diversity, well-drained, sandy or gravelly soils, close to ocean or lagoon, often sparsely vegetated ……………………………………………………………………………………..………. **Dunegrass beach meadow**

13. Soils not well drained, sedges or other grasses dominant ……………………………………………………………... 14

14. Saline soils, low elevation, dominated by low mats of *Carex subspathacea* or *Puccinellia phryganodes ………………………………………..*………………………………………………..…………………………………….………….….. **Salt marsh**

15. Brackish to fresh soils, other sedges dominant ………………..……………………………………………….…………….. 16

16. Brackish soils, without standing water, dominant species *Carex rariflora*, may be co-dominant with dwarf willows *Salix ovalifolia* or *S. fuscescens* …………………..………………..…….. **Brackish sedge willow marsh**

16. Fresh to brackish soils, often with standing water, *Carex aquatilis* and/or *Eriophorum angustifolium* dominant ……………………………………………………………………………………… **Fresh to brackish wet sedge meadow**

17 (10). Emergent vegetation from saline or brackish pools or lagoons, *Hippuris tetraphylla* dominant ………………..….………………………………………………………………..…………………………………. **Brackish marestail marsh**

17. Terrestrial vegetation …………………………………………………………………………….………………………………………. 18

18. Saturated, saline soils, dominated by *Potentilla egedii¸ Chrysanthemum arcticum* and/or *Stellaria humifusa*. Graminoids *Carex subspathacea* or *Puccinellia phryganodes* may also be present ………………. ………………………………………………………………………………………………………………………………….…………..… **Salt marsh**

18. Well-drained soils, high forb diversity (5 or more species), such as *Epilobium latifolium*, *Artemisia tilesii*, *Saxifraga tricuspidata* or other coastal Apiaceae species ……… **Herbaceous dry beach ridge meadow**

# Vegetation type descriptions

## Brackish marestail marsh (BMM)

Figure 1. Brackish Marestail Marsh, plot A-20180716-17, eastern Krusenstern Lagoon, CAKR. NPS photo, public domain.

**USNVC Group:** Not classified.

**Plant association:** *Hippuris tetraphylla* [1,4]

**Alaska Vegetation Class:** III.D.2.a. Four-Leaf Marestail

**ARCN Landcover Ecotype:** Coastal Aquatic Brackish Marestail Marsh (not mapped)

Found where brackish to saline ponds are shallow enough to allow emergent vegetation. Although the plots in the table do include other species, this is an artifact of plot placement on pond margins. Most 200 m^2^ stands of *Hippuris tetraphylla* are in ponds and inaccessible. We attempt to map pure stands of *Hippuris tetraphylla* in the coastal classification. This type has one of the highest error rates of all types included (see Results) as it is often indistinguishable from water and occurs in small patches.

*Hippuris tetraphylla* is the halophytic species found in Alaska, opposed to the more common, freshwater *Hippuris vulgaris*.

Table 1. Site variable summary for Brackish Marestail Marsh (n=10).

|  | **Median** | **IQR** |
| --- | --- | --- |
| EC (µS/m) | 4000 | 0 |
| Hydric index | 1 | 0 |
| Elevation (m) | 4.5 | 2.3 |
| Distance to ocean (km) | 1.4 | 2.6 |
| Distance to estuary (km) | 0.2 | 0.3 |
| *Functional group cover (%)* | | |
| Graminoid | 0 | 5 |
| Forb | 32 | 22.5 |
| Dwarf shrub | 0 | 0 |
| Low shrub | 0 | 0 |
| Tall shrub | 0 | 0 |
| Lichen | 0 | 0 |
| Moss | 0 | 0 |

Table 2. Species cover contingency table for Brackish Marestail Marsh plots (n=10).

|  | Cover Class Frequency (%) | | | | | |  |
| --- | --- | --- | --- | --- | --- | --- | --- |
|  | 0:<1% | 1:1-5% | 2:6-25% | 3:26-50% | 4:51-75% | 5:76-100% | **Total Frequency (%)** |
| *Hippuris tetraphylla* | 10 | 10 | 20 | 20 | 30 | 10 | 100 |
| *Carex subspathacea* | 20 | 20 | 0 | 0 | 0 | 0 | 40 |
| *Caltha palustris* | 20 | 0 | 0 | 0 | 0 | 0 | 20 |
| *Carex glareosa* | 0 | 20 | 0 | 0 | 0 | 0 | 20 |
| *Sparganium* sp*.* | 0 | 20 | 0 | 0 | 0 | 0 | 20 |
| *Carex aquatilis* | 10 | 0 | 0 | 0 | 0 | 0 | 10 |
| *Carex rariflora* | 0 | 10 | 0 | 0 | 0 | 0 | 10 |
| *Puccinellia phryganodes* | 0 | 10 | 0 | 0 | 0 | 0 | 10 |
| *Stellaria humifusa* | 10 | 0 | 0 | 0 | 0 | 0 | 10 |

## Brackish sedge-willow marsh (BSWM)

Figure 2. Brackish sedge-willow marsh, plot B-20180706-04, barrier island of Cowpack Lagoon, BELA. NPS photo, public domain.

**USNVC Group:** Not classified.

**Plant association:** *Salix ovalifolia - Carex rariflora;* *Carex rariflora-Salix ovalifolia-Empetrum nigrum* [syn. *E. hermaphroditum*] [5,6]

**Alaska Vegetation Class:** III.A.3.i - Halophytic wet sedge meadow

**ARCN Landcover Ecotype:** Coastal Brackish Willow Shrub.

Found in moist areas at lower salinity than salt marshes. Typically, these are either found in protected swales surrounded by beach ridges or at slightly higher elevations transitioning from salt marsh. Brackish sedge-willow marshes do not have standing water. They are dominated by the minute sedge *Carex rariflora* and the creeping dwarf willow *Salix ovalifolia*. This type transitions to wet sedge meadows of *Eriophorum angustifolium*-*Carex aquatilis* (Fresh-to-brackish wet sedge meadow, FBSWM) with decreasing salinity.

Table 3. Site variable summary for Brackish Sedge-Willow Marsh (n=74).

|  | **Median** | **IQR** |
| --- | --- | --- |
| EC (µS/m) | 1373 | 2389.5 |
| Hydric index | 2.1 | 0.5 |
| Elevation (m) | 4.4 | 1.3 |
| Distance to ocean (km) | 0.6 | 5.8 |
| Distance to estuary (km) | 0.3 | 0.6 |
| *Functional group cover (%)* | | |
| Graminoid | 35 | 25 |
| Forb | 2 | 5 |
| Dwarf shrub | 20 | 34.5 |
| Low shrub | 0 | 0 |
| Tall shrub | 0 | 0 |
| Lichen | 0 | 0 |
| Moss | 0 | 2 |

Table 4. Species cover and frequency for Brackish Sedge-Willow Marsh (n=74).

|  | Cover Class Frequency (%) | | | | | |  |
| --- | --- | --- | --- | --- | --- | --- | --- |
|  | 0:<1% | 1:1-5% | 2:6-25% | 3:26-50% | 4:51-75% | 5:76-100% | **Total Frequency (%)** |
| *Salix ovalifolia* | 1 | 24 | 29 | 17 | 5 | 1 | 77 |
| *Carex rariflora* | 6 | 12 | 23 | 24 | 8 | 0 | 73 |
| *Chrysanthemum arcticum* | 29 | 18 | 1 | 0 | 0 | 0 | 48 |
| *Empetrum hermaphroditum* | 12 | 20 | 7 | 7 | 1 | 0 | 48 |
| *Eriophorum angustifolium* | 5 | 19 | 18 | 6 | 0 | 0 | 48 |
| *Calamagrostis deschampsioides* | 11 | 19 | 7 | 4 | 0 | 0 | 41 |
| *Rumex arcticus* | 30 | 2 | 0 | 0 | 0 | 0 | 33 |
| *Rhodiola integrifolia* | 23 | 5 | 0 | 0 | 0 | 0 | 28 |
| *Salix fuscescens* | 1 | 10 | 7 | 4 | 0 | 0 | 22 |
| *Carex glareosa* | 2 | 13 | 4 | 1 | 0 | 0 | 20 |
| *Carex ramenskii* | 1 | 4 | 8 | 6 | 1 | 0 | 20 |
| *Dupontia fisheri* | 4 | 11 | 4 | 1 | 0 | 0 | 19 |
| *Leymus mollis* | 14 | 4 | 1 | 0 | 0 | 0 | 19 |
| *Carex aquatilis* | 4 | 10 | 2 | 0 | 0 | 0 | 16 |
| *Cerastium beeringianum* | 14 | 1 | 0 | 0 | 0 | 0 | 16 |
| Moss, other | 0 | 5 | 8 | 2 | 0 | 0 | 16 |
| *Pedicularis* sp. | 16 | 0 | 0 | 0 | 0 | 0 | 16 |
| *Petasites frigidus* | 10 | 5 | 0 | 0 | 0 | 0 | 14 |
| *Saussurea nuda* | 8 | 5 | 1 | 0 | 0 | 0 | 14 |
| *Stellaria humifusa* | 11 | 4 | 0 | 0 | 0 | 0 | 14 |
| *Saxifraga radiata* | 10 | 4 | 0 | 0 | 0 | 0 | 13 |
| *Carex subspathacea* | 1 | 6 | 4 | 0 | 1 | 0 | 12 |
| *Cochlearia officinalis* | 7 | 5 | 0 | 0 | 0 | 0 | 12 |
| *Juncus arcticus* | 5 | 4 | 1 | 1 | 0 | 0 | 11 |
| *Polemonium acutiflorum* | 10 | 1 | 0 | 0 | 0 | 0 | 11 |
| *Sphagnum* sp. | 2 | 6 | 2 | 0 | 0 | 0 | 11 |
| *Betula nana* | 5 | 5 | 0 | 0 | 0 | 0 | 10 |

## Crowberry lichen tundra (CLT)

Figure 3. Crowberry lichen tundra, plot A-20180704-11, barrier island of Cowpack Lagoon, BELA. NPS photo, public domain.

**USNVC Group:** Not classified.

**Plant association:** *Empetrum nigrum-Leymus mollis* [1]; *Empetrum hermaphroditum/Thamnolia vermicularis-Flavocetraria cucullata*

**Alaska Vegetation Class**: II.D.2.c. Crowberry Dwarf Shrub Tundra

**ARCN Landcover Ecotype:** Coastal Crowberry Dwarf Shrub

Dominated by crowberry (*Empetrum hermaphroditum*) and lichens such as *Flavocetraria cucullata*, *Thamnolia vermicularis, Stereocaulon spp., Flavocetraria nivalis, Bryocaulon divergens,* and *Alectoria nigricans*, this type is found on stabilized beach ridges. Beach ridges farther from the ocean typically have higher lichen cover, presumably a function of age. Newly stabilized beach ridges frequently have just a few species. We include some plots from the Cape Krusenstern beach ridge complex that are not dominated by crowberry, but maintain much of the same species pool, high lichen cover and have similar reflectance in the imagery.

Table 5. Site variable summary for Crowberry Lichen Tundra (n=43).

|  | **Median** | **IQR** |
| --- | --- | --- |
| EC (µS/m) | 30 | 79.5 |
| Hydric index | 3.3 | 0.7 |
| Elevation (m) | 4.9 | 1.9 |
| Distance to ocean (km) | 0.2 | 0.5 |
| Distance to estuary (km) | 0.4 | 0.5 |
| *Functional group cover (%)* | | |
| Graminoid | 3 | 7.5 |
| Forb | 2 | 6 |
| Dwarf shrub | 48 | 33 |
| Low shrub | 0 | 0.5 |
| Tall shrub | 0 | 0 |
| Lichen | 10 | 31.5 |
| Moss | 0 | 6 |

Table 6. Species cover and frequency for Crowberry Lichen Tundra (n=43).

|  | Cover Class Frequency (%) | | | | | |  |
| --- | --- | --- | --- | --- | --- | --- | --- |
|  | 0:<1% | 1:1-5% | 2:6-25% | 3:26-50% | 4:51-75% | 5:76-100% | **Total Frequency (%)** |
| *Empetrum hermaphroditum* | 0 | 9 | 27 | 40 | 15 | 9 | 100 |
| *Leymus mollis* | 13 | 44 | 11 | 0 | 0 | 0 | 67 |
| *Thamnolia vermicularis* | 11 | 40 | 2 | 0 | 0 | 0 | 53 |
| *Flavocetraria cucullata* | 18 | 24 | 4 | 4 | 0 | 0 | 49 |
| *Armeria maritima* | 35 | 5 | 0 | 0 | 0 | 0 | 40 |
| *Lathyrus maritimus* | 11 | 15 | 15 | 0 | 0 | 0 | 40 |
| *Vaccinium uliginosum* | 13 | 16 | 7 | 0 | 0 | 0 | 36 |
| *Stereocaulon* sp. | 16 | 16 | 2 | 0 | 0 | 0 | 35 |
| *Arctous alpina* | 16 | 16 | 0 | 0 | 0 | 0 | 33 |
| *Flavocetraria nivalis* | 11 | 22 | 0 | 0 | 0 | 0 | 33 |
| Moss, other | 2 | 18 | 11 | 2 | 0 | 0 | 33 |
| *Salix ovalifolia* | 7 | 15 | 9 | 0 | 0 | 0 | 31 |
| *Lobaria linita* | 25 | 4 | 0 | 0 | 0 | 0 | 29 |
| *Cladonia amaurocraea* | 9 | 15 | 4 | 0 | 0 | 0 | 27 |
| *Sphaerophorus globosus* | 22 | 5 | 0 | 0 | 0 | 0 | 27 |
| *Salix arctica* | 4 | 5 | 7 | 4 | 4 | 0 | 24 |
| *Bryocaulon divergens* | 4 | 13 | 5 | 0 | 0 | 0 | 22 |
| *Cetraria laevigata* | 22 | 0 | 0 | 0 | 0 | 0 | 22 |
| *Cladina arbuscula/mitis* | 4 | 13 | 5 | 0 | 0 | 0 | 22 |
| *Cladonia* sp. | 4 | 11 | 7 | 0 | 0 | 0 | 22 |
| *Vaccinium vitis-idaea* | 5 | 13 | 4 | 0 | 0 | 0 | 22 |
| *Betula nana* | 5 | 11 | 4 | 0 | 0 | 0 | 20 |
| *Cetraria ericetorum/islandica/laevigata* | 0 | 5 | 11 | 4 | 0 | 0 | 20 |
| *Artemisia arctica* | 11 | 5 | 0 | 0 | 0 | 0 | 16 |
| *Chrysanthemum bipinnatum* | 13 | 4 | 0 | 0 | 0 | 0 | 16 |
| *Alectoria nigricans* | 4 | 11 | 0 | 0 | 0 | 0 | 15 |
| *Dicranum* sp. | 0 | 13 | 2 | 0 | 0 | 0 | 15 |
| *Festuca rubra* | 11 | 4 | 0 | 0 | 0 | 0 | 15 |
| *Oxytropis maydelliana* | 11 | 4 | 0 | 0 | 0 | 0 | 15 |
| *Salix glauca* | 4 | 4 | 5 | 2 | 0 | 0 | 15 |
| *Sphaerophorus* sp. | 2 | 7 | 5 | 0 | 0 | 0 | 15 |
| *Bupleurum americanum* | 11 | 2 | 0 | 0 | 0 | 0 | 13 |
| *Carex rariflora* | 0 | 4 | 9 | 0 | 0 | 0 | 13 |
| *Cladina rangiferina/stygia* | 0 | 9 | 4 | 0 | 0 | 0 | 13 |
| *Epilobium latifolium* | 4 | 5 | 4 | 0 | 0 | 0 | 13 |
| *Ledum palustre* | 4 | 7 | 2 | 0 | 0 | 0 | 13 |
| *Lobaria* sp. | 0 | 7 | 5 | 0 | 0 | 0 | 13 |
| *Pedicularis* sp. | 13 | 0 | 0 | 0 | 0 | 0 | 13 |
| *Trisetum spicatum* | 11 | 2 | 0 | 0 | 0 | 0 | 13 |
| *Alectoria ochroleuca* | 7 | 2 | 2 | 0 | 0 | 0 | 11 |
| *Artemisia tilesii* | 7 | 4 | 0 | 0 | 0 | 0 | 11 |
| *Castilleja elegans* | 9 | 2 | 0 | 0 | 0 | 0 | 11 |
| *Cladonia uncialis* | 9 | 2 | 0 | 0 | 0 | 0 | 11 |
| *Ochrolechia frigida* | 4 | 7 | 0 | 0 | 0 | 0 | 11 |
| *Poa* sp. | 11 | 0 | 0 | 0 | 0 | 0 | 11 |
| *Polytrichum* sp. | 5 | 4 | 2 | 0 | 0 | 0 | 11 |

## Deciduous low shrub tundra (DLST)

Figure 4. Typical deciduous low shrub tundra, plot A-20180710-02, upper Goodhope River. NPS photo, public domain.

**USNVC Group:** G356 *Betula nana - Salix pulchra* Mesic Low Shrubland Group

**Plant association:** *Betula glandulosa/Vaccinium uliginosum-Empetrum nigrum-Ledum*

*decumbens/*lichens [7,8]

**Alaska Vegetation Class:** II.C.2.c. Open Low Mesic Shrub Birch-Ericaceous Shrub

**ARCN Landcover Ecotype:** Lowland Birch–Ericaceous Low Shrub

A freshwater type, dominated by *Betula nana*, *Ledum decumbens* subsp. *palustre*, and *Vaccinium uliginosum*. Other common species include *Rubus chamaemorus, Empetrum hermaphroditum, Vaccinium vitis-idaea* and *Salix pulchra*. Typical mosses were *Pleurozium schreberi* and various *Sphagnum* species. Though not strictly a coastal type, it is included in the classification because it is found, like *Carex aquatilis ­*– *Eriophorum angustifolium* wetlands, in protected areas between beach ridges of the study area.

Table 7. Site variable summary for Deciduous Low Shrub Tundra (DLST) (n=25).

|  | **Median** | **IQR** |
| --- | --- | --- |
| EC (µS/m) | 150 | 114.8 |
| Hydric index | 2.9 | 0.7 |
| Elevation (m) | 4.8 | 1.7 |
| Distance to ocean (km) | 1.1 | 1.1 |
| Distance to estuary (km) | 1.3 | 2 |
| *Functional group cover (%)* | | |
| Graminoid | 4 | 5.2 |
| Forb | 2.5 | 7.2 |
| Dwarf shrub | 48.5 | 26.8 |
| Low shrub | 11 | 34.8 |
| Tall shrub | 0 | 1 |
| Lichen | 5.5 | 28 |
| Moss | 16.5 | 36.2 |

Table 8. Species cover and frequency for Deciduous Low Shrub Tundra (DLST) (n=25).

|  | Cover Class Frequency (%) | | | | | |
| --- | --- | --- | --- | --- | --- | --- |
|  | 0:<1% | 1:1-5% | 2:6-25% | 3:26-50% | 4:51-75% | **Total Frequency (%)** |
| *Betula nana* | 0 | 25 | 58 | 17 | 0 | 100 |
| *Empetrum hermaphroditum* | 0 | 25 | 46 | 21 | 0 | 92 |
| *Ledum palustre* | 12 | 38 | 21 | 12 | 0 | 83 |
| *Vaccinium uliginosum* | 4 | 33 | 38 | 0 | 0 | 75 |
| *Vaccinium vitis-idaea* | 8 | 25 | 29 | 12 | 0 | 75 |
| *Flavocetraria cucullata* | 12 | 38 | 21 | 0 | 0 | 71 |
| *Rubus chamaemorus* | 8 | 29 | 25 | 0 | 0 | 62 |
| *Salix pulchra* | 12 | 29 | 17 | 0 | 4 | 62 |
| *Dicranum* sp. | 8 | 21 | 21 | 0 | 0 | 50 |
| *Sphagnum* sp. | 0 | 12 | 17 | 17 | 4 | 50 |
| Moss, other | 4 | 25 | 12 | 0 | 0 | 42 |
| *Arctous alpina* | 8 | 17 | 12 | 0 | 0 | 38 |
| *Carex aquatilis* | 0 | 17 | 21 | 0 | 0 | 38 |
| *Polytrichum* sp. | 8 | 21 | 8 | 0 | 0 | 38 |
| *Aulacomnium palustre/acuminatum* | 0 | 25 | 8 | 0 | 0 | 33 |
| *Aulacomnium turgidum* | 0 | 29 | 4 | 0 | 0 | 33 |
| *Carex bigelowii* | 12 | 21 | 0 | 0 | 0 | 33 |
| *Cetraria ericetorum/islandica/laevigata* | 0 | 12 | 21 | 0 | 0 | 33 |
| *Pleurozium schreberi* | 4 | 17 | 12 | 0 | 0 | 33 |
| *Andromeda polifolia* | 4 | 17 | 8 | 0 | 0 | 29 |
| *Cetraria laevigata* | 21 | 8 | 0 | 0 | 0 | 29 |
| *Hylocomium splendens* | 0 | 17 | 12 | 0 | 0 | 29 |
| *Thamnolia vermicularis* | 4 | 25 | 0 | 0 | 0 | 29 |
| *Cassiope tetragona* | 21 | 4 | 0 | 0 | 0 | 25 |
| *Cladina arbuscula/mitis* | 8 | 17 | 0 | 0 | 0 | 25 |
| *Cladonia* sp. | 0 | 8 | 17 | 0 | 0 | 25 |
| Unknown lichen | 0 | 21 | 0 | 0 | 0 | 21 |
| *Alectoria nigricans* | 12 | 8 | 0 | 0 | 0 | 21 |
| *Bryocaulon divergens* | 4 | 17 | 0 | 0 | 0 | 21 |
| *Cladina rangiferina/stygia* | 0 | 21 | 0 | 0 | 0 | 21 |
| *Cladonia amaurocraea* | 4 | 17 | 0 | 0 | 0 | 21 |
| *Flavocetraria nivalis* | 8 | 8 | 4 | 0 | 0 | 21 |
| *Lobaria linita* | 17 | 4 | 0 | 0 | 0 | 21 |
| *Salix reticulata* | 8 | 8 | 4 | 0 | 0 | 21 |
| *Carex rariflora* | 4 | 12 | 0 | 0 | 0 | 17 |
| *Loiseleuria procumbens* | 0 | 12 | 4 | 0 | 0 | 17 |
| *Oxycoccus microcarpos* | 4 | 8 | 4 | 0 | 0 | 17 |
| *Petasites frigidus* | 8 | 8 | 0 | 0 | 0 | 17 |
| *Poa arctica* | 8 | 8 | 0 | 0 | 0 | 17 |
| *Rhytidium rugosum* | 0 | 4 | 12 | 0 | 0 | 17 |
| *Arctous rubra* | 4 | 8 | 0 | 0 | 0 | 12 |
| *Asahinea chrysantha* | 12 | 0 | 0 | 0 | 0 | 12 |
| *Calamagrostis canadensis* | 4 | 8 | 0 | 0 | 0 | 12 |
| *Cladonia gracilis* | 12 | 0 | 0 | 0 | 0 | 12 |
| *Hierochloe alpina* | 12 | 0 | 0 | 0 | 0 | 12 |
| *Pedicularis* sp. | 8 | 4 | 0 | 0 | 0 | 12 |
| *Peltigera aphthosa* | 4 | 8 | 0 | 0 | 0 | 12 |
| *Sphaerophorus globosus* | 8 | 4 | 0 | 0 | 0 | 12 |

## Dunegrass beach meadow (DBM)

Figure 5. A densely vegetated dunegrass beach meadow on a sandy beach ridge on Cowpack Lagoon, with sparse dunes in the background, transitioning to crowberry tundra. NPS photo, public domain.

**USNVC Group:** G612 *Leymus mollis* - *Honckenya peploides* - *Lathyrus japonicus* var. *maritimus* [syn. *Lathyrus maritimus*] Beach & Dune Group

**Plant associations:** *Elymus* *arenarius* [syn. *Leymus mollis*] [5,8–10]; *Elymus arenarius-Lathyrus maritimus* [5,10].

**Alaska Vegetation Class:** IlI.A.1.a. *Elymus* [syn. *Leymus*]

**ARCN Landcover Ecotype:** Coastal Brackish Dunegrass Meadow

*Leymus mollis* is the pioneer species of the arctic coast, the first plant to colonize open sediment and stabilize beach dunes. In the sandy barrier islands of BELA where breaches into the lagoons are common and wind action can disturb sediments, dunegrass communities are common in strips along blowouts. This type is present in CAKR directly along the beach front, and in the inner beach of larger lagoons. Dunegrass meadows vary from sparsely to densely vegetated, and transition to crowberry tundra.

Associated species in both parks include *Lathyrus maritimus*, *Poa eminens* and *Honckenya peploides*. Unique to our BELA plots: *Rhodiola integrifolia*. Unique to CAKR: *Senecio pseudo-arnica*.

Table 9.

|  | **Median** | **IQR** |
| --- | --- | --- |
| EC (µS/m) | 17 | 57 |
| Hydric index | 3.3 | 0.5 |
| Elevation (m) | 5.2 | 1.4 |
| Distance to ocean (km) | 0.1 | 0 |
| Distance to estuary (km) | 0.4 | 1.9 |
| *Functional group cover (%)* | | |
| Graminoid | 17.5 | 18 |
| Forb | 9 | 22.8 |
| Dwarf shrub | 0 | 0 |
| Low shrub | 0 | 0 |
| Tall shrub | 0 | 0 |
| Lichen | 0 | 0 |
| Moss | 0 | 0 |

Table 10. Species cover and frequency for Dunegrass Beach Meadow (DBM) (n=33).

|  | Cover Class Frequency (%) | | | | | |
| --- | --- | --- | --- | --- | --- | --- |
|  | 0:<1% | 1:1-5% | 2:6-25% | 3:26-50% | 4:51-75% | Total Frequency (%) |
| *Leymus mollis* | 0 | 25 | 58 | 17 | 0 | 100 |
| *Lathyrus maritimus* | 0 | 25 | 46 | 21 | 0 | 92 |
| *Honckenya peploides* | 12 | 38 | 21 | 12 | 0 | 83 |
| *Poa eminens* | 4 | 33 | 38 | 0 | 0 | 75 |
| *Festuca rubra* | 8 | 25 | 29 | 12 | 0 | 75 |
| *Senecio pseudo-arnica* | 12 | 38 | 21 | 0 | 0 | 71 |
| *Artemisia tilesii* | 8 | 29 | 25 | 0 | 0 | 62 |
| *Chrysanthemum arcticum* | 12 | 29 | 17 | 0 | 4 | 62 |
| Moss, other | 8 | 21 | 21 | 0 | 0 | 50 |
| *Conioselinum pacificum* | 0 | 12 | 17 | 17 | 4 | 50 |

## Fresh-to-brackish wet sedge meadow (FBWSM)

Figure 6. An *Eriophorum angustifolium* dominated plot, A-20180717-11, Imik Lagoon, CAKR. NPS photo, public domain.

**USNVC Group:**  G617 *Carex aquatilis* - *Eriophorum angustifolium* Arctic & Subarctic Wet Meadow Group

**Plant association:** *Carex aquatilis-Eriophorum angustifolium* [7,10]

**Alaska Vegetation Class:** II.A.3.a Wet sedge meadow tundra

**ARCN Landcover Ecotype:** Most similar to Lowland Sedge Fen, though we did not see the dominance of *Carex chordorrhiza* in our plots.

These wetlands range from brackish to fresh. This vegetation type is typical of the wet inter-polygons of the surrounding uplands. It is included on the map in particularly protected inter-dune areas and in the transition from brackish to freshwater types.

Table 11. Site variable summary for Fresh-to-Brackish Wet Sedge Meadow (FBWSM) (n=26).

|  | **Median** | **IQR** |
| --- | --- | --- |
| EC (µS/m) | 340 | 974.5 |
| Hydric index | 1.8 | 0.5 |
| Elevation (m) | 4.6 | 1.6 |
| Distance to ocean (km) | 0.6 | 1.5 |
| Distance to estuary (km) | 1.1 | 2.2 |
| *Functional group cover (%)* | | |
| Graminoid | 29 | 20 |
| Forb | 1 | 5 |
| Dwarf shrub | 8 | 25 |
| Low shrub | 0 | 0 |
| Tall shrub | 0 | 0 |
| Lichen | 0 | 0 |
| Moss | 5 | 17 |

Table 12. Species cover and frequency for Fresh-to-Brackish Wet Sedge Meadow (FBWSM) (n=26).

|  | Cover Class Frequency (%) | | | | | |
| --- | --- | --- | --- | --- | --- | --- |
|  | 0:<1% | 1:1-5% | 2:6-25% | 3:26-50% | 4:51-75% | **Total Frequency (%)** |
| *Carex aquatilis* | 4 | 0 | 59 | 26 | 7 | 96 |
| *Eriophorum angustifolium* | 7 | 11 | 11 | 30 | 0 | 59 |
| *Salix fuscescens* | 4 | 15 | 22 | 4 | 0 | 44 |
| *Carex rariflora* | 7 | 30 | 4 | 0 | 0 | 41 |
| *Comarum palustre* | 15 | 15 | 7 | 4 | 0 | 41 |
| *Sphagnum* sp. | 11 | 11 | 15 | 4 | 0 | 41 |
| *Betula nana* | 4 | 26 | 4 | 0 | 0 | 33 |
| *Andromeda polifolia* | 11 | 11 | 7 | 0 | 0 | 30 |
| *Empetrum hermaphroditum* | 7 | 22 | 0 | 0 | 0 | 30 |
| *Polemonium acutiflorum* | 22 | 7 | 0 | 0 | 0 | 30 |
| *Salix pulchra* | 4 | 11 | 7 | 4 | 0 | 26 |
| *Cardamine pratensis* | 22 | 0 | 0 | 0 | 0 | 22 |
| *Dupontia fisheri* | 7 | 15 | 0 | 0 | 0 | 22 |
| *Rumex arcticus* | 15 | 7 | 0 | 0 | 0 | 22 |
| *Saxifraga hirculus* | 19 | 4 | 0 | 0 | 0 | 22 |
| *Vaccinium uliginosum* | 15 | 7 | 0 | 0 | 0 | 22 |
| *Aulacomnium palustre/acuminatum* | 11 | 7 | 0 | 0 | 0 | 19 |
| *Calamagrostis canadensis* | 0 | 7 | 11 | 0 | 0 | 19 |
| *Eriophorum chamissonis* | 0 | 15 | 0 | 0 | 0 | 15 |
| *Ledum palustre* | 4 | 11 | 0 | 0 | 0 | 15 |
| Moss, other | 0 | 11 | 4 | 0 | 0 | 15 |
| *Petasites frigidus* | 0 | 15 | 0 | 0 | 0 | 15 |
| *Salix ovalifolia* | 4 | 7 | 4 | 0 | 0 | 15 |
| *Salix richardsonii* | 0 | 4 | 7 | 4 | 0 | 15 |
| *Valeriana capitata* | 7 | 7 | 0 | 0 | 0 | 15 |
| *Bistorta vivipara* | 11 | 0 | 0 | 0 | 0 | 11 |
| *Caltha palustris* | 11 | 0 | 0 | 0 | 0 | 11 |
| *Carex chordorrhiza* | 0 | 11 | 0 | 0 | 0 | 11 |
| *Carex rotundata* | 4 | 7 | 0 | 0 | 0 | 11 |
| *Galium brandegeei* | 11 | 0 | 0 | 0 | 0 | 11 |
| *Pedicularis* sp. | 11 | 0 | 0 | 0 | 0 | 11 |
| *Rhodiola integrifolia* | 11 | 0 | 0 | 0 | 0 | 11 |
| *Rubus chamaemorus* | 0 | 11 | 0 | 0 | 0 | 11 |

## Grayleaf willow shrub (GWS)

Figure 7. Plot A-20180714-09, west of the Tukrok River, outer beach of CAKR. NPS photo, public domain.

**USNVC Group:** Not yet classified, most similar to G368 *Salix alaxensis* Arctic Wet Shrubland Group.

**Plant association:** None proposed. See notes below.

**Alaska Vegetation Class:** II.C.2.g. Open Low Willow Shrub

**ARCN Landcover Ecotype:** Most similar to Riverine Willow Low Shrub and Coastal Crowberry Dwarf Shrub.

This type is limited to a few sites along the outer beach ridges of Cape Krusenstern, where it appears to replace coastal crowberry tundra. We saw several distinct suites of co-occurring plants, but have too few samples to adequately describe these as plant associations. We include it as a distinct class because the presence of tall shrubs makes it highly visible in the imagery and possible to map.

Table 13. Site variable summary for Grayleaf Willow Shrub (GWS) (n=5).

|  | **Median** | **IQR** |
| --- | --- | --- |
| EC (µS/m) | 459 | 3207 |
| Hydric index | 2.9 | 0.7 |
| Elevation (m) | 4.7 | 0.6 |
| Distance to ocean (km) | 2.3 | 2.8 |
| Distance to estuary (km) | 0.1 | 1.4 |
| *Functional group cover (%)* | | |
| Graminoid | 5 | 19 |
| Forb | 1 | 5 |
| Dwarf shrub | 29 | 44 |
| Low shrub | 15 | 23 |
| Tall shrub | 0 | 0 |
| Lichen | 0 | 0 |
| Moss | 0 | 1 |

Table 14. Species cover and frequency for Grayleaf Willow Shrub (GWS) (n=5).

|  | Cover Class Frequency (%) | | | | | |  |
| --- | --- | --- | --- | --- | --- | --- | --- |
|  | 0:<1% | 1:1-5% | 2:6-25% | 3:26-50% | 4:51-75% | Total Frequency (%) | |
| *Salix glauca* | 0 | 0 | 40 | 40 | 20 | 100 | |
| *Salix ovalifolia* | 20 | 20 | 0 | 20 | 0 | 60 | |
| *Dupontia fisheri* | 20 | 20 | 0 | 0 | 0 | 40 | |
| *Empetrum hermaphroditum* | 0 | 0 | 0 | 0 | 40 | 40 | |
| *Eriophorum angustifolium* | 0 | 40 | 0 | 0 | 0 | 40 | |
| *Leymus mollis* | 20 | 20 | 0 | 0 | 0 | 40 | |
| *Petasites frigidus* | 20 | 20 | 0 | 0 | 0 | 40 | |
| *Salix richardsonii* | 0 | 0 | 20 | 20 | 0 | 40 | |
| *Vaccinium uliginosum* | 20 | 20 | 0 | 0 | 0 | 40 | |
| *Androsace chamaejasme* | 20 | 0 | 0 | 0 | 0 | 20 | |
| *Arctagrostis latifolia* | 20 | 0 | 0 | 0 | 0 | 20 | |
| *Arctous rubra* | 20 | 0 | 0 | 0 | 0 | 20 | |
| *Astragalus alpinus* | 20 | 0 | 0 | 0 | 0 | 20 | |
| *Betula nana* | 0 | 20 | 0 | 0 | 0 | 20 | |
| *Calamagrostis stricta* | 0 | 0 | 0 | 20 | 0 | 20 | |
| *Carex bigelowii* | 20 | 0 | 0 | 0 | 0 | 20 | |
| *Castilleja elegans* | 20 | 0 | 0 | 0 | 0 | 20 | |
| *Cladonia pyxidata* | 0 | 20 | 0 | 0 | 0 | 20 | |
| *Comarum palustre* | 20 | 0 | 0 | 0 | 0 | 20 | |
| *Deschampsia cespitosa* | 0 | 20 | 0 | 0 | 0 | 20 | |
| *Epilobium latifolium* | 20 | 0 | 0 | 0 | 0 | 20 | |
| *Equisetum arvense* | 0 | 20 | 0 | 0 | 0 | 20 | |
| *Equisetum variegatum* | 0 | 20 | 0 | 0 | 0 | 20 | |
| *Festuca rubra* | 20 | 0 | 0 | 0 | 0 | 20 | |
| *Lathyrus maritimus* | 20 | 0 | 0 | 0 | 0 | 20 | |
| Moss, other | 0 | 0 | 20 | 0 | 0 | 20 | |
| *Orthilia secunda* | 20 | 0 | 0 | 0 | 0 | 20 | |
| *Parmelia omphalodes* | 20 | 0 | 0 | 0 | 0 | 20 | |
| *Parnassia palustris* | 20 | 0 | 0 | 0 | 0 | 20 | |
| *Potentilla villosula* | 20 | 0 | 0 | 0 | 0 | 20 | |
| *Primula* sp. | 20 | 0 | 0 | 0 | 0 | 20 | |
| *Rumex arcticus* | 20 | 0 | 0 | 0 | 0 | 20 | |
| *Salix pulchra* | 0 | 0 | 20 | 0 | 0 | 20 | |
| *Salix reticulata* | 20 | 0 | 0 | 0 | 0 | 20 | |
| *Saxifraga hirculus* | 20 | 0 | 0 | 0 | 0 | 20 | |
| *Senecio lugens* | 20 | 0 | 0 | 0 | 0 | 20 | |

## Herbaceous dry beach ridge meadow (HDBRM)

Figure 8. A forb-dominated beach ridge, upper Krusenstern Lagoon in CAKR, plot A-20180716-13. NPS photo, public domain.

**USNVC Group:** Not classified.

**Plant association:** *Epilobium latifolium – Saxifraga tricuspidata – Artemisia tilesii*

**Alaska Vegetation Class:** Similar to Ill.B.1.a. Seral Herbs

**ARCN Landcover Ecotype:** Not classified.

Found exclusively along the beach ridges of CAKR, this type is not denoted by the dominance of any one species. Instead, it is separated from crowberry lichen tundra by the abundance and diversity of herbaceous flowering plants. Common species included *Epilobium latifolium*, *Artemisia tilesii*, *Saxifraga tricuspidata* and *Conioselinum pacificum*. Older beach ridges transition to crowberry lichen tundra.

Table 15. Site variable summary for Herbaceous Dry Beach Ridge Meadow (HDBRM) (n=16).

|  | **Median** | **IQR** |
| --- | --- | --- |
| EC (µS/m) | 11 | 13 |
| Hydric index | 3.5 | 0.3 |
| Elevation (m) | 4.2 | 0.6 |
| Distance to ocean (km) | 0.2 | 0.4 |
| Distance to estuary (km) | 0.1 | 1.7 |
| *Functional group cover (%)* | | |
| Graminoid | 15 | 15 |
| Forb | 35 | 22 |
| Dwarf shrub | 2 | 4 |
| Low shrub | 0 | 0 |
| Tall shrub | 0 | 0 |
| Lichen | 2 | 10 |
| Moss | 5 | 9 |

Table 16. Species cover and frequency for Herbaceous Dry Beach Ridge Meadow (HDBRM) (n=16).

|  | Cover Class Frequency (%) | | | |  |
| --- | --- | --- | --- | --- | --- |
|  | 0:<1% | 1:1-5% | 2:6-25% | 3:26-50% | **Total Frequency (%)** |
| *Epilobium latifolium* | 35 | 29 | 29 | 0 | 94 |
| *Saxifraga tricuspidata* | 24 | 47 | 18 | 0 | 88 |
| *Artemisia tilesii* | 6 | 53 | 24 | 0 | 82 |
| *Bupleurum americanum* | 53 | 24 | 0 | 0 | 76 |
| *Conioselinum pacificum* | 47 | 24 | 6 | 0 | 76 |
| *Lathyrus maritimus* | 0 | 12 | 59 | 0 | 71 |
| *Empetrum hermaphroditum* | 0 | 47 | 12 | 6 | 65 |
| *Festuca rubra* | 6 | 47 | 6 | 0 | 59 |
| *Leymus mollis* | 0 | 12 | 41 | 6 | 59 |
| *Carex gmelinii* | 29 | 18 | 6 | 0 | 53 |
| *Astragalus alpinus* | 12 | 29 | 6 | 0 | 47 |
| *Rhytidium rugosum* | 6 | 12 | 18 | 12 | 47 |
| *Senecio lugens* | 24 | 24 | 0 | 0 | 47 |
| *Androsace chamaejasme* | 29 | 12 | 0 | 0 | 41 |
| *Cerastium beeringianum* | 35 | 6 | 0 | 0 | 41 |
| *Thamnolia subuliformis/vermicularis* | 6 | 24 | 12 | 0 | 41 |
| *Trisetum spicatum* | 29 | 12 | 0 | 0 | 41 |
| *Artemisia arctica* | 18 | 18 | 0 | 0 | 35 |
| *Flavocetraria cucullata* | 12 | 24 | 0 | 0 | 35 |
| *Hylocomium splendens* | 12 | 18 | 6 | 0 | 35 |
| *Angelica lucida* | 12 | 18 | 0 | 0 | 29 |
| *Armeria maritima* | 24 | 6 | 0 | 0 | 29 |
| *Dicranum* sp. | 6 | 24 | 0 | 0 | 29 |
| *Flavocetraria nivalis* | 12 | 12 | 0 | 0 | 24 |
| *Iris setosa* | 24 | 0 | 0 | 0 | 24 |
| *Lobaria linita* | 24 | 0 | 0 | 0 | 24 |
| Moss, other | 0 | 12 | 12 | 0 | 24 |
| *Papaver lapponicum* | 18 | 6 | 0 | 0 | 24 |
| *Papaver* sp. | 24 | 0 | 0 | 0 | 24 |
| *Poa arctica* | 0 | 18 | 6 | 0 | 24 |
| *Polytrichum* sp. | 0 | 18 | 6 | 0 | 24 |
| *Potentilla villosula* | 18 | 6 | 0 | 0 | 24 |
| *Selaginella sibirica* | 6 | 0 | 12 | 6 | 24 |
| *Silene acaulis* | 24 | 0 | 0 | 0 | 24 |
| *Sphaerophorus globosus* | 6 | 12 | 6 | 0 | 24 |
| *Stellaria* sp. | 12 | 12 | 0 | 0 | 24 |
| *Asahinea chrysantha* | 18 | 0 | 0 | 0 | 18 |
| *Cladonia amaurocraea* | 6 | 12 | 0 | 0 | 18 |
| *Ligusticum scoticum* | 12 | 0 | 0 | 6 | 18 |
| *Oxytropis maydelliana* | 12 | 6 | 0 | 0 | 18 |
| *Poa glauca* | 0 | 12 | 6 | 0 | 18 |
| *Poa* sp. | 18 | 0 | 0 | 0 | 18 |
| *Senecio pseudo-arnica* | 12 | 0 | 6 | 0 | 18 |
| *Stereocaulon* sp. | 6 | 12 | 0 | 0 | 18 |
| *Aulacomnium palustre/acuminatum* | 0 | 6 | 6 | 0 | 12 |
| *Bryocaulon divergens* | 6 | 6 | 0 | 0 | 12 |
| *Cetraria laevigata* | 12 | 0 | 0 | 0 | 12 |
| *Cladina arbuscula/mitis* | 0 | 12 | 0 | 0 | 12 |
| *Cladonia pyxidata* | 6 | 0 | 6 | 0 | 12 |
| *Cladonia* sp. | 0 | 12 | 0 | 0 | 12 |
| *Dactylina arctica/beringica* | 12 | 0 | 0 | 0 | 12 |
| *Draba glabella* | 12 | 0 | 0 | 0 | 12 |
| *Epilobium angustifolium* | 6 | 0 | 6 | 0 | 12 |
| *Honckenya peploides* | 6 | 6 | 0 | 0 | 12 |
| *Mertensia maritima* | 0 | 6 | 6 | 0 | 12 |
| *Ochrolechia frigida* | 0 | 6 | 6 | 0 | 12 |
| *Peltigera britannica* | 12 | 0 | 0 | 0 | 12 |
| *Poa eminens* | 6 | 6 | 0 | 0 | 12 |
| *Polemonium acutiflorum* | 12 | 0 | 0 | 0 | 12 |
| *Potentilla litoralis* | 12 | 0 | 0 | 0 | 12 |
| *Salix ovalifolia* | 6 | 0 | 6 | 0 | 12 |
| *Saxifraga bronchialis* | 6 | 6 | 0 | 0 | 12 |
| *Stellaria longipes* | 12 | 0 | 0 | 0 | 12 |

## Lyngbye’s sedge marsh (LSM)

Figure 9. Lyngbye’s sedge marsh in the swale in between beach ridges at the mouth of Ikpek Lagoon, BELA, plot B-20180707-16. NPS photo, public domain.

**USNVC Group:**  Not classified.

**Plant association:** *Carex lyngbyaei* [sic] [5,6,11,12]

**Alaska Vegetation Class**: lll.A.3.i. Halophytic Sedge Wet Meadow

**ARCN Landcover Ecotype:** Not classified.

This near-monoculture is typically found in a narrow swale immediately adjacent to the outer beach ridges. A few other salt tolerant species were found in these plots, but none consistently. This type proved difficult to map due to its scarcity.

Table 17. Site variable summary for Lyngbye’s Sedge Marsh (LSM) (n=9).

|  | **Median** | **IQR** |
| --- | --- | --- |
| EC (µS/m) | 2100 | 3440 |
| Hydric index | 1.4 | 0.5 |
| Elevation (m) | 3.5 | 1.1 |
| Distance to ocean (km) | 0.4 | 0.3 |
| Distance to estuary (km) | 0.5 | 0.5 |
| *Functional group cover (%)* | | |
| Graminoid | 35 | 10 |
| Forb | 0 | 3 |
| Dwarf shrub | 0 | 0 |
| Low shrub | 0 | 0 |
| Tall shrub | 0 | 0 |
| Lichen | 0 | 0 |
| Moss | 0 | 0 |

Table 18. Species cover and frequency for Lyngbye’s Sedge Marsh (LSM) (n=9).

|  | Cover Class Frequency (%) | | | | | |  |
| --- | --- | --- | --- | --- | --- | --- | --- |
|  | 0:<1% | 1:1-5% | 2:6-25% | 3:26-50% | 4:51-75% | 5:76-100% | **Total Frequency (%)** |
| *Carex lyngbyei* | 0 | 0 | 0 | 22 | 56 | 22 | 100 |
| *Rumex arcticus* | 33 | 11 | 0 | 0 | 0 | 0 | 44 |
| *Comarum palustre* | 22 | 0 | 11 | 0 | 0 | 0 | 33 |
| *Caltha palustris* | 11 | 11 | 0 | 0 | 0 | 0 | 22 |
| *Cardamine pratensis* | 22 | 0 | 0 | 0 | 0 | 0 | 22 |
| *Carex rariflora* | 0 | 11 | 11 | 0 | 0 | 0 | 22 |
| *Hippuris tetraphylla* | 22 | 0 | 0 | 0 | 0 | 0 | 22 |
| Moss, other | 0 | 11 | 11 | 0 | 0 | 0 | 22 |
| *Andromeda polifolia* | 11 | 0 | 0 | 0 | 0 | 0 | 11 |
| *Betula nana* | 0 | 11 | 0 | 0 | 0 | 0 | 11 |
| *Carex aquatilis* | 0 | 11 | 0 | 0 | 0 | 0 | 11 |
| *Cladonia pyxidata* | 11 | 0 | 0 | 0 | 0 | 0 | 11 |
| *Cladonia* sp. | 0 | 11 | 0 | 0 | 0 | 0 | 11 |
| *Dupontia fisheri* | 11 | 0 | 0 | 0 | 0 | 0 | 11 |
| *Eriophorum angustifolium* | 0 | 11 | 0 | 0 | 0 | 0 | 11 |
| *Iris setosa* | 11 | 0 | 0 | 0 | 0 | 0 | 11 |
| *Juncus arcticus* | 0 | 11 | 0 | 0 | 0 | 0 | 11 |
| *Juncus castaneus* | 11 | 0 | 0 | 0 | 0 | 0 | 11 |
| *Pinguicula vulgaris* | 11 | 0 | 0 | 0 | 0 | 0 | 11 |
| *Poa* sp. | 11 | 0 | 0 | 0 | 0 | 0 | 11 |
| *Ranunculus* sp. | 11 | 0 | 0 | 0 | 0 | 0 | 11 |
| *Salix arctica* | 11 | 0 | 0 | 0 | 0 | 0 | 11 |
| *Salix fuscescens* | 11 | 0 | 0 | 0 | 0 | 0 | 11 |
| *Salix pulchra* | 0 | 0 | 11 | 0 | 0 | 0 | 11 |
| *Saxifraga hirculus* | 11 | 0 | 0 | 0 | 0 | 0 | 11 |
| *Vaccinium uliginosum* | 0 | 11 | 0 | 0 | 0 | 0 | 11 |

## Pendantgrass lagoon margin (PLM)

Figure 10. A narrow swath of pendantgrass at plot A-20190705-07, inner Cowpack Lagoon, BELA. NPS photo, public domain.

**USNVC Group:** G370 *Arctophila fulva - Carex aquatilis* Arctic Marsh Group

**Plant associations:** Several *Arctophila fulva* monoculture types in freshwater habitats have been previously published (see Viereck et al. 1992). We do not describe a brackish type here, as our sample is ecotonal.

**Alaska Vegetation Class**: Not classified.

**ARCN Landcover Ecotype:** Not classified.

We include several overlapping, transitional plant associations in this class. It is defined as emergent or wet vegetation found immediately along the margin of inner lagoons that is dominated by *Arctophila fulva* or other tall sedges and grasses. It is most widespread in BELA. This habitat is a typically only 2-3 m wide. Almost all plots were ecotonal, and likely highly variable with seasonal snowmelt as well as storm surge. In several sites we visited in early July in 2019, the plots were phenologically far behind the rest of the vegetation, presumably due to late-melting snow.

The predominant species, *Arctophila fulva* (Pendantgrass), is an emergent grass, commonly found in pure stands in freshwater ponds and slow-moving streams. This class is a spectrum, from plots that are near-monocultures of *Arctophila fulva*, to plots that contain a variety of other salt-tolerant, emergent species. The widespread, hydrophilic *Eriophorum angustifolium* (tall cottonsedge) and *Equisetum arvense* (field horsetail) were frequently co-dominant. We include two plots where those species are dominant over *Arctophila fulva* (A-20190705-08 and A-20190705-11), as they occurred in the same habitat and had a similar suite of co-occurring species.

Table 19. Site variable summary for Pendantgrass Lagoon Margin (PM) (n=15).

|  | **Median** | **IQR** |
| --- | --- | --- |
| EC (µS/m) | 598 | 1327 |
| Hydric index | 1.6 | 0.6 |
| Elevation (m) | 3.8 | 0.5 |
| Distance to ocean (km) | 5.4 | 3.5 |
| Distance to estuary (km) | 0 | 0 |
| *Functional group cover (%)* | | |
| Graminoid | 35 | 37.5 |
| Forb | 5 | 18 |
| Dwarf shrub | 0 | 0 |
| Low shrub | 0 | 0 |
| Tall shrub | 0 | 0 |
| Lichen | 0 | 0 |
| Moss | 0 | 0 |

Table 20. Species cover and frequency for Pendantgrass Lagoon Margin (PLM) (n=15).

|  | Cover Class Frequency (%) | | | | | |  |
| --- | --- | --- | --- | --- | --- | --- | --- |
|  | 0:<1% | 1:1-5% | 2:6-25% | 3:26-50% | 4:51-75% | 5:76-100% | **Total Frequency (%)** |
| *Arctophila fulva* | 7 | 7 | 20 | 47 | 7 | 7 | 93 |
| *Potentilla egedii* | 47 | 33 | 0 | 0 | 0 | 0 | 80 |
| *Rumex arcticus* | 47 | 7 | 0 | 0 | 0 | 0 | 53 |
| *Equisetum arvense* | 7 | 20 | 13 | 7 | 0 | 0 | 47 |
| *Eriophorum angustifolium* | 13 | 7 | 13 | 13 | 0 | 0 | 47 |
| *Leymus mollis* | 13 | 20 | 7 | 0 | 0 | 0 | 40 |
| *Chrysanthemum arcticum* | 33 | 0 | 0 | 0 | 0 | 0 | 33 |
| *Puccinellia phryganodes* | 7 | 27 | 0 | 0 | 0 | 0 | 33 |
| *Stellaria humifusa* | 7 | 20 | 7 | 0 | 0 | 0 | 33 |
| *Calamagrostis canadensis* | 0 | 13 | 13 | 0 | 0 | 0 | 27 |
| *Cardamine pratensis* | 20 | 7 | 0 | 0 | 0 | 0 | 27 |
| *Carex lyngbyei* | 7 | 13 | 0 | 7 | 0 | 0 | 27 |
| *Petasites frigidus* | 13 | 13 | 0 | 0 | 0 | 0 | 27 |
| *Polemonium acutiflorum* | 20 | 7 | 0 | 0 | 0 | 0 | 27 |
| *Artemisia tilesii* | 20 | 0 | 0 | 0 | 0 | 0 | 20 |
| *Comarum palustre* | 13 | 7 | 0 | 0 | 0 | 0 | 20 |
| *Poa eminens* | 0 | 7 | 13 | 0 | 0 | 0 | 20 |
| *Angelica lucida* | 13 | 0 | 0 | 0 | 0 | 0 | 13 |
| *Atriplex gmelinii* | 7 | 7 | 0 | 0 | 0 | 0 | 13 |
| *Carex aquatilis* | 7 | 0 | 7 | 0 | 0 | 0 | 13 |
| *Carex glareosa* | 7 | 7 | 0 | 0 | 0 | 0 | 13 |
| *Carex rariflora* | 0 | 13 | 0 | 0 | 0 | 0 | 13 |
| *Carex subspathacea* | 0 | 13 | 0 | 0 | 0 | 0 | 13 |
| *Hippuris tetraphylla* | 7 | 7 | 0 | 0 | 0 | 0 | 13 |
| *Rhodiola integrifolia* | 13 | 0 | 0 | 0 | 0 | 0 | 13 |
| *Spiraea stevenii* | 13 | 0 | 0 | 0 | 0 | 0 | 13 |
| *Stellaria* sp. | 7 | 7 | 0 | 0 | 0 | 0 | 13 |

## Rock sedge marsh (RSM)

Figure 11. Plot A-20180718-16, Cake Krusenstern, CAKR. NPS photo, public domain.

**USNVC Group:**  Not classified.

**Plant association:** *Carex saxatilis/Salix pulchra/Iris setosa*

**Alaska Vegetation Class:** lll.A.3.i. Halophytic Sedge Wet Meadow

**ARCN Landcover Ecotype:** Not classified.

This type is found solely on Cape Krusenstern in the narrow swales several beach ridges from the ocean. Other species include *Iris setosa* and *Salix pulchra*.

Table 21. Site variable summary for Rock Sedge Marsh (RSM) (n=3).

|  | **Median** | **IQR** |
| --- | --- | --- |
| EC (µS/m) | 292 | 120 |
| Hydric index | 2.2 | 0.1 |
| Elevation (m) | 4.4 | 0.2 |
| Distance to ocean (km) | 0.5 | 0.1 |
| Distance to estuary (km) | 1.6 | 0.1 |
| *Functional group cover (%)* | | |
| Graminoid | 20 | 7.5 |
| Forb | 3 | 1 |
| Dwarf shrub | 1 | 1 |
| Low shrub | 15 | 17.5 |
| Tall shrub | 0 | 0 |
| Lichen | 0 | 0 |
| Moss | 0 | 0 |

Table 22. Species cover and frequency for Rock Sedge Marsh (RSM) (n=3).

|  | Cover Class Frequency (%) | | | |  |
| --- | --- | --- | --- | --- | --- |
|  | 0:<1% | 1:1-5% | 2:6-25% | 3:26-50% | **Total Frequency (%)** |
| *Carex saxatilis* | 0 | 0 | 33 | 67 | 100 |
| *Iris setosa* | 67 | 33 | 0 | 0 | 100 |
| *Salix pulchra* | 33 | 33 | 33 | 0 | 100 |
| *Andromeda polifolia* | 67 | 0 | 0 | 0 | 67 |
| *Betula nana* | 67 | 0 | 0 | 0 | 67 |
| *Carex aquatilis* | 0 | 33 | 33 | 0 | 67 |
| *Rubus chamaemorus* | 67 | 0 | 0 | 0 | 67 |
| *Salix richardsonii* | 33 | 33 | 0 | 0 | 67 |
| *Vaccinium uliginosum* | 33 | 33 | 0 | 0 | 67 |
| *Aulacomnium palustre/acuminatum* | 0 | 33 | 0 | 0 | 33 |
| *Aulacomnium turgidum* | 33 | 0 | 0 | 0 | 33 |
| *Cardamine bellidifolia* | 33 | 0 | 0 | 0 | 33 |
| *Empetrum hermaphroditum* | 33 | 0 | 0 | 0 | 33 |
| *Equisetum arvense* | 0 | 0 | 33 | 0 | 33 |
| Moss, other | 0 | 33 | 0 | 0 | 33 |
| *Saxifraga hirculus* | 33 | 0 | 0 | 0 | 33 |

## Salt marsh (SM)

Figure 12a. Typical *Carex subspathacea* dominated salt marsh-water mosaic at the mouth of the Nugnugaluktuk Estuary. 12b. *Puccinellia phryganodes* dominated salt marsh, heavily grazed by geese. NPS photos, public domain.

**USNVC Group:** G535 *Carex subspathacea - Dupontia fisheri* Arctic Coastal Salt Marsh Group (USNVC 2019)

**Plant associations:** *Carex subspathacea* [5,8,10,13]; *Carex subspathacea-Puccinellia phryganodes* [6,14]; *Carex subspathacea-Salix ovalifolia* [10]*; Puccinellia phryganodes* [6,10,13]

**Alaska Vegetation Class****:** III.A.3.i - Halophytic wet sedge meadow; III.A.3.h Halophytic wet grass meadow

**ARCN Landcover Ecotype:** Coastal Saline Sedge–Grass Meadow; Coastal Brackish Sedge–Grass Meadow

This vegetation type is found in low-lying areas saturated with seawater, but protected from direct wave action. This allows the vegetation to build up a deep layer of organic muck. Typical along inlets, estuary mouths and the inner beach of lagoons, this widespread type was nearly indistinguishable in CAKR and BELA.

Dominated by *Carex subspathacea* and *Puccinellia phryganodes*, which are frequently monocultures. Only a handful of other species can tolerate the high salinity of this habitat. This highly productive type is vital migratory bird habitat.

Table 23. Site variable summary for Salt Marsh (SM) (n=128).

|  | **Median** | **IQR** |
| --- | --- | --- |
| EC (µS/m) | 4000 | 0 |
| Hydric index | 1.5 | 0.6 |
| Elevation (m) | 4 | 1.5 |
| Distance to ocean (km) | 2 | 6.7 |
| Distance to estuary (km) | 0.3 | 1.3 |
| *Functional group cover (%)* | | |
| Graminoid | 45 | 26 |
| Forb | 6 | 16 |
| Dwarf shrub | 0 | 0 |
| Low shrub | 0 | 0 |
| Tall shrub | 0 | 0 |
| Lichen | 0 | 0 |
| Moss | 0 | 0 |

Table 24. Species cover and frequency for Salt Marsh (SM) (n=128).

|  | Cover Class Frequency (%) | | | | | |  |
| --- | --- | --- | --- | --- | --- | --- | --- |
|  | 0:<1% | 1:1-5% | 2:6-25% | 3:26-50% | 4:51-75% | 5:76-100% | **Total Frequency (%)** |
| *Carex subspathacea* | 1 | 14 | 26 | 24 | 14 | 5 | 84 |
| *Potentilla egedii* | 18 | 32 | 13 | 2 | 1 | 0 | 65 |
| *Chrysanthemum arcticum* | 20 | 32 | 10 | 0 | 0 | 0 | 62 |
| *Puccinellia phryganodes* | 3 | 12 | 26 | 9 | 5 | 2 | 58 |
| *Leymus mollis* | 16 | 15 | 17 | 5 | 0 | 0 | 52 |
| *Stellaria humifusa* | 27 | 20 | 3 | 0 | 0 | 0 | 50 |
| *Calamagrostis deschampsioides* | 8 | 16 | 8 | 2 | 0 | 0 | 34 |
| *Carex glareosa* | 6 | 8 | 8 | 2 | 0 | 0 | 24 |
| *Salix ovalifolia* | 5 | 5 | 8 | 2 | 1 | 0 | 20 |
| *Saussurea nuda* | 8 | 6 | 2 | 0 | 0 | 0 | 17 |
| *Cochlearia officinalis* | 9 | 2 | 0 | 0 | 0 | 0 | 11 |
| *Rumex arcticus* | 10 | 2 | 0 | 0 | 0 | 0 | 11 |

## Tall shrub upland (TSU)

This type was only incidentally sampled in our vegetation scheme, as it is not under tidal influence. We include it in the map as it frequently demarcates the abrupt transition from coast to upland, and the transition from brackish estuary systems to fresh riverine habitat.

## Upland tundra (UT)

This is a heterogeneous type, predominately composed of low shrub and graminoid dominated polygonal tundra. It includes all vegetation beyond the influence of potential storm surge, and marks the boundary of our interest in coastal mapping.

## Unclassified plots

A total of 9 plots collected for this project, and 3 collected for the earlier ARCN landcover classification, were unable to be assigned to vegetation classes. The data for those plots, designated as ‘UNC’, can found in an Access database and flat files available in the IRMA Data Store reference for this project, <https://irma.nps.gov/DataStore/Reference/Profile/2272456>.

# Citations

1. Jorgenson MT, Roth JE, Miller PF, Macander MJ, Duffy MS, Wells AF, et al. An ecological land survey and landcover map of the Arctic Network. Fort Collins (CO): National Park Service; 2009. Report No.: ARCN/NRTR-2009/270. Available: https://irma.nps.gov/App/Reference/Profile/663934

2. Viereck LA, Dyrness CT, Batten AR, Wenzlick KJ. The Alaska vegetation classification. Pacific Northwest Research Station: USDA Forest Service; 1992. Report No.: PNW-GTR-286.

3. Parker CL. Vascular Plant Inventory of Alaska‘s Arctic National Parklands. Fort Collins, Colorado: National Park Service; 2006. Report No.: NPS/AKRARCN/NRTR-2006/01.

4. Potter LD. Plant Ecology of the Walakpa Bay Area, Alaska. ARCTIC. 1972;25: 115–130. doi:10.14430/arctic2951

5. Hanson HC. Characteristics of Some Grassland, Marsh, and Other Plant Communities in Western Alaska. Ecol Monogr. 1951;21: 317–378. doi:10.2307/1948654

6. Kincheloe KL, Stehn RA. Vegetation patterns and environmental gradients in coastal meadows on the Yukon–Kuskokwim delta, Alaska. Can J Bot. 1991;69: 1616–1627. doi:10.1139/b91-205

7. Batten AR. The vascular floristics, major vegetation units, and phytogeography of the Lake Peters area, northeastern Alaska. M.S. Thesis, University of Alaska, Fairbanks. 1977.

8. Hanson HC. Vegetation Types in Northwestern Alaska and Comparisons with Communities in Other Arctic Regions. Ecology. 1953;34: 111–140. doi:10.2307/1930313

9. Shacklette H, Durrell LW, Krog H, Erdman JA, Persson H, Keith JR, et al. Vegetation of Amchitka Island, Aleutian Islands, Alaska. Washington, D.C.: US Geological Survey; 1969. Report No.: 648.

10. Boggs K, Boucher TV, McTeague ML. Plant Association Classification for Northern Alaska. Alaska Natural Heritage Program, Alaska Center for Conservation Science, University of Alaska Anchorage; 2018 p. 144.

11. Vince SW, Snow AA. Plant Zonation in an Alaskan Salt Marsh: I. Distribution, Abundance and Environmental Factors. J Ecol. 1984;72: 651. doi:10.2307/2260074

12. del Moral R, Watson AF. Vegetation on the Stikine Flats, southeast Alaska. Northwest Sci. 1978;52.

13. Meyers C. Vegetation of the Beaufort Sea coast, Alaska: community composition, distribution, and tidal influences. M.S. Thesis, University of Alaska, Fairbanks. 1985.

14. Byrd GV, Ronsse D. Preliminary classification of plant communities in the vegetated intertidal zone of the central Yukon Delta, Alaska. Bethel, Alaska: US Fish and Wildlife Service; 1983.
