# Supplementary figures and images for: A high-resolution map of coastal vegetation for two Arctic Alaskan parklands: An object-oriented approach with point training data
Source: PLoS One. 2022 Aug 31;17(8):e0273893. doi: 10.1371/journal.pone.0273893 (PMC9432696; doi:10.1371/journal.pone.0273893)

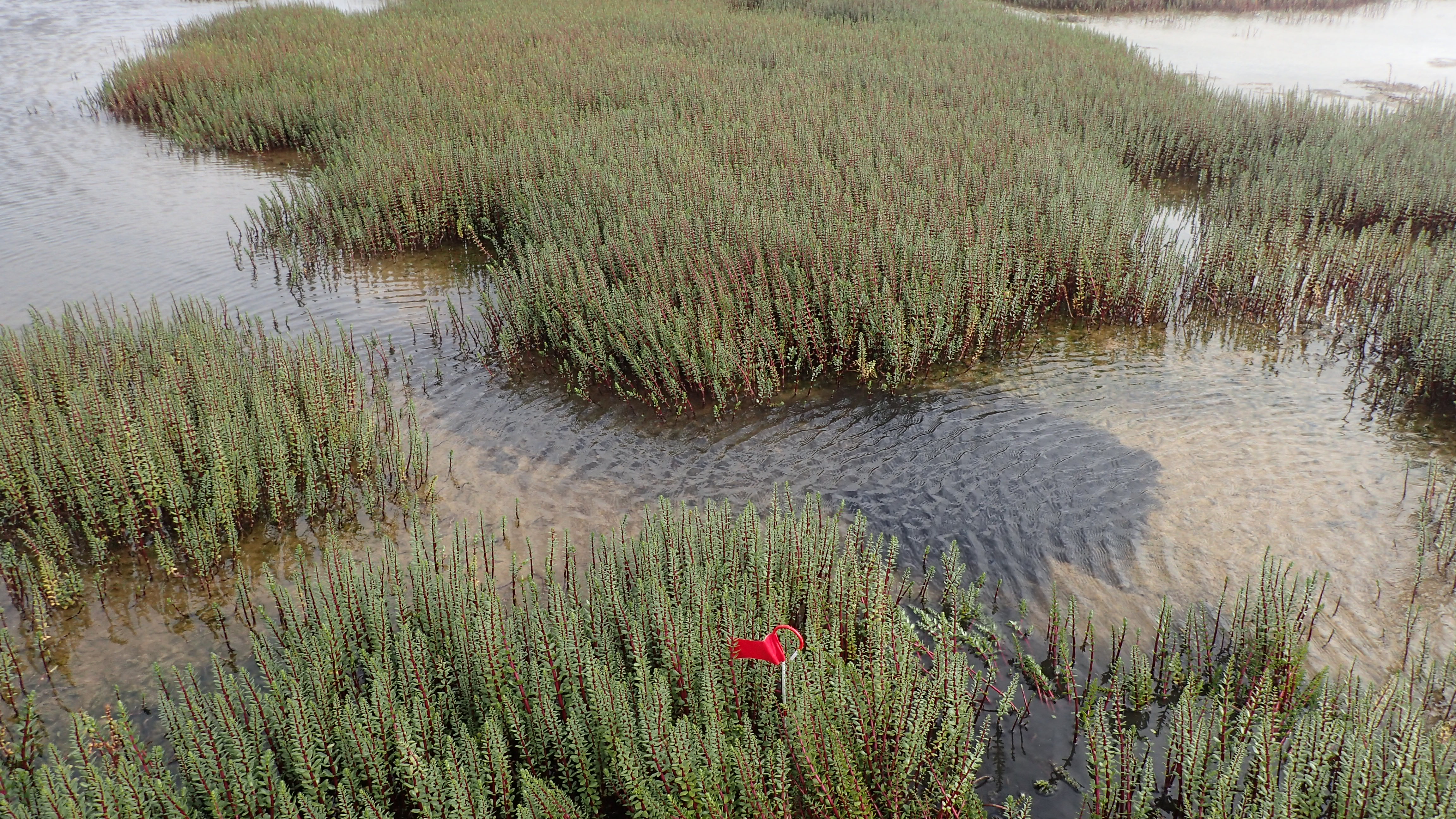

Supplement: S1 File — (ZIP) [file pone.0273893.s008.zip › Appendix1_Vegetation_types/FigA1_BMM_P7160897_A_2018.JPG]

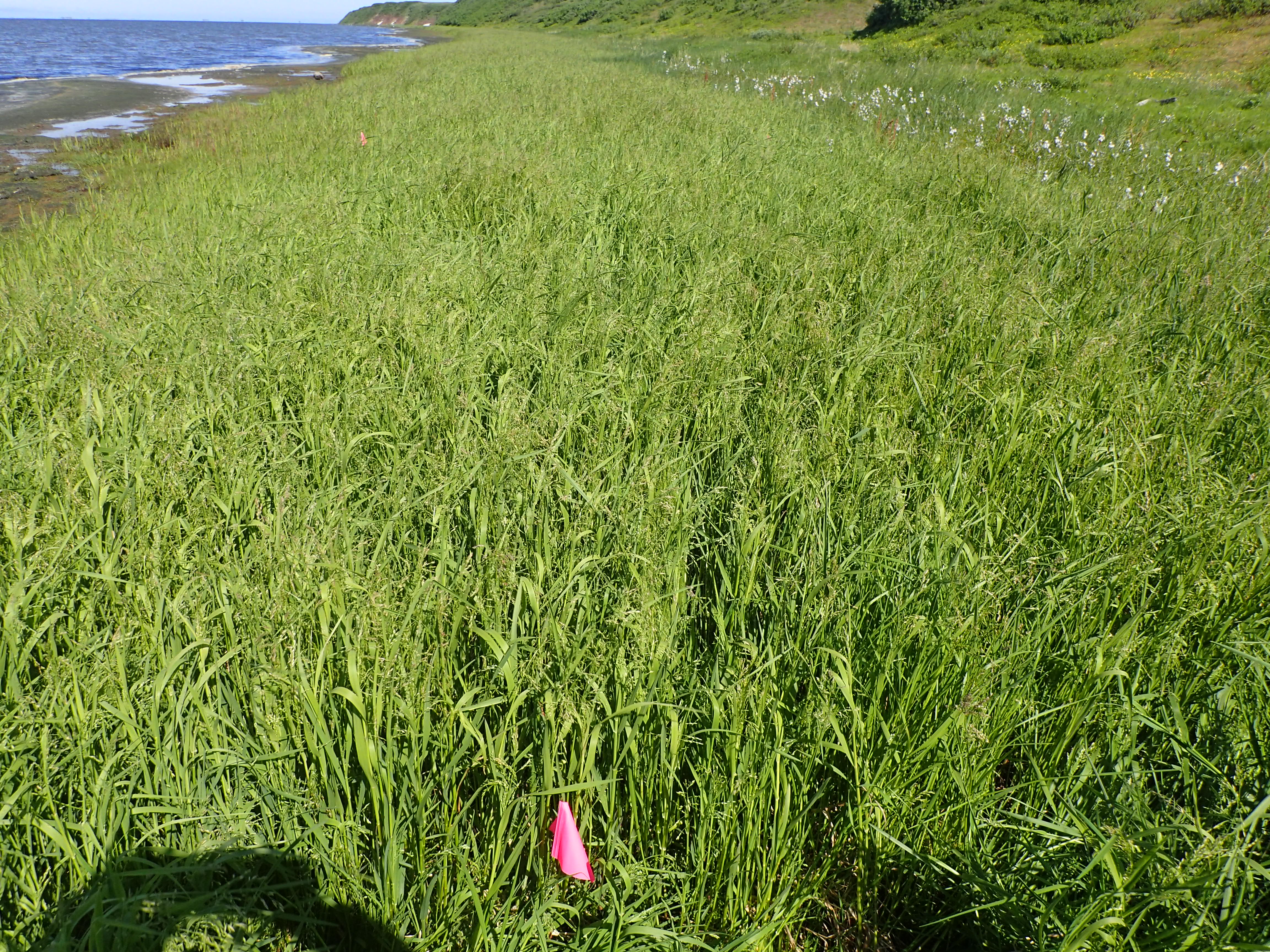

Supplement: S1 File — (ZIP) [file pone.0273893.s008.zip › Appendix1_Vegetation_types/FigA10_PLM_P7050313_A_2019.JPG]

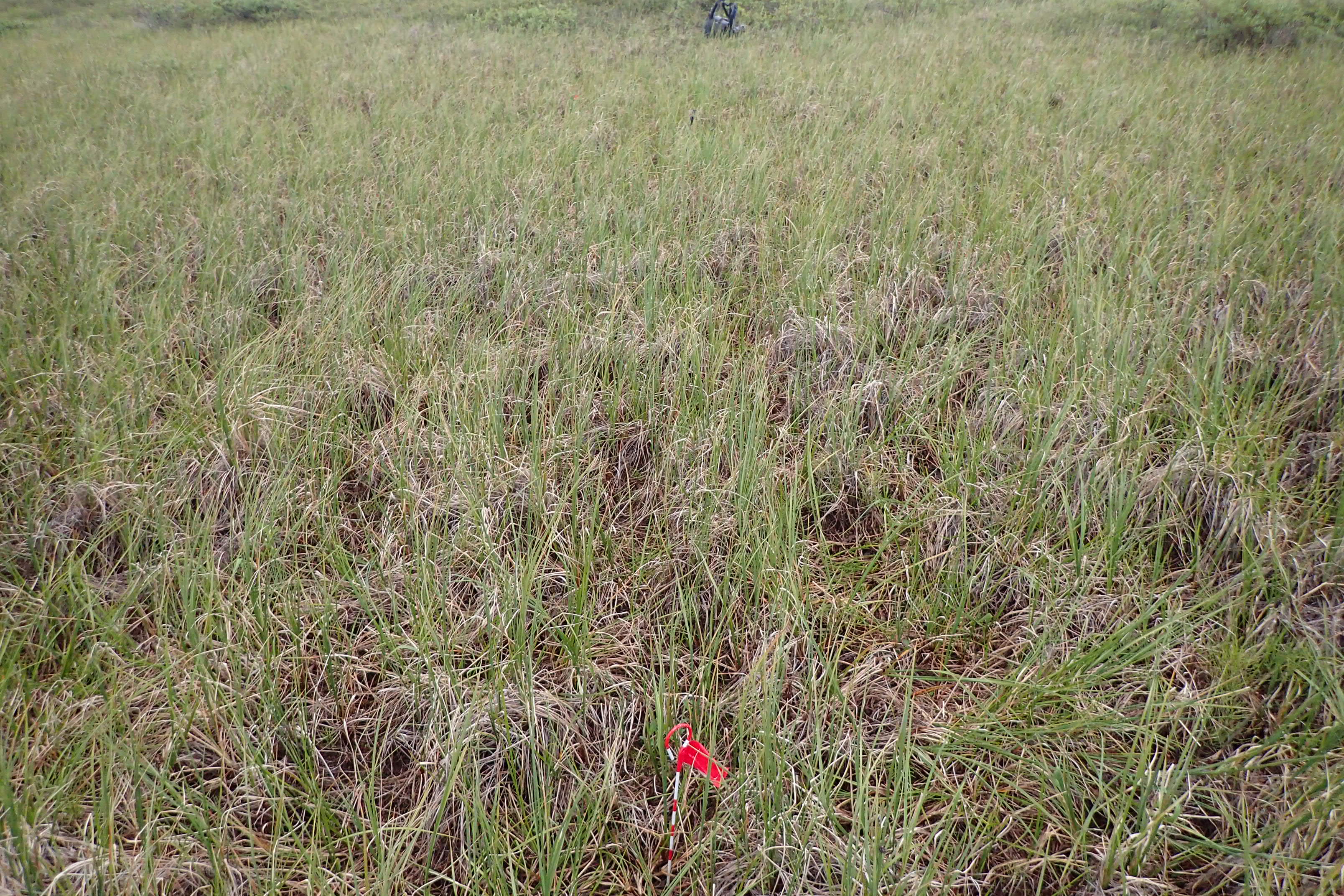

Supplement: S1 File — (ZIP) [file pone.0273893.s008.zip › Appendix1_Vegetation_types/FigA11_RSM_P7181141_A_2018.JPG]

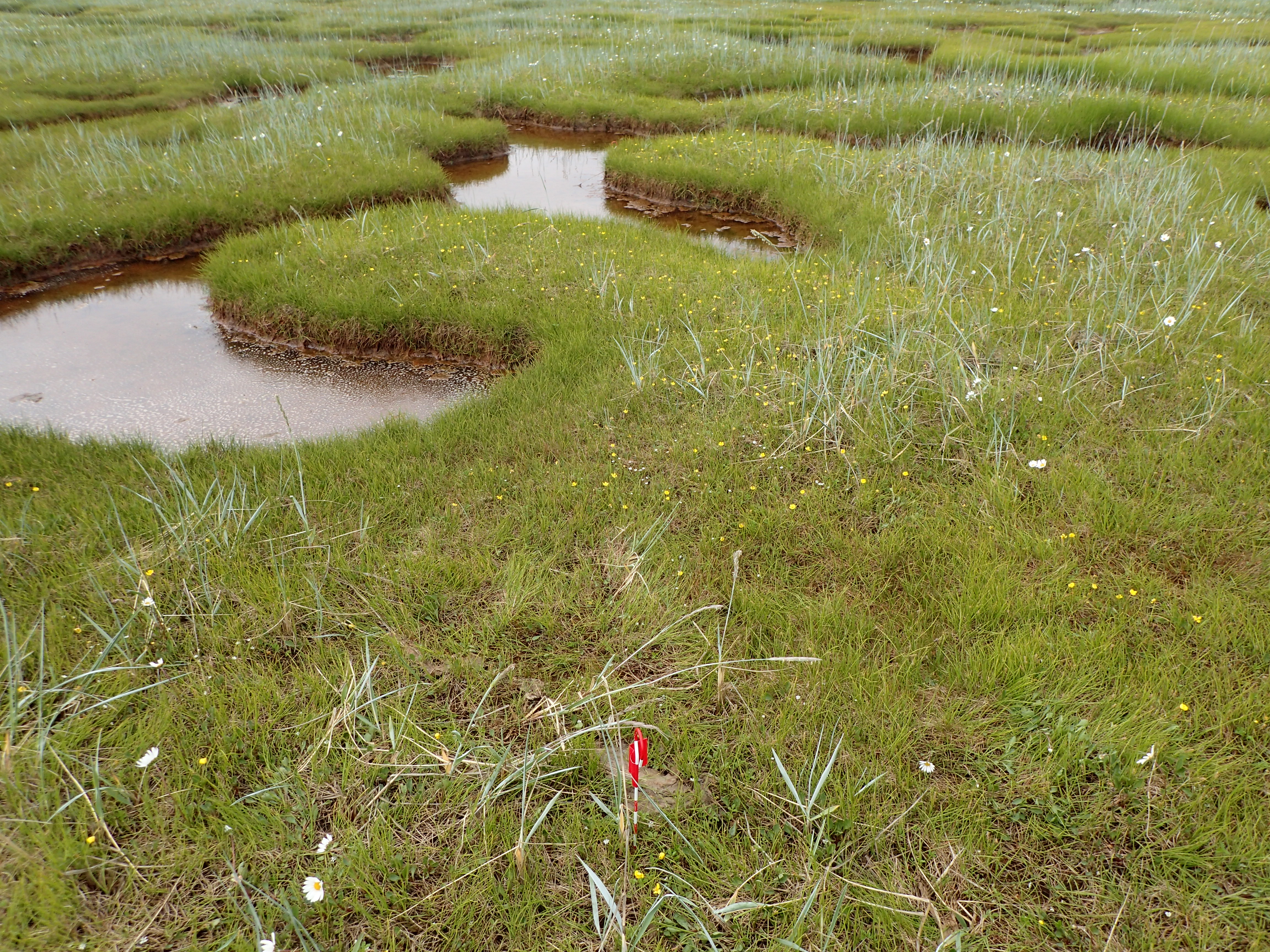

Supplement: S1 File — (ZIP) [file pone.0273893.s008.zip › Appendix1_Vegetation_types/FigA12_a_SM_P7050272_A_2018.JPG]

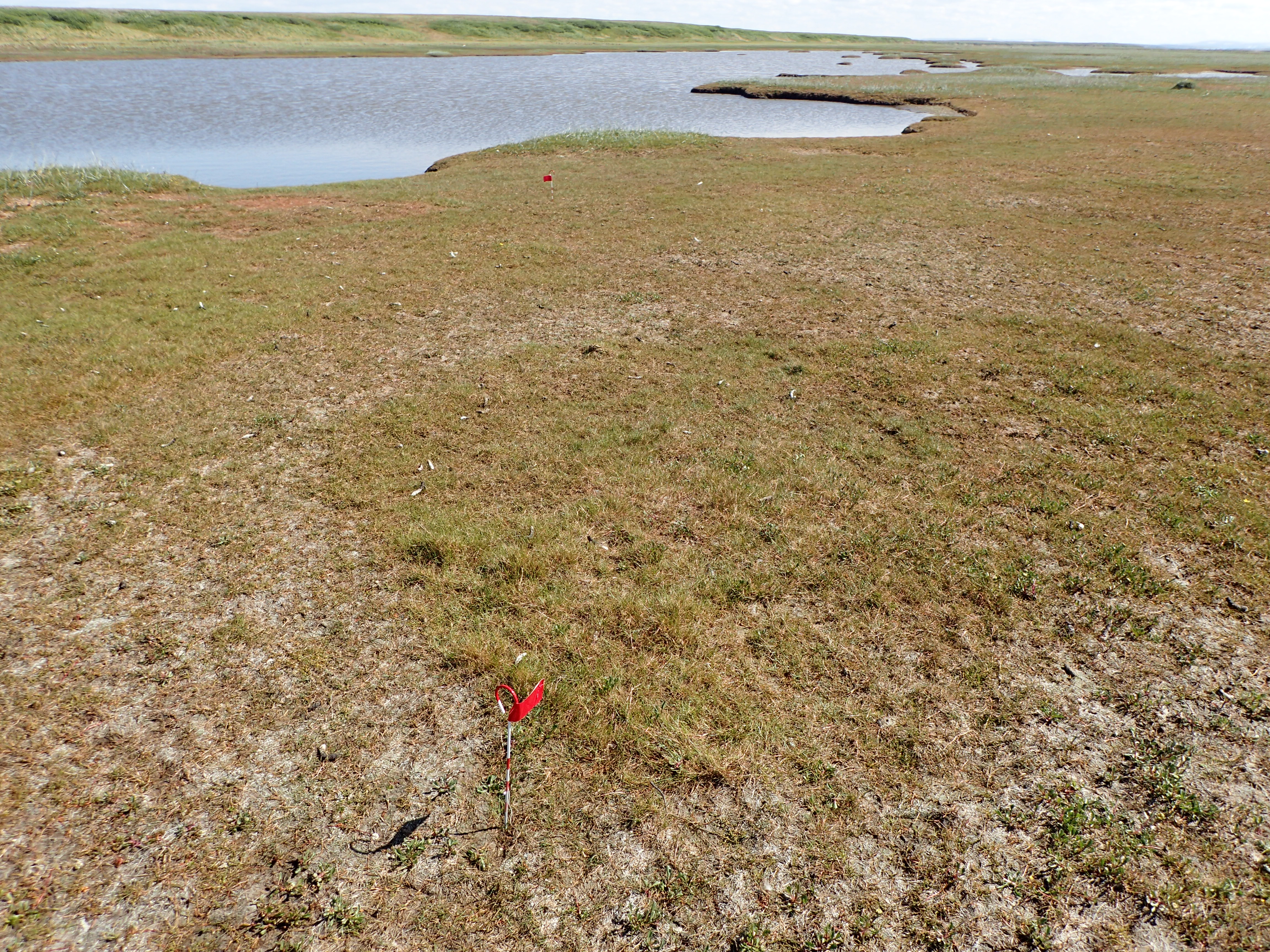

Supplement: S1 File — (ZIP) [file pone.0273893.s008.zip › Appendix1_Vegetation_types/FigA12_b_SM_P7070417_A_2018.JPG]

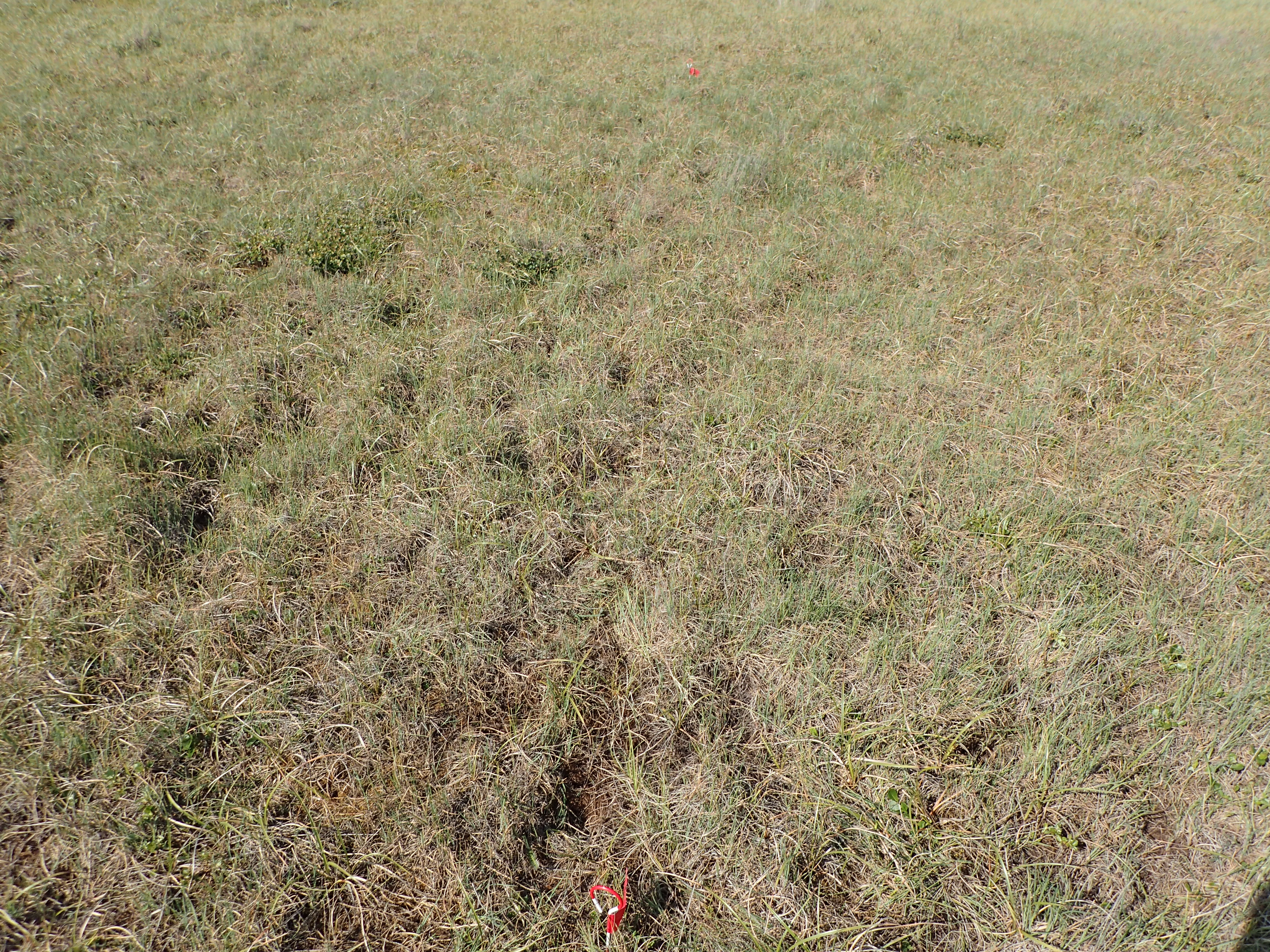

Supplement: S1 File — (ZIP) [file pone.0273893.s008.zip › Appendix1_Vegetation_types/FigA2_BSWM_P7060325_B_2018.JPG]

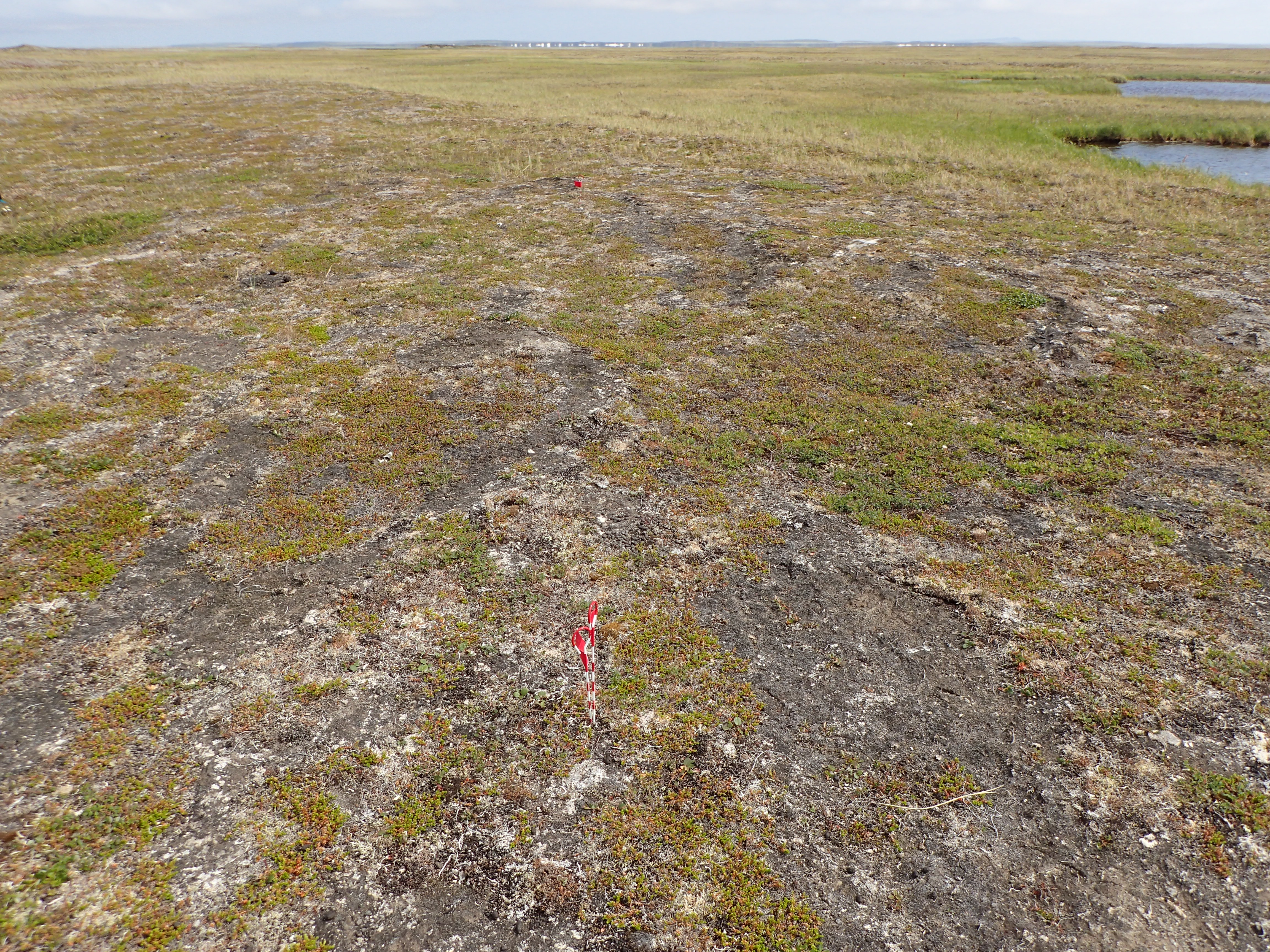

Supplement: S1 File — (ZIP) [file pone.0273893.s008.zip › Appendix1_Vegetation_types/FigA3_CLT_P7040169_A_2018.JPG]

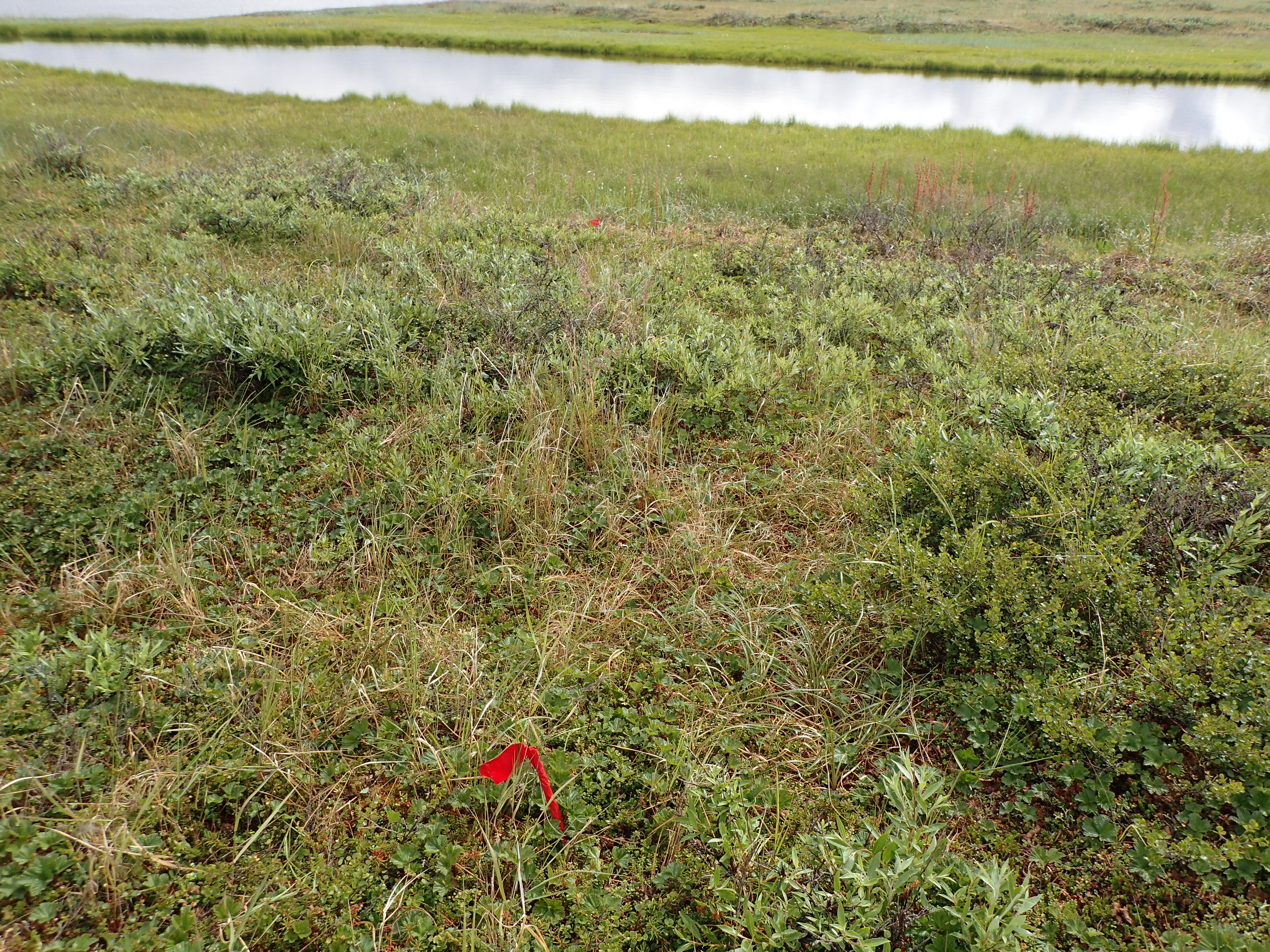

Supplement: S1 File — (ZIP) [file pone.0273893.s008.zip › Appendix1_Vegetation_types/FigA4_DLST_P7100541_A_2018.JPG]

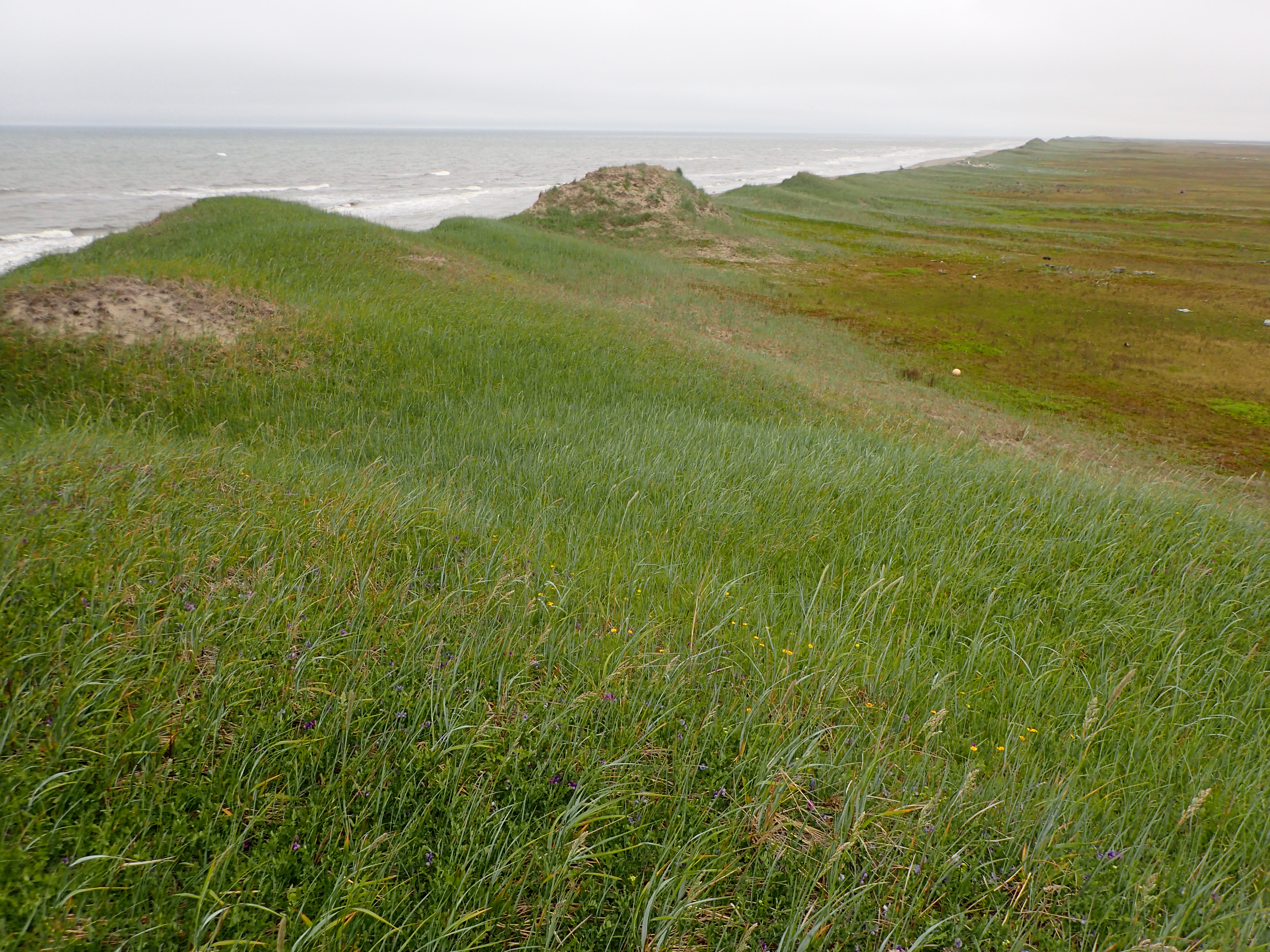

Supplement: S1 File — (ZIP) [file pone.0273893.s008.zip › Appendix1_Vegetation_types/FigA5_DBM_P7040179_A_2018.JPG]

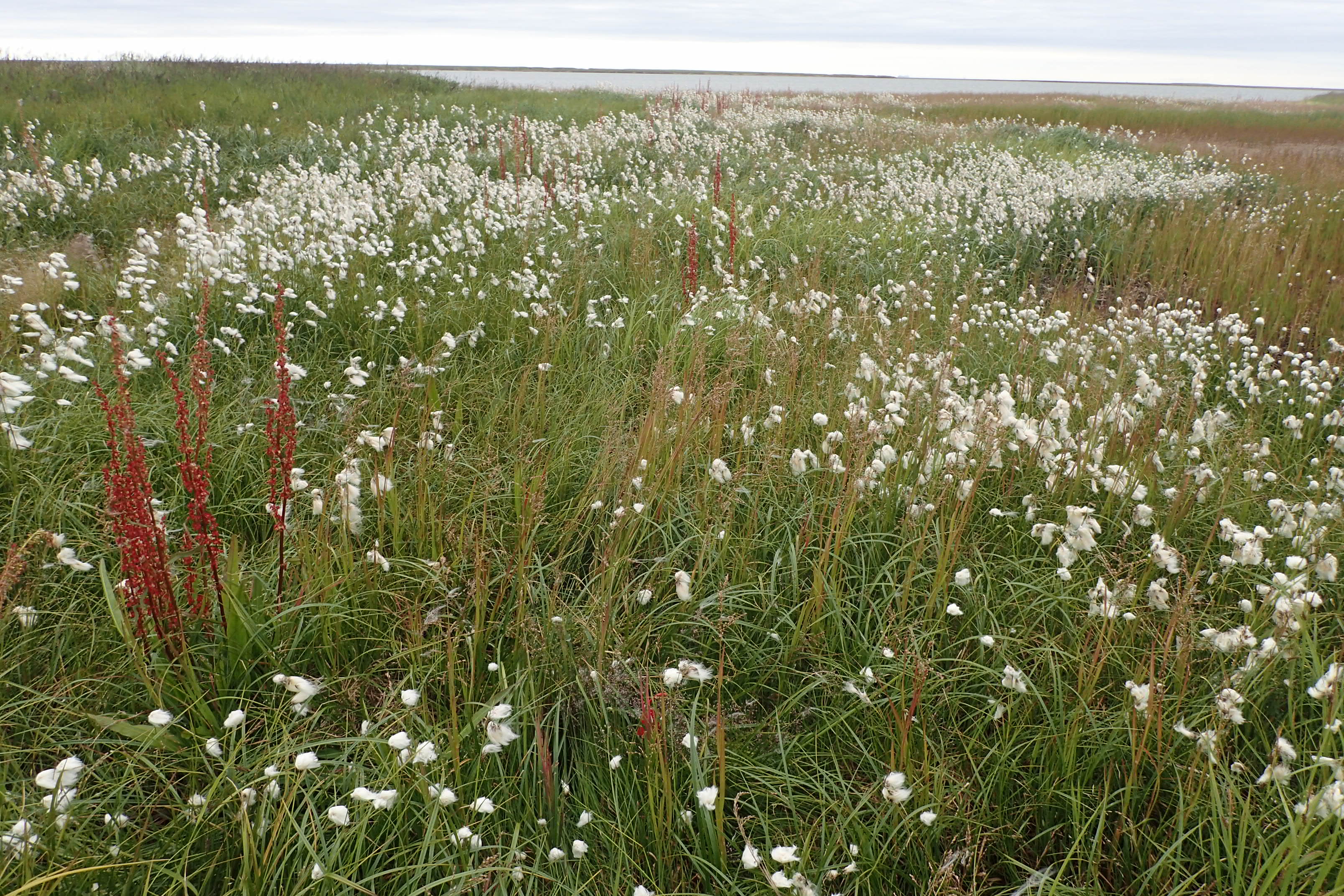

Supplement: S1 File — (ZIP) [file pone.0273893.s008.zip › Appendix1_Vegetation_types/FigA6_FBSWM_P7170973_A_2018.JPG]

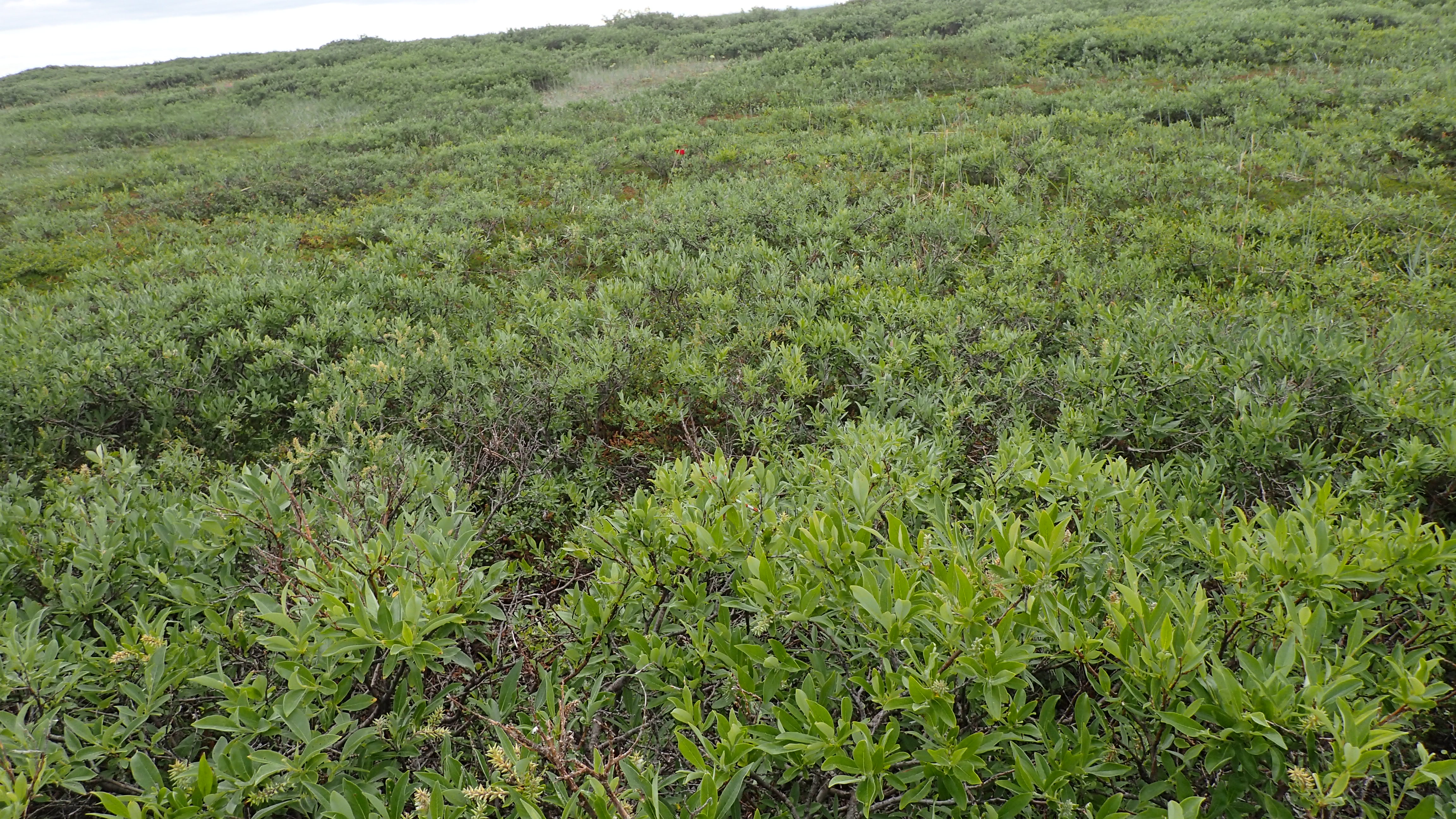

Supplement: S1 File — (ZIP) [file pone.0273893.s008.zip › Appendix1_Vegetation_types/FigA7_GWS_P7140701_A_2018.JPG]

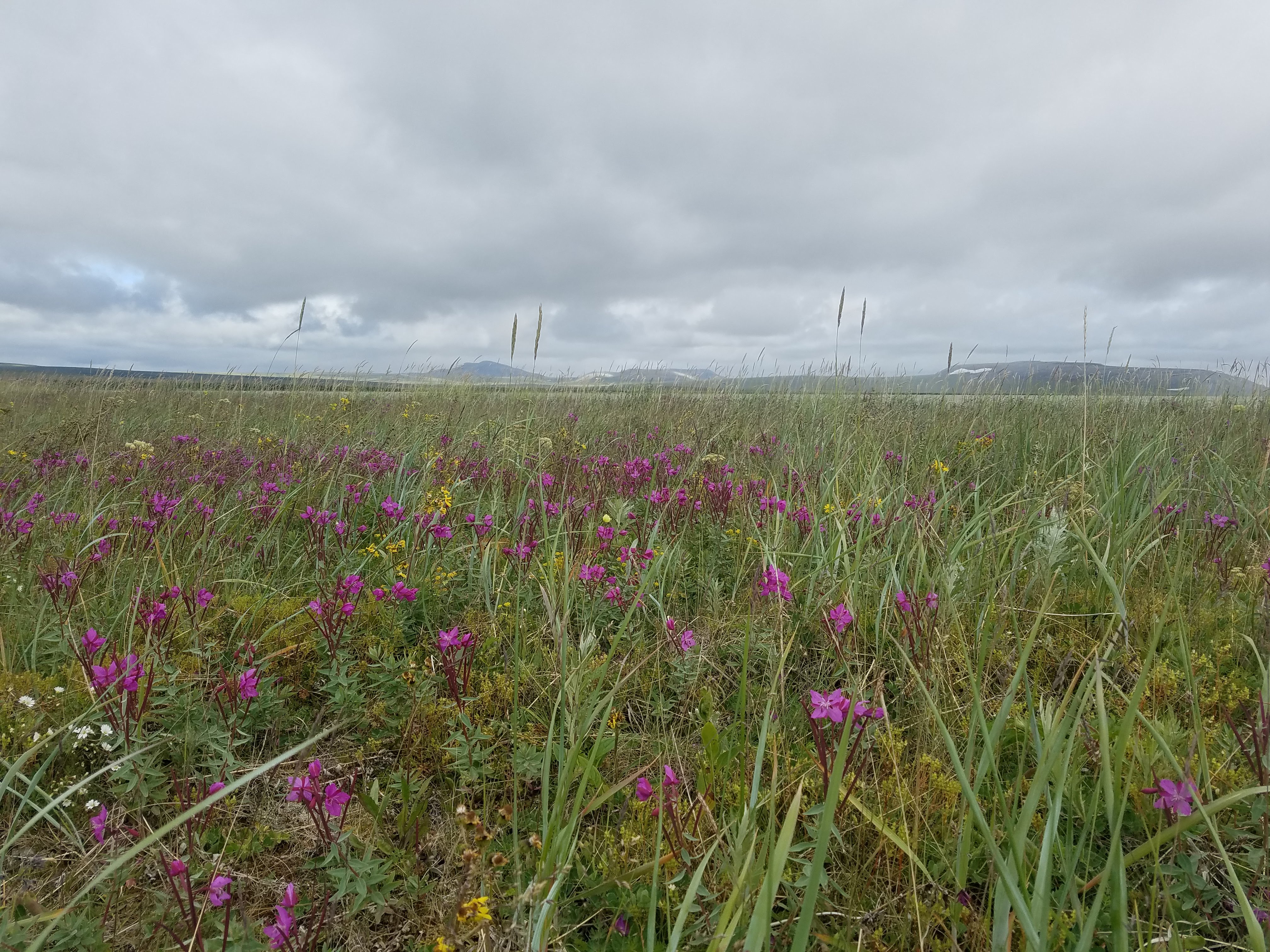

Supplement: S1 File — (ZIP) [file pone.0273893.s008.zip › Appendix1_Vegetation_types/FigA8_HDRBM_20180716_172808.jpg]

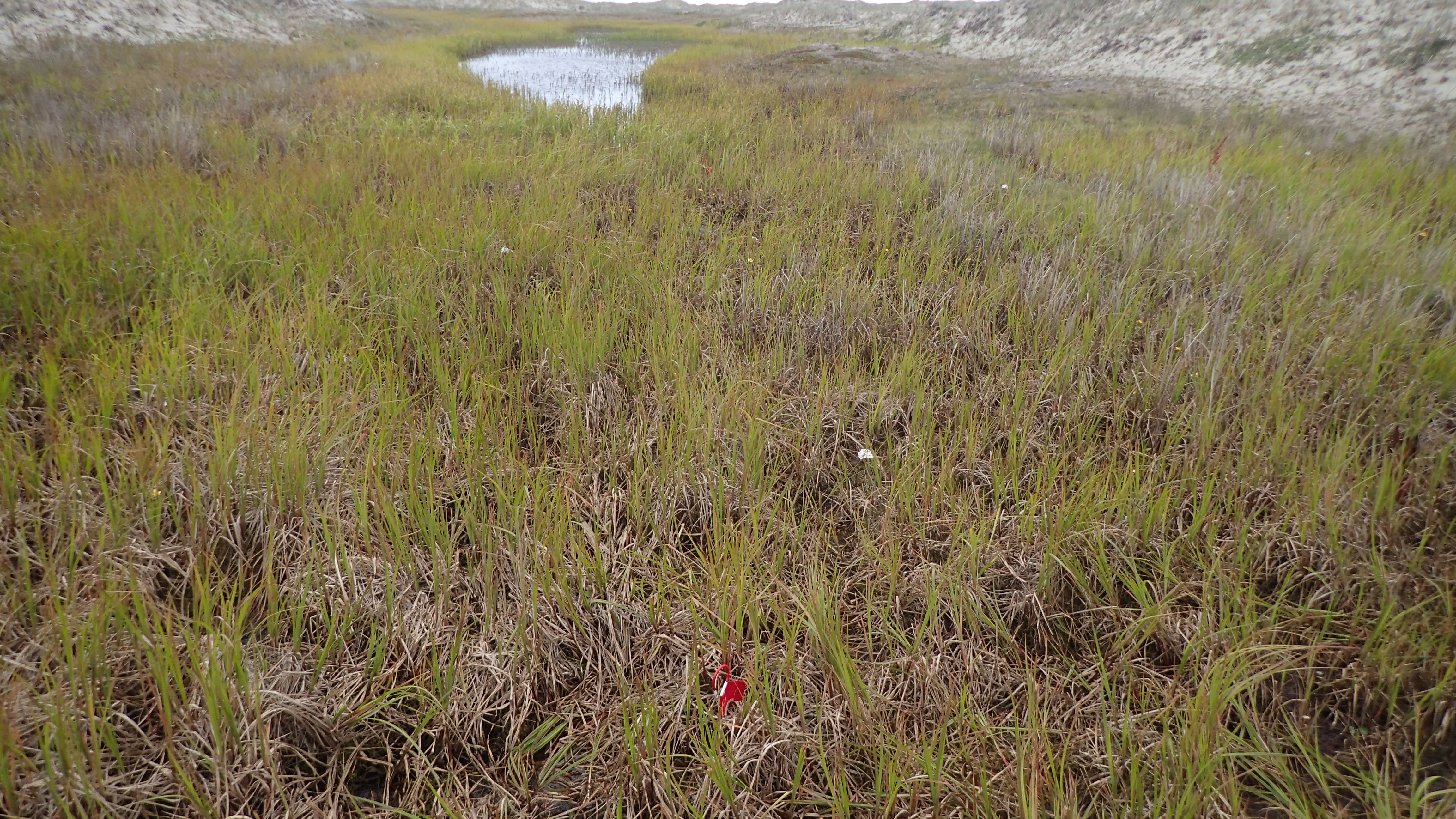

Supplement: S1 File — (ZIP) [file pone.0273893.s008.zip › Appendix1_Vegetation_types/FigA9_LSM_P7070382_B_2018.JPG]
