# Supplementary material for: A high-resolution map of coastal vegetation for two Arctic Alaskan parklands: An object-oriented approach with point training data
Source: PLoS One. 2022 Aug 31;17(8):e0273893. doi: 10.1371/journal.pone.0273893 (PMC9432696; doi:10.1371/journal.pone.0273893)
Supplement: S2 File — (DOCX) [file pone.0273893.s009.docx]

# Workflow Protocol

Code and supporting file structure available from https://irma.nps.gov/DataStore/Reference/Profile/2272456


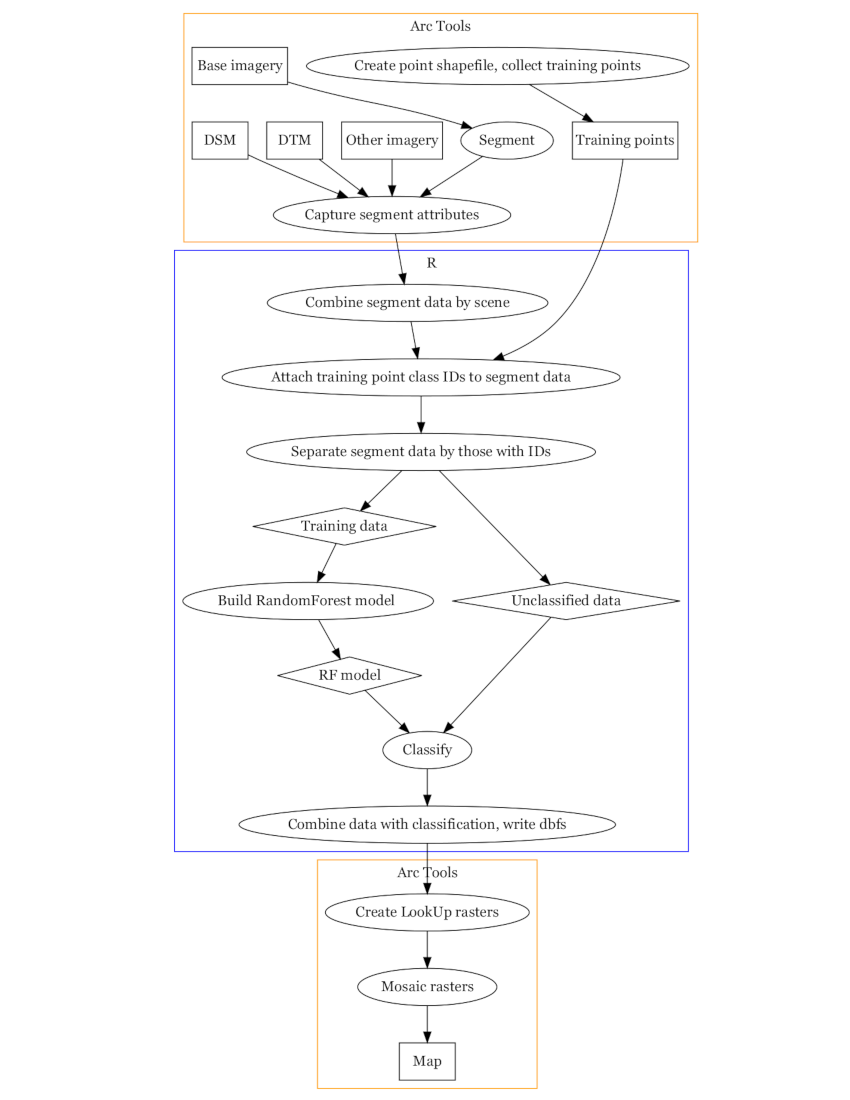


**Fig A13. Workflow diagram for the CAKR/BELA object-oriented map.** Boxes represent tools or processes, ellipses represent geospatial objects, diamonds are R objects.

The first steps should be taken manually, in the GIS software of your choice.

1. Identify your base imagery and auxiliary imagery.
   1. We aimed to have high resolution imagery with low cloud cover that was available for whole study area. Worldview2 imagery filled this niche, with a small gap supplemented by IKONOS.
   2. For our study area we derived attributes from the 5-meter resolution IFSAR DEM available for the state of Alaska. We cropped the DTM and DSM from this data set to our study area, and then derived slope, aspect and curvature maps in ArcMap tools. We also captured spectral data from aerial photography mosaics (see Methods). These data do not need to be the same resolution as the base imagery, but should have the same extent as much as possible.
   3. We took a National Hydrology Dataset shapefile of the region, and exported the Chukchi Sea as a single polygon. In step 7, this is used to create ‘distance to ocean’ as an attribute.
2. Add training points. Assign a numeric ‘class’ attribute to each point.
   1. Traditionally this is done with carefully drawn polygons around homogenous areas. We simplify and expedite this process by using points. Training data is captured by intersecting the point ID with the attributes of the segment it falls within.
   2. In ArcCatalog, create a blank point shapefile. Using the ‘Create Features’ sidebar, manually add points where the land cover class is identifiable. Alternatively, random point or grids of points could be generated and then identified.

**01_clip_raster.py**

1. Clip your base imagery to the study area.
   1. ArcMap tools: ‘Clip (Data Management)’, with option ‘use input features for clipping geometry’.
   2. Optional: combine base imagery into a mosaic and export a single raster. This will considerably simplify following steps. We maintained all satellite scenes separately, in order to create scene-specific classifying models. This also allowed tests of all steps to be performed on a smaller raster, reducing computational time for exploratory work.
   3. In QGIS, and occasionally in ArcTools, ‘no data’ values in a raster can cluster with actual data during the segmentation. Depending on the imagery and the extent of your study area, this step should potentially follow segmentation.

**02_segment.py**

1. Segment the base imagery.
   1. Complete a PCA of the bands of the base imagery to determine which 3 bands to use in the segmentation. The available WV2 tiles four our study area were a mix of 8 band and 4 band imagery, with the Red, Green, Blue and NIR bands consistently available. A PCA in R determined that NIR, Red and Green were the least correlated.
   2. Choose several small areas to run tests for different parameters. Segmentation is the basis of the whole process, and it should accurately reflect the variation in the imagery. Running the segmentation, and capturing the attributes for each segment (step 5) are the most computationally intensive steps.
   3. The segmentation parameters in the ArcMap tool ‘Segment Mean shift’ are spectral detail (1-20), spatial detail (1-20) and minimum pixel number. We chose the highest spectral detail (20), moderately high spatial detail (18) and 54 pixels, equivalent to 200 m^2^.
   4. After the parameters have been tested, run segmentation for all satellite scenes.

**03_get_segment_attributes.py**

1. Capture segment attributes for all available datasets. [This step could potentially be done in R, by converting the segmentation to polygons (step 5), and then extracting the data of the base layers using raster::extract and running summary statistics.]
   1. Using ArcMap tool ‘Compute Segment Attributes’, run a python loop to capture attributes from the main imagery, and all other datasets available (elevation, slope, aspect, curvature, etc.) for each satellite scene.
   2. Calculate the base segment attributes of pixel count, rectangularity and compactness in one attribute file, perhaps the main imagery. These are a property of the segmentation and will not vary with different datasets.
   3. For all data, capture mean and SD.
   4. Save output files with scene identifier and dataset names (e.g. scene1_aspect.tif; scene1_elevation.tif; scene1_WV2.tif).
2. Convert the segmented rasters into polygons.
   1. Use the ‘Raster to Polygon’ tool, choose ‘do not simplify’. The polygon boundaries should be the exact pixel boundaries of the segmentation.

**04_capture_polygon_centroids.R**

1. Read polygons into R to calculate centroids and distance to ocean. [This step could be done in ArcMap, but is faster in R, and segues into the data collation and classification process of the next steps.]
   1. Read all polygon files into R.
   2. Read in the ocean shapefile, if distance to ocean is a parameter of interest. Ensure the CRS of polygons and the ocean shapefile is the same.
   3. Calculate centroids, using function ‘gCentroid’ in the rgeos package. [In ArcMap, calculate field geometry of the polygons.]
   4. Get centroid distance from the ocean (‘rgeos::gDistance’). [In ArcMap, export the centroids to a new shapefile and use the ‘Near’ tool.]
   5. Combine the ocean distance and centroid x and y coordinates into a dataframe with the object id of the polygon. Create a SpatialPointsDataFrame with this as data and the centroid coordinates. Write into a point shapefile in the same workspace as the attribute files. This will create a dbf with the ocean distance, latitude and longitude as attributes.

**05_merge_dbf.R**

1. Read all segment attribute dbfs created in step 6 and 7 into R and combine them by scene. [This could be done in ArcMap, using attribute table joins, and then the combined data read into R.]
   1. Have a set workspace with all dbf files. Read them all into R.
   2. Use R tools to combine the data for a given scene by the segment ID.

**06_random_forest.R**

1. Read in the training points shapefile to R. [This and the following steps must be taken in R.]
2. Attach training points to the segmentation. All of these steps are done by each satellite scene, as scenes overlap and but may have different attributes.
   1. Read in the segmented raster tiles.
   2. Segregate the training points by raster. (‘raster::intersect’)
   3. Using ‘raster::extract’, capture the object id of the segment each training falls in.
   4. Remove duplicate object ids. Inevitably, some training points will have been placed in what the segmentation grouped as one polygon.
   5. Bind the training class to the collated information from the dbfs.
   6. All dbf rows with an associated training point are now training data. All unassociated rows are unclassified data.
3. Create a classifying model based on the training data.
   1. Many classifiers are implemented in R. Consider using the ‘caret’ package to test the accuracy and processing speed possible models for a subset of the training data.
   2. We used Random Forest, as implemented in the package ‘randomForest’ (see Methods.)
   3. Models can be created separately for the training data from each scene, or the training data from all scenes combined.
   4. Save the models to disk.

**07_RF_models_compare.R**

1. Compare the out-of-bag error of your models.
   1. Compare the RF out-of-bag errors by class and by satellite scene, or any other factor you can devise.

**08_classify.R**

1. Classify the remaining unidentified data.
   1. Classify each the training data with the model.
   2. Recombine the unknown and training segment data for each scene, with a column for the numeric class. Write dbfs for each scene in a folder with a copy of the segmented file. It does not matter if the dbfs contain all the combined segment attributes, or solely the segment ID and numeric class.
   3. To compare multiple models, write the dbfs in sperate folders.

**09_test_classification_accuracy.R**

1. Test the accuracy of your models.
   1. With a reserved test set of field data, capture the class IDs of the segments they fall on.
   2. Summarize the classification accuracy through confusion matrices for each model, as well as error rates by class and satellite scene.

**10_create_classified_rasters.py**

1. Using ArcMap ‘LookUp’ tool and numeric class as the lookup field, create rasters from the dbf of each segmented scene.
   1. I found it useful to create a colormap file (.clr) and import this to the LookUp rasters for display and comparison of intermediary maps. (**11_add_colormap.py**, optional)

**The following steps are taken manually in ArcMap.**

1. Create a mosaic of all rasters and edit the footprints through homogenous areas to remove abrupt transitions.
2. Export to a final raster or polygon shapefile.
